# Understanding carbon dioxide activation and carbon–carbon coupling over nickel

**DOI:** 10.1038/s41467-019-12858-3

**Published:** 2019-11-25

**Authors:** Charlotte Vogt, Matteo Monai, Ellen B. Sterk, Jonas Palle, Angela E. M. Melcherts, Bart Zijlstra, Esther Groeneveld, Peter H. Berben, Jelle M. Boereboom, Emiel J. M. Hensen, Florian Meirer, Ivo A. W. Filot, Bert M. Weckhuysen

**Affiliations:** 1 Inorganic Chemistry and Catalysis group, Debye Institute for Nanomaterials Science, Utrecht University, Universiteitsweg 99, 3584 CG Utrecht, The Netherlands; 2 Schuit Institute of Catalysis, Department of Chemical Engineering and Chemistry, Eindhoven University of Technology, PO Box 513, 5600 MB Eindhoven, The Netherlands; 3 BASF Nederland B.V., Strijkviertel 61, 3454 PK De Meern, The Netherlands

**Keywords:** Catalytic mechanisms, Heterogeneous catalysis, Density functional theory

## Abstract

Carbon dioxide is a desired feedstock for platform molecules, such as carbon monoxide or higher hydrocarbons, from which we will be able to make many different useful, value-added chemicals. Its catalytic hydrogenation over abundant metals requires the amalgamation of theoretical knowledge with materials design. Here we leverage a theoretical understanding of structure sensitivity, along with a library of different supports, to tune the selectivity of methanation in the Power-to-Gas concept over nickel. For example, we show that carbon dioxide hydrogenation over nickel can and does form propane, and that activity and selectivity can be tuned by supporting different nickel particle sizes on various oxides. This theoretical and experimental toolbox is not only useful for the highly selective production of methane, but also provides new insights for carbon dioxide activation and subsequent carbon–carbon coupling towards value-added products thereby reducing the deleterious effects of this environmentally harmful molecule.

## Abstract (web summary)

Carbon dioxide is a desired feedstock for platform molecules, such as carbon monoxide and higher hydrocarbons, but needs improved catalysts. Here, the authors use a combined theoretical and experimental approach to tune the activity and selectivity of CO_2_ conversion over nickel towards desired products.

## Introduction

As we enter the era of small molecule activation, the main questions to answer for heterogeneous catalyst researchers in the 21st century involve hydrogen (hydrogenation catalysis), and the coupling of carbon fragments^1,2^. As discussed in recent literature^3^, there are CO_2_ mitigation strategies that can and should be applied as soon as possible. Those aimed at point sources of CO_2_ (an example is methanation, which will be introduced below), and those that will play a role in the farther future, i.e., direct air capture (DAC) of CO_2_, and the production of increasingly complex (and thus value added) carbon-containing molecules. However, it is important to stress that the latter processes cannot play a role in maintaining the 1.5 °C global temperature increase mark due to the laws of energy-technology deployment^3,4^.

CO_2_ conversion into CH_4_ (further denoted as CO_2_ methanation) is a process with high technological readiness levels. Its reaction over Ni is the classical reaction discovered by the French chemist Paul Sabatier in the early 1900s^5,6^ (also referred to as the Sabatier reaction) and one that can be applied in the so-called Power-to-Gas (PtG) principle^7–9^. Here, point source CO_2_ emissions can be employed as cheap, or even negative cost carbon feedstock to demodulate the mismatch in renewable electricity demand and supply, while reducing harmful CO_2_ emissions in the atmosphere via a closed-cycle process^10,11^. While hydrogen produced via the electrolysis of water could directly be used as an energy carrier, technical requirements for its large-scale storage deem it an expensive solution for large-scale (seasonal) storage of electricity. For this reason, small molecules, such as ammonia, methanol and methane, are considered for this specific application^8,11,12^. The advantage of methanation is that it can be carried out under relatively mild conditions, that is, starting at atmospheric pressure and between 300 and 450 °C, and is synthesized over the cost-effective non-noble metal Ni^13^. As it is one of the technologies that may be applied in the near future it is of great importance to tune this reaction with practical tools we have at hand. That is, for example by varying the support^14–18^, and by varying particle size^19,20^.

In this work we will use a combination of experiment and theory to determine what the different pathways for carbon dioxide activation are, and how carbon–carbon coupling proceeds on a nickel surface. This knowledge will allow us to not only tune the activity and selectivity of the Sabatier reaction in the Power-to-Gas framework, but also gain fundamental knowledge about some basic principles of heterogeneous catalysis on non-noble metal catalysts. As of yet, to the best of our knowledge, no systematic study has been done to fully catalog and identify experimental and theoretical activity and selectivity descriptors in catalytic CO_2_ methanation over Ni. The ultimate desire to valorize CO_2_ emissions, and to use them as a feedstock even for the production of value-added platform molecules, such as CO or higher hydrocarbons, over non-scarce metals requires the successful combination of theoretical knowledge with materials design.

## Results

### Particle size and support-based tuning of carbon dioxide conversion

We have synthesized a set of well-defined SiO_2_ supported Ni catalysts with particle sizes varying from 1 to 6 nm, and Ni catalysts supported on different metal oxides with differing degrees of reducibility: Al_2_O_3_, CeO_2_, ZrO_3_, and TiO_2_. More details on the characterization results and approach taken can be found in the Supplementary data ‘Catalyst Samples’ (Supplementary Figs. 1–11 and Supplementary Table 1) and literature^19^.

**Fig. 1:**
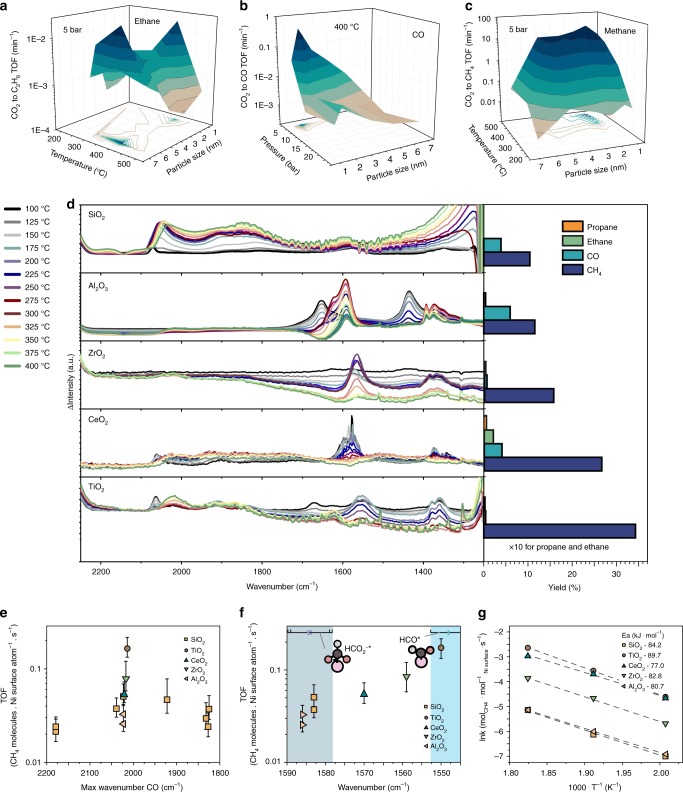
Structure sensitivity and support effects for tuning selectivity and activity in the carbon dioxide hydrogenation over Ni. **a**–**c** The particle size dependence of the turn over frequency (TOF) for CO_2_ hydrogenation towards methane, ethane, and CO over SiO_2_-supported Ni catalysts at 5 bar, with varying temperatures and at 400 °C, with varying pressures. **d** The support dependence of CO_2_ hydrogenation as measured by operando FT-IR spectroscopy from 100 to 400 °C with corresponding yield (measured in a high-throughput reactor system, corresponding well to online values for operando FT-IR spectroscopy) at 300 °C. **e** TOF of CO_2_ methanation at 400 °C plotted against the wavenumber at which the maximum absorbance is observed in the CO region (2200–1800 cm^−1^). Error bars in **e** and **f** are defined as standard deviation in particle size in TOF calculation. **f** TOF of CO_2_ methanation at 400 °C plotted against the wavenumber at which the maximum absorbance is observed in the formate region (1550–1600 cm^−1^) for catalysts showing comparable wavenumber of maximum absorbance in the CO_ads_ region (range: 2019–2023 cm^−1^). The ranges indicated in blue in the plot are the calculated fundamental vibrational frequencies of each most stable adsorbate (of HCO_2_* and HCO*) on Ni(111), with a 5% error bar indicated on the top of the figure. **g** Apparent activation energy barriers calculated from operando FT-IR measurements at different temperatures, for the different supports.

CO_2_ conversion was carried out in different catalytic setups; both by high temperature, high pressure operando FT-IR spectroscopy and GC analysis, and in a multi-tubular reactor system (see section ‘Supplementary data catalyst samples’ and Supplementary Note 1), under a wide range of different reaction conditions (temperatures varying from 200 to 500 °C, and pressures ranging from 5 to 20 bar). As to be expected, methane is the main, or often the only product readily reported for the hydrogenation of CO_2_ over Ni^21,22^. However, measuring under methanation conditions (1:4 CO_2_ to H_2_ at varying pressures and temperatures) and using a high sensitivity operando GC/FT-IR setup, we have also observed the formation of ethane, CO, and (which is rather unusual) low quantities of propane, whereas no alcohols were observed. More ethane forms than propane, and no ethanol forms (Fig. 1).

Firstly, examining the effect of Ni particle size on selectivity more closely, by looking at the turnover frequencies (TOFs) plotted in Fig. 1a–c, ethane formation via C–C coupling seems to be structure insensitive for many of the measured temperatures, i.e., there is no change in surface-normalized activity for different Ni particle sizes. At 400 °C, however, a saddle point in the surface-normalized activity versus Ni particle size is observed. The observed trend is the exact inverse of the observed trends for methane activity at these reaction conditions (shown in the ethane trend in Fig. 1a versus methane in 1c, but also other literature^3,19^). Based on these trends, a maximum in methane activity where there is a minimum in ethane activity, we may conclude that the coupling of C fragments on the Ni surface is competing with the hydrogenation of the C fragments into methane. Furthermore, it is interesting to see that small Ni particles produce more CO (Fig. 1b), which might be explained as too small Ni particles cannot dissociate CO, while CO_2_ must have been activated on them to form CO. Hence likely, the CO hydrogenation step is thus more structure sensitive than the CO_2_ hydrogenation step. CO is unwanted in synthetic natural gas, and we may thus practically tune the selectivity of this reaction, for example, by using slightly larger Ni nanoparticles.

Secondly, examining the effect of catalyst support by studying Ni nanoparticles on different oxides, we observe that the intermediates detected by FT-IR spectroscopy and the product distribution changes with reducibility of the support (see Fig. 1d). Most notably, there is a bathochromic shift in the vibrational frequency of intermediates in the formate region with increased reducibility of the support, and the yields to different end products change for the different supports accordingly.

More specifically, it seems that by delocalizing the electrons around adsorbed CO (e.g. adding support-H* to the adsorbed CO* or CO_2_*), the rate-determining step is affected leading to higher activity (increased yield, e.g. for Ni/TiO_2_). On the other hand, Ni/Al_2_O_3_ is shown to produce CO with high selectivity (93%) at low temperature (200 °C), which opens up possibilities for CO_2_ to syngas conversion. Highly interestingly, propane forms on Ni/CeO_2_ catalysts. CeO_2_ can cooperate with Ni to form CO from CO_2_ via surface vacancies^23^, and thus increases the availability of C–containing intermediates on or near the Ni surface. The effect of different supports deserves further focus in CO_2_ valorization research, especially in terms of the possibility to exploit strong metal-support interactions to expose different active metal sites thus changing the selectivity of supported nanoparticles.

Nevertheless, it is valuable to theoretically understand and therefore better leverage these observed effects. Figure 1e–g summarizes the experimental descriptors we have found in the operando FT-IR spectroscopy experiments (see also Supplementary Fig. 7). We have previously established that there is a CO adsorption strength dependence with particle size, and that this correlates with catalyst activity^19^ (see also Fig. 1e), we now see that the bathochromic shift in the region 1600–1550 cm^−1^ over different supports also correlates with TOF (Fig. 1f). Notably, the shift going from Ni/SiO_2_ to Ni/TiO_2_ is in the order of 30–40 cm^−1^, which is the expected difference between formate (HCOO^−^*) and a formyl (HCO*) adsorbates, as shown by theoretical calculations (see the below). Furthermore, by examining catalyst activity at different temperatures for the different supported catalysts we can see that the apparent activation energy from CO_2_ to CH_4_ is virtually irrespective of the different supports (Fig. 1g). An obvious explanation for this would be that the reactions predominantly take place on the Ni nanoparticles. The effect of the support is then to influence the Ni particle size and shape, which leads to more or less active sites of some sort (disregarding CeO_2_ where we know the interface plays an active role). This will influence the pre-factor, which is the product of the entropic factor in the Arrhenius equation and the amount of active sites. From these experiments, we can take two working hypotheses to evaluate theoretically. Firstly, that the rate-determining step is the hydrogen-assisted dissociation of carbon monoxide. Secondly, that Ni particles with an optimal size (2–3 nm) must have a higher concentration of sites facilitating exactly this reaction step.

### Theoretical calculations explain structure sensitivity in carbon dioxide activation

This experimental information gives us enough input to make approximations to link our experimental descriptors to theoretical descriptors, to ultimately understand and thus be able to further manipulate desired activities and selectivities. To this end, computational catalysis (Density Functional Theory, DFT^24,25^) was applied to understand these concepts. Through calculations, e.g. the application of microkinetic modeling^26^, a detailed study of surface reactions at the molecular level can be made possible^27,28^. A caveat here is that some approximations have to be made. Though the development of force-fields is in full speed as of yet, one simply cannot include support effects to a realistic slab model for large Ni particles. Nickel clusters of a few atoms may be modeled in this way where full shape effects are taken into account. However, it is well-established that particles that are larger than ~1 nm (as used in our experimental studies) are well-described by periodic slabs. In nickel, quantum effects may be expected to influence such an approximation up to ~560 Ni atoms^29–31^, or ~2.5 nm. We compare 3.6–6 nm Ni nanoparticle sizes across the different supports, or ~2570–10,864 atoms. Supplementary Figure 12 and Supplementary Note 2 give more information on DFT modeling of catalytic reactions over nanoparticles. Thus to simulate the availability of different active sites on a Ni nanoparticle (fcc structure), two terrace facets, (Ni(111), and Ni(100)), and two stepped facets, (Ni(211), and Ni(110)) were examined. These facets represent the plethora of available sites on nanoparticles, which can largely be grouped into under-coordinated (stepped) and highly coordinated (terrace) sites (see also Supplementary Fig. 13). This approximation will prove to be justifiable if our theoretical calculations can be linked to our experimentally observed kinetic and vibrational values.

Chemisorption energies (i.e. the energy required to remove an adsorbate in a given position from a surface) give a good indication of the stability of each reaction intermediate. The fundamental vibrational frequencies of HCO_2_* and HCO* thereby obtained, show that the more reducible a support is in our experiments, the more the nature of FT-IR peaks shift towards a HCO* intermediate instead of a HCO_2_*. The nature of this finding can be much better understood in the light of a full mechanistic understanding of processes leading from CO_2_ to CH_4_ over Ni.

**Fig. 2:**
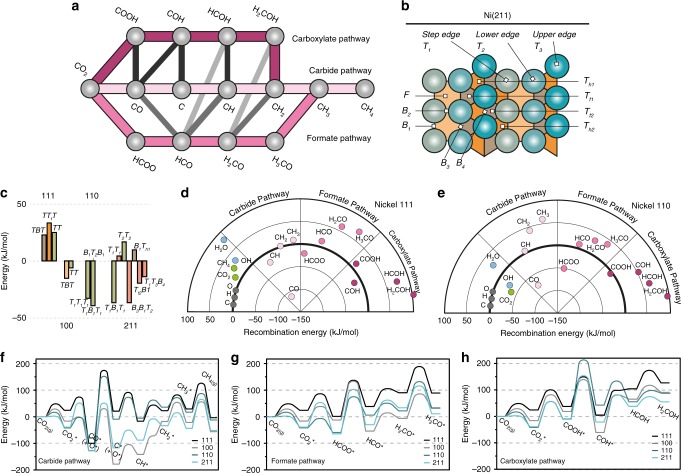
Theoretical calculations of CO_2_ activation over Ni. **a** Schematic including all possible reaction pathways for the carbon dioxide hydrogenation in pink, all intermediates are represented as gray dots. Gray lines indicate plausible links between the primary pathways. **b** Schematic representation of available sites (T_1_, T_f1_, B_1_, …) on a Ni(211) surface. **c** Chemisorption energies of CO_2_ on different available sites of Ni(111), Ni(100), Ni(110), and Ni(211) crystal facets (T: top, B: bridge). **d**, **e** Recombination energies of the carbide, formate and carboxyl pathways on a terrace Ni(111), and a stepped Ni(110) crystal facet. The left panel in each respective figure shows the C, H, and O adatoms from which each intermediate is recombined, as well as reaction intermediates OH and H_2_O. The most stable adsorption site is set at zero-energy. **f**–**h** Potential energy diagrams for (**f**) the carbide pathway (**g**) the formate pathway, and (**h**) the carboxyl pathway on terrace facets Ni(111) and Ni(100), and stepped facets Ni(110) and Ni(211) of CO_2_ hydrogenation as calculated by nudged elastic band (NEB).

Experimental evidence suggests CO_2_ activation proceeds via two parallel particle size-dependent mechanisms over Ni^19^. The first step in the hydrogenation of CO_2_ over Ni is the dissociation of CO_2_ to CO, either via a formate intermediate (formate pathway, Fig. 2a), or directly via adsorbed CO_2_ (CO_2_*). CO can then be further dissociated, or directly hydrogenated to form CH_4_ (carbide pathway, Fig. 2a)^19,32–35^. While these pathways have shown to be active, so-called carboxyl, or alcohol pathways (Fig. 2a) are well known on Ni’s (next) neighbors in the periodic table; Cu and Fe^36^ but have not yet been shown to exist on Ni. Supplementary Table 2 shows all literature values of intermediates from these different proposed pathways, and at the same time illustrates the necessity for an extensive DFT study of methanation over Ni. Supplementary Table 3 shows our calculated most stable chemisorption energy of each of the intermediates shown in Fig. 2a, represented by dots, on each of the 4 Ni facets, and for different adsorption sites (Fig. 2b, Supplementary Fig. 13). Full details (all chemisorption energies and adsorption geometries of all intermediates, on all facets and adsorption sites) can also be found in the Supplementary information (Supplementary Tables 2–4, Supplementary Figs. 14–17). Figure 2c shows the chemisorption energy of CO_2_ on the different facets. The chemisorption energy clearly differs greatly not only per exposed Ni facet, but also for the different adsorption sites on each facet. Stepped surfaces apparently stabilize CO_2_ better than terrace surfaces, which means that the first reaction step, independent of which reaction pathway is followed (carbide, formate, or carboxyl, Fig. 2a), is facet sensitive^19^. A density of states analysis (read below) is further elaborated on in the Methods section and Supplementary information.

When comparing chemisorption energies of a given adsorbate between one facet versus another, comparing less stable fragments (CH*, or CO_2_*) to highly stable fragments such as e.g. C* or O* or e.g. any singular adatom on a Ni(100) surface, because of highly stable hcp hole sites, can be misleading. Therefore, and to obtain a better idea about the possibility of formation of a given intermediate, we compare formation enthalpies starting from singular adatoms, making use of the calculated chemisorption energies, as defined in Eq. 1. Here, *E*_A*_ is the chemisorption energy of reactant A, *E*_A*B*_ that of the formed product and *E*_slab_ is the energy of a bare slab of Ni (with no adsorbates). These “recombination energies” or, the formation enthalpies starting from adsorbed O*, H* and C*, are shown in Fig. 2d, e for Ni(111) and Ni(110), respectively, and in Supplementary Fig. 18 for Ni(100) and Ni(211).

1 $$\begin{array}{l}\Delta H_{nA \ast + mB \ast \to xAB \ast }= \left( {u\left( {E_{{\mathrm{Slab}}} - E_A} \right) + m\left( {E_{{\mathrm{Slab}}} - E_B} \right)} \right) \\ \quad \quad- \left( {xE_{{\mathrm{Slab}}} - E_{AB}} \right)\end{array}$$

From these recombination plots on both a stepped and terrace facet we can draw several conclusions. First, the stability of the first intermediate in each pathway is inversely correlated to the degree of conjugation: in ascending order (CO*, HCOO*, COOH*). Second, we see that the formation of the first intermediate of the carboxyl pathway (COOH*) is thermodynamically unfavorable and each of the following intermediates is generally even higher in energy. This is a good indication of why Ni does not generally form alcohols, as opposed to e.g. Cu or Fe catalysts, which do.

Though Fig. 2d, e give a good overview to compare different reaction pathways, reaction barriers are needed for catalytic understanding. To this end, nudged elastic band (NEB) calculations were performed, which yield activation energies (*E*_a_) for each respective reaction step. Figure 2f–h show the potential energy diagram for each reaction pathway and each examined facet (Supplementary Fig. 19 lists the values for all forward- and backward-activation barriers). From the potential energy diagrams, of the three pathways, the carbide pathway is the lowest in energy. The activation energy of the CO_2_ dissociation step (CO_2_* → CO* + O*) in this lowest-energy pathway vary greatly over the different facets, with no trend in terrace vs. stepped facets (Fig. 2f–h, depicted in detail in Supplementary Fig. 20). The *E*_a_ is in the order Ni(100) < Ni(110) < Ni(111) < Ni(211).

**Fig. 3:**
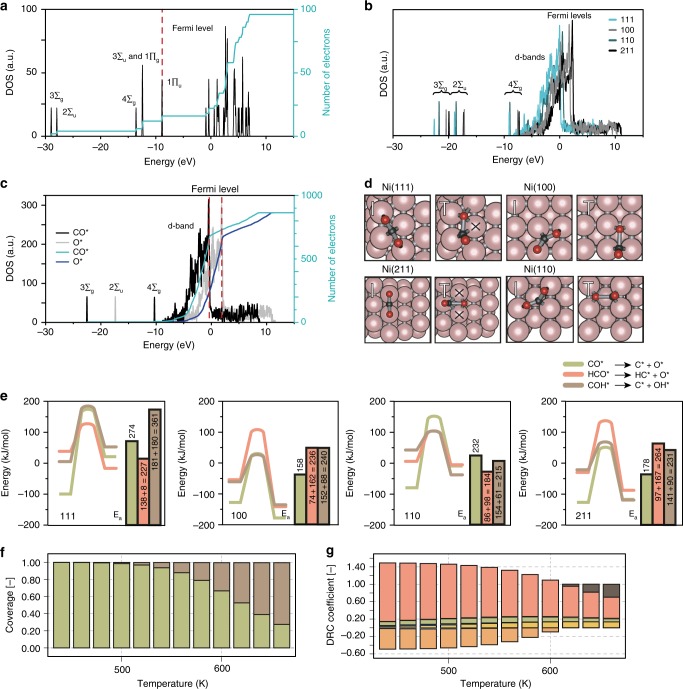
CO_2_^ǂ^ transition state analysis and microkinetic modeling show the effect of H assistance on CO* dissociation. **a** The density of states (DOS) and integrated DOS of CO_2(g)_. Each peak corresponds to a molecular orbital. **b** DOS analysis of transition state CO_2_^ǂ^ in CO_2_^*^ → CO* + O* on Ni(111), Ni(100), Ni(110), and Ni(211). **c** DOS of the final state of CO_2_* → CO* + O* on Ni(100). **d** Geometries of CO_2_ on four nickel facets in the initial and transition state for direct CO_2_ dissociation on the four Ni facets. Shared atoms are indicated by a cross. **e** Energy barriers of (H-assisted) CO dissociation on Ni(111), Ni(100), Ni(110), and Ni(211). **f**, **g** Microkinetic modeling of individual reaction steps on the Ni(110) surface in a temperature range from 440 to 660 K. **f** Coverage as a function of temperature (green, CO; gray, empty adsorption site*) **g** degree of rate control (DRC) coefficients (red, HCO* + * ↔ CH* + O*; green, COH* + * ↔ CO* + H*; orange, OH* + H* ↔ H_2_O* + *; yellow, CH_2_* + H* ↔ CH_3_ + *; gray, CO_2_* + H* ↔ COOH* + *).

To obtain a descriptor for the facility of CO_2_ activation, a highly important reaction step in any CO_2_ utilization process^37,38^, an in-depth density of states analysis (DOS) was performed. Supplementary Figure 21 shows the initial - transition - and final states of the elementary reaction steps relevant in CO_2_ hydrogenation. To activate a C–O bond in CO_2_*, we understand (e.g. from the molecular orbital (MO) diagram and DOS analysis in Fig. 3a, and Supplementary Figs. 22 and 23) that donation into 4Ʃ_g_* should be considered. Yet (as Fig. 3b, and Supplementary Fig. 22 show) there is proper overlap between the anti-bonding orbitals of the adsorbate (CO_2_*) with the d-orbitals of each metal facet under study and thus it seems that a decrease of the bond-order of CO_2_* should be equally facile on all Ni facets. Nevertheless, by comparing the position of the Fermi-level corrected MO’s of CO_2_^ǂ^ in Fig. 3b, (here ^ǂ^ denotes the transition state in CO_2_* → CO* + O*, more information in Supplementary Fig. 23 and Supplementary Table 5), we have a tool to rank nickel facets in order of activating effect per MO. For 2Ʃ_u_ the order is 111 < 110 < 211 < 100. From a DOS analysis of CO* and O* (depicted in Fig. 3c and Supplementary Figs. 24–27), we understand that MO 2Ʃ_u_ is separated from 4Ʃ_g_ and 3Ʃ_g_ to form CO* and O*, respectively. We now understand why Ni(100) activates CO_2_ the best (in CO_2_* → CO* + O*): it activates the 2Ʃ_u_ molecular orbital the best.

Nevertheless, other factors should be taken into account to fully describe the trend in activation of CO_2_* for all four facets under study. When considering the geometric aspects of CO_2_^ǂ^ on each surface, see Fig. 3d (and Supplementary Fig. 28, Supplementary Table 6 for more information), the final descriptor is found; i.e. the transition state (TS) stability due to the bond-order conservation (BOC) principle stating that a transition state with fewer shared metal atoms yields increased TS stability and thus a lower E_a_. While on Ni(100) and Ni(110) the TS shares no metal atoms, it shares 1 and 2 atoms on Ni(111) and Ni(211) respectively, corresponding well to the second highest and highest *E*_a_, in direct CO_2_ dissociation. We now understand exactly how CO_2_ is activated over Ni. Furthermore, in Fig. 3f, g we see that the *E*_a_ for CO* → C* and HCOO* → HCO* are the highest in each respective reaction pathway. This is in very good agreement with experimental observations where CO* and HCOO* are continuously observed in operando studies of CO_2_ hydrogenation over Ni^19,34^. However, the highest energy barrier is actually in the lowest-energy pathway (the pathway with the most stable intermediates), namely the carbide pathway (CO* → C*). This is not an uncommon occurrence and it is believed to exist also in e.g. the Fischer-Tropsch Synthesis (FTS) process over Co^26^ or CO methanation over Ni^39^. It is thus interesting to study this behavior for Ni from a fundamental point of view^26,40^.

From experimental studies we know that high surface coverage of CO* is often observed under methanation conditions^19,34^. To circumvent the high activation energy barrier of CO* → C*, H-assisted CO dissociation via either the formate pathway (CO* → HCO* → CH*) or via the carboxyl pathway (CO* → COH* → C*) may occur. As Fig. 3e shows, the overall reaction barrier (2 steps for H-assisted CO dissociation; the addition of H to CO, and the ultimate cleavage of O) can be lower than the direct dissociation of CO.

A detailed microkinetic model, which can be constructed using the theoretical input described above, yields insights into the transient chemical behavior of the chemical system. This can give important details such as the rate limiting step, most abundant reaction intermediate, and *E*_a_ ultimately linking theory and experiment. Details on the microkinetic model can be found in the Supporting information (section ‘Microkinetic modeling’, Supplementary Figs. 29–40 and Supplementary Tables 7–8). The outcome yields some important conclusions. Firstly, that the most methane is produced on Ni(110) facets (in the order 110 > 211 > 111 > 100). Figure 3f, g show the coverage of intermediates, and the rate of CH_4_ production on Ni(110) as a function of temperature. Secondly, that the most abundant reaction intermediate for the most active facets (Ni(110), Ni(211), and Ni(111)) is CO and that the rate limiting step is its (hydrogen assisted) dissociation, on Ni(100) it is H_2_CO* (Supplementary Figs. 29–40 and Supplementary Tables 7 and 8). An optimum in the Ni particle size, with sufficient terrace facets to supply H* and sufficient step sites with highest activity, is thus theoretically expected; and experimentally observed. These conclusions confirm our experimental findings and show that the assumptions made for our theoretical model hold valid.

This information we have now obtained (the highest *E*_a_ is the direct dissociation of CO* to C*, and that an excellent way to circumvent this is by the addition of H to the adsorbed CO*) might be leveraged by introducing more reducible supports. These, at the nickel-support interface, can increase the activation of CO* via e.g. the addition of H* from the support. This relates back to experiments in Fig. 1, where the observed experimental descriptors for catalytic activity are both linked to an optimal degree of CO bond activation (Fig. 1e), and to the bond order of HCO(O)* intermediates (Fig. 1f).

**Fig. 4:**
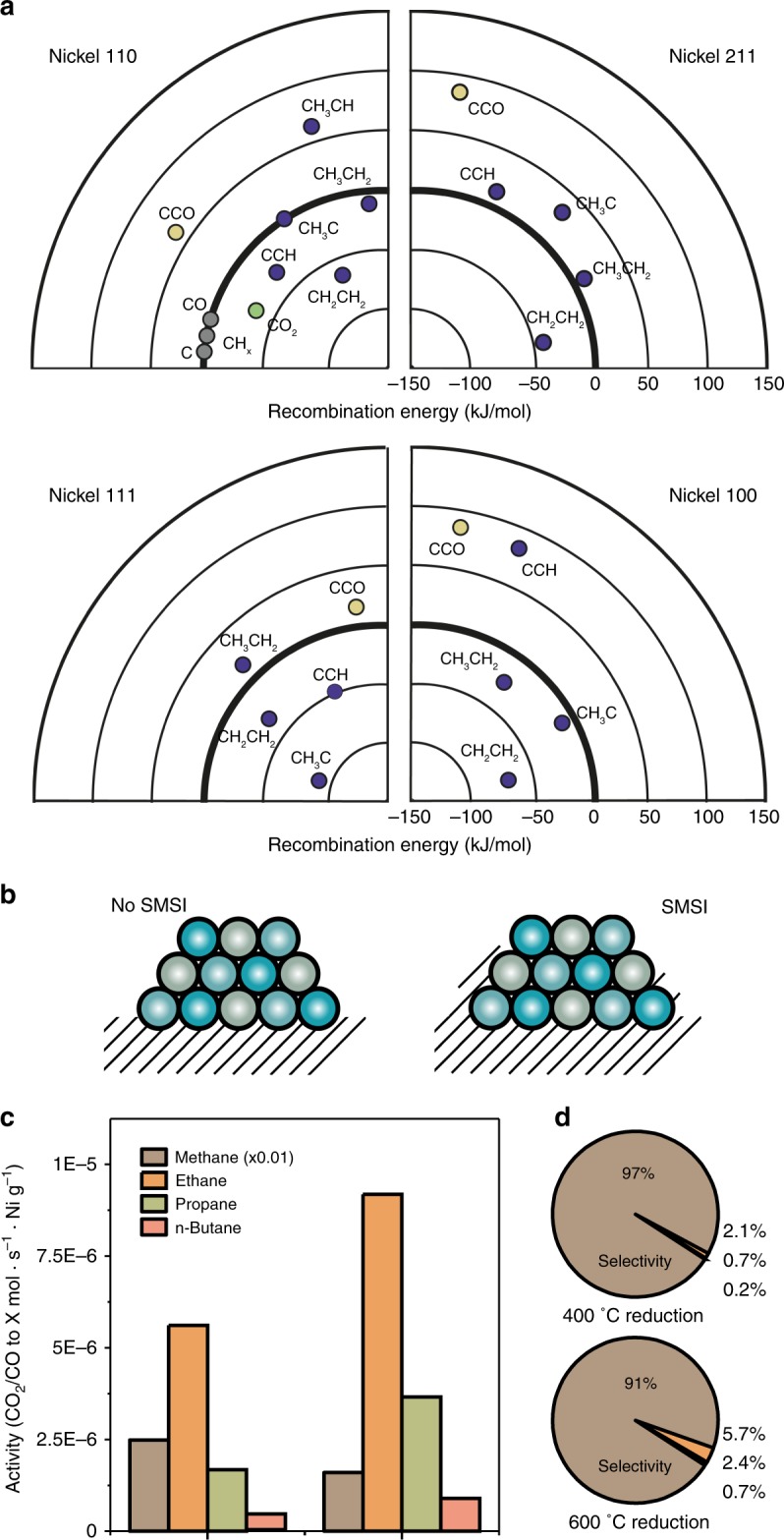
Strong metal-support interaction (SMSI) induced C–C coupling in CO_2_/CO hydrogenation over Ni. **a**, **b** Recombination energies relevant for C–C coupling and CO insertion, on terrace Ni(111), Ni(100), and stepped Ni(110), Ni(211) crystal facet. **b** Schematic showing supposed SMSI effects on a nanoparticle. Sub-oxides are believed to creep up over nanoparticles, affecting their exposed surface area and electronic structure. **c** Activity, **d** selectivity of a 6 wt% Ni/TiO_2_ catalyst reduced at 400 and 600 °C in CO_2_/CO (3:1) hydrogenation at 300 °C at 4 bar.

From literature we also know that ethane formation does occur (albeit in very small quantities) for CO_2_ methanation over Ni^21^, and we have shown in Fig. 1 that C–C coupling (yielding C_*n*>1_ hydrocarbons, e.g. ethane, and even some propane) does occur and seems to be structure insensitive or hydrogenation-rate limited (Fig. 1d). Carboxyl formation (e.g. methanol or ethanol) is not reported in literature for silica-supported Ni catalysts to the best of our knowledge. The concept of CO insertion is interesting thus, assuming here that any alcohol that would be formed is not formed via the carboxyl pathway described in Fig. 2a, as intermediates in this pathway are not stable on Ni (Fig. 2d, e, and Supplementary Figs. 18–20) and the pathway is high in energy (Fig. 2f–h). The recombination energies for the coupling of carbon on Ni via CH_x_–CH_x_* fragments and CO* insertion in C* are given in Fig. 4a. CO insertion is endothermic (i.e., thermodynamically unfavorable) on each surface from theory, thus now we are fully able to explain why Ni, in principle, does not form alcohols. C–C coupling, on the other hand, is favorable in many cases and does not seem to have any preference for terraces or facets. Thus, the only reason for the ever-reported high methane selectivity for Ni catalysts should indeed be a very high relative hydrogenation rate, which correlates to the inverse trend to methane TOF at 400 °C we observed in Fig. 1a. Accordingly, Ni/CeO_2_ catalysts, on which H spillover from Ni is known to occur^41^, showed the most C–C coupling activity.

In a showcase to leverage this gained knowledge from experiments and theory (that C–C coupling over Ni is likely to be hydrogenation rate limited and structure insensitive), trying to make even more C–C coupled products, one may wish to cover part of the Ni nanoparticles, and have sufficient CO already present for the carbide mechanism to be initiated. The former, in turn, can be done by inducing strong metal-support interactions (SMSI), believed to occur when reducible supports are partially reduced^40^. By doing so, sub-oxide support species creep up over the nanoparticle affecting the exposed surface, and consequently the electronic structure (Fig. 4b). In Fig. 4c–d this effect is shown using a 6 wt% Ni catalyst supported on TiO_2_. When the catalyst is reduced at 600 °C, versus at 400 °C, we see increased C–C coupling and even formation of butane, while methane production decreases (Fig. 4c, d). This bolsters the experimental and theoretical findings presented here as suppressing the hydrogenation activity (for which methane production is a qualitative measure) through SMSI increases C–C coupling.

## Discussion

In the valorization of CO_2_ towards platform molecules, we need to improve current catalytic systems. Classical single-metal catalyst systems are often still not fully mapped in terms of theoretical and experimental descriptors for activity. By evaluating the two main parameters at hand to tune the electronic and geometric properties of classical single-metal heterogeneous catalytic systems, a broad understanding can be developed through which new systems may be designed. Electronic and geometric effects can be tuned by two main ways; structure sensitivity, and support. It is apparent from the results presented in this work that, neglecting the individual contribution of supports that can inherently activate CO_2_, the apparent active reaction pathway is very similar across different supports and particle sizes. Another conceptually important conclusion is that suppressing the hydrogenation activity of Ni leads to C–C coupled products. Selective suppression of hydrogenation activity is arguably the biggest challenge in catalysis research in the activation of small molecules, one of the most pressing fields in the clean energy transition.

In this work we have shown that via a complete theoretical mechanistic understanding of CO_2_ hydrogenation over a classical monometallic catalyst system, Ni on silica, we are entering the era of rational design and are able to tune the selectivity of CO_2_ conversion experimentally. These concepts are important not only in the selective production of methane e.g. in the Power-to-Methane concept, but also for the ultimate valorization of CO_2_ as a feedstock for value-added chemicals, such as higher hydrocarbons, aiding in the mitigation of its harmful effect on the environment. At the same time, we stress the reality that to overcome the challenges in small molecule activation, classical heterogeneous catalytic systems will not suffice. Rather, nanostructuring, bifunctionality, or for example functional ligands should be introduced for which studies such as the current could form a theoretical fundament.

## Methods

### DFT calculations

All DFT calculations were performed using the Vienna Ab-initio Simulation Package (VASP)^24,42^ with the projector-augmented wave (PAW) method^42,43^. The Perdew-Becke Ernzerhof (PBE) exchange-correlation functional was used^44^. The bulk lattice constant of nickel (face centered cubic crystal structure) was optimized, yielding a value of 3.521 Å, which corresponds to the experimental bulk lattice constant of 3.5 Å. The four nickel facets were modeled with this optimized Ni-Ni distance using periodic boundary conditions. All atoms were allowed to relax. The kinetic energy cutoff for the plane wave basis set was 400 eV. The Monkhorst-Pack mesh k-points of (5 × 5 × 1) for Ni(111), Ni(100) and Ni(110) and (3 × 3 × 1) for Ni(211) were used. Between each slab a 10-Å vacuum, perpendicular to the surface between the layers, was applied in order to prevent interaction of intermediates between a bottom-side and a top-side of overlying super cells. Dipole-dipole interactions between super-cells was avoided by bifacial adsorption of intermediates at the surface working from a center of inversion. All recombination energies shown in the main text and supporting information have thus been corrected for by dividing the outcome of Eq. 1 by 2. An energy criterion was used for the ionic convergence using the conjugate gradient algorithm. Geometries were converged to 10^−4^ eV and electronic convergence was set at 10^−5^ eV. For the gas-phase calculations of CO_2_, CO, H_2_, and H_2_O only a G centered grid for k-point sampling was used. The molecules were placed at the center of a 10 × 10 × 10 Å unit cell. For electron smearing, a Gaussian smearing with a width of 0.00002 eV was used. The reaction pathways discussed in the main text (Fig. 1) have been calculated by the nudged elastic band (NEB) approach as implemented in VASP^45,46^. A frequency analysis was performed to confirm that all transition geometries were in a first-order saddle point on the potential energy surface. The Hessian matrix was constructed using a finite displacement approach with a step size of 0.02 Å for displacement of individual atoms along each Cartesian coordinate. These frequencies were used to determine the zero-point energy (ZPE) correction to the energy of the geometries of the initial, transition, and final states.

### Catalyst synthesis and characterization

Silica-supported Ni catalysts were prepared by homogeneous deposition precipitation (HDP) over silica gel (Sigma Aldrich, 150 A 200–425 mesh, S.A. = 325 m^2^/g). Further information can be found elsewhere^19^. Ni catalysts supported on TiO_2_ (Degussa P25, S.A. = 60 m^2^/g), CeO_2_ (prepared by precipitation with urea, vide infra), Al_2_O_3_ (Puralox TH 100/150, S.A. = 196 m^2^/g) and ZrO_2_ (precipitation, vide infra) were prepared following a similar method using urea as a precipitating agent. The Ni loading was fixed at 6% for all catalysts. To obtain a g of final catalyst, 297.27 mg of Ni nitrate hexahydrate (99.999%, Sigma Aldrich) and 1.6 g urea (p.a., Acros) were dissolved in 50 mL water, followed by addition of 940 mg of the selected support. The system was then vigorously stirred at 95 °C for 20 h, to induce hydrolysis of urea and deposition of Ni^47^. Finally, the system was centrifuged and washed with water until the pH of the supernatant was neutral. The obtained powder was dried at 120 °C for 24 h, and reduced in-situ at 450 °C before CO_2_ hydrogenation catalytic experiments, based on Temperature Programmed Reduction (TPR) results (Supplementary Fig. 5).

CeO_2_ and ZrO_2_ supports were prepared by a similar, scaled up procedure. In a typical synthesis, 50 g of cerium(III) nitrate hexahydrate (99.99% trace metals basis, Sigma Aldrich) or 25 g of zirconium(IV) oxynitrate hydrate (99.99% trace metals basis, Sigma Aldrich) were dissolved in 1.7 L of DI water together with 750 g urea. In the case of Zr, the solution was heated to 60 °C to allow for complete dissolution of the precursor. The solution was then brought to 95 °C for 20 h, and the precipitate was then filtered and washed with water until neutral pH. The obtained cake was dried at 60 °C for 12 h and at 120 °C for 24 h, followed by calcination to 500 °C for 5 h, 5 °C/min ramp, under static air. The obtained CeO_2_ and ZrO_2_ powder had a surface area of 88 m^2^/g and 68 m^2^/g.

The reduced Ni supported catalysts were examined with transmission electron microscopy (TEM) in an FEI Tecnai T12 operated at 120 kV or in an FEI Tecnai F20 operated at 200 kV. Samples were crushed and suspended in ethanol under ultrasonic vibration. A drop of this suspension was deposited on a holey carbon film on a 300 mesh copper grid. TEM images are reported in Supplementary Fig. 6.

## Supplementary information

Supplementary Information

## Data Availability

All relevant data is available from the authors.

## Code availability

All relevant code not already in the open literature can be requested from the authors.
