# Supplementary material for: Understanding carbon dioxide activation and carbon–carbon coupling over nickel
Source: Nat Commun. 2019 Nov 25;10:5330. doi: 10.1038/s41467-019-12858-3 (PMC6877608; doi:10.1038/s41467-019-12858-3)
Supplement: Supplementary file 1 — Supplementary Information [file 41467_2019_12858_MOESM1_ESM.pdf]

Supplementary Information to: Understanding carbon dioxide activation and carbon-carbon coupling over nickel

Vogt et al.

Supplementary information

Supplementary data catalyst samples	3
DFT calculations	13
Chemisorption energies	16
Stable geometry figures	24
Density of states analysis CO_2* → CO* + O*	54
Microkinetic modelling	63
Supplementary references	76

Supplementary data catalyst samples

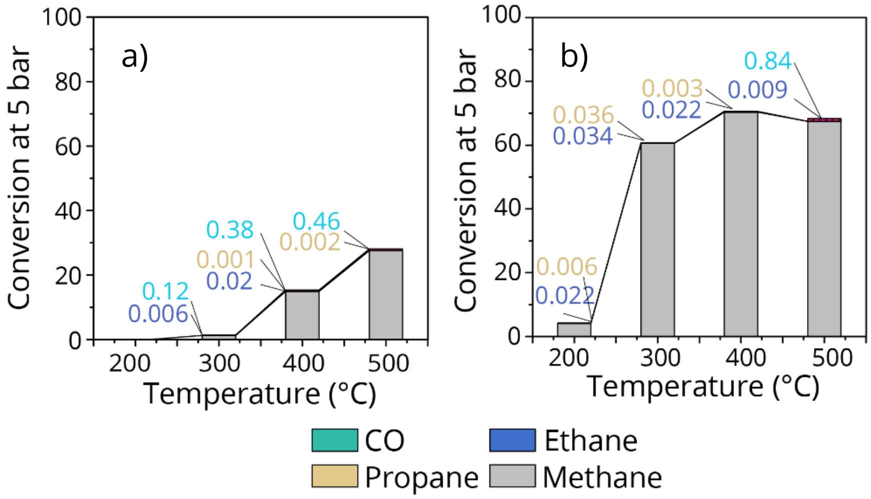

Supplementary Figure 1. Yield of methane, ethane and propane at 5 bar for the catalyst samples with the smallest and the largest mean Ni diameters (catalyst samples a) 1 and b) 6).

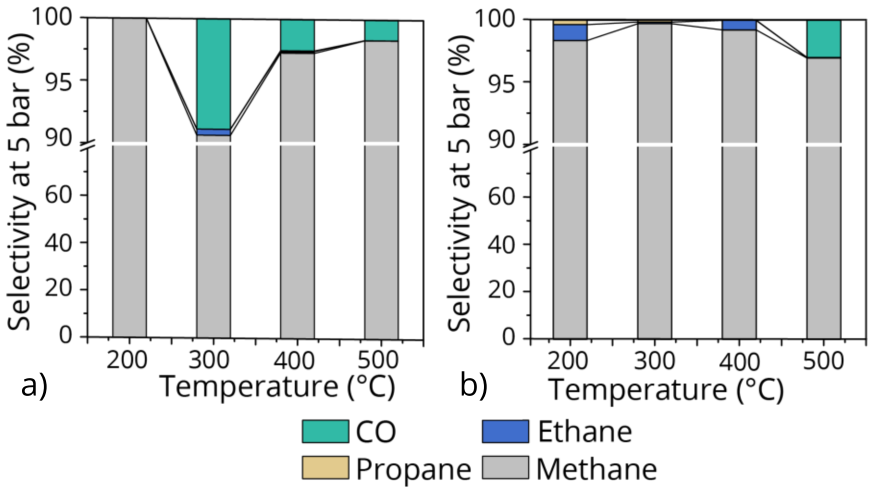

Supplementary Figure 2. Selectivity towards methane, CO, ethane and propane at 5 bar for the catalyst samples with the smallest and the largest mean Ni diameters (catalyst samples a) 1 and b) 6).

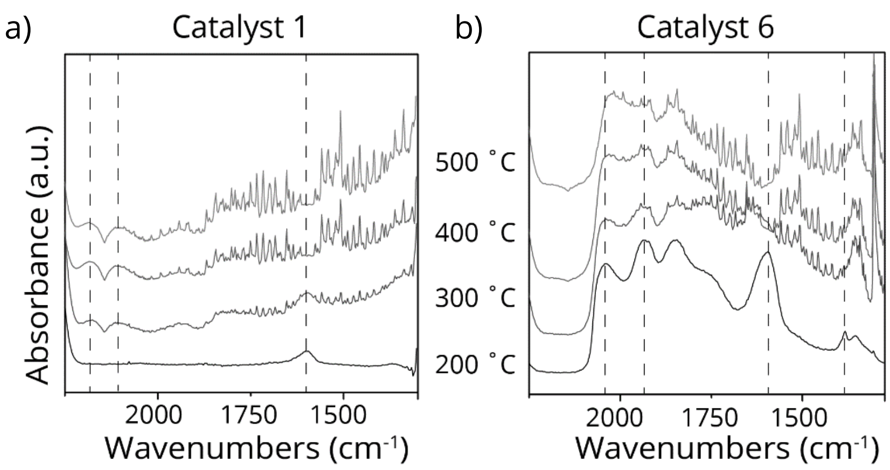

Supplementary Figure 3. Operando FT-IR spectra recorded during CO_2 hydrogenation experiments with a catalyst sample with Ni mean particle size of 1.4 nm (catalyst 1), and of 6.0 nm (catalyst 6) at 5 bar and 200, 300, 400 and 500 °C.

Supplementary Note 1

To supplement the activity data provided via operando FT-IR as is described in the main text, and to verify whether the side products (particularly ethane and propane) could be duplicated in a different catalytic setup, the catalysts were tested in a classical fixed bed multitubular reactor system. The catalysts were tested at atmospheric pressure, and at 200, 300, 400 and 500 °C. It is important to note here that the ethane and propane results could be reproduced despite having measured at atmospheric pressure.

It is known that the effect of a support is generally more intense than any differences induced purely based on site composition (induced e.g. by changes in nanoparticle size). Nevertheless, both support- and size-effects will contribute to designing the overall most active catalyst. Here we separate the effects of site compositions from support effects as the complete reaction mechanisms on Ni should be calculated first, after which the effect of the support may be examined and compared in a separate study. This basic but broad understanding of a study on nickel alone already allows us to rationally design Ni catalysts with reducible supports, to facilitate the CO hydrogenation step. While we do not yet include the effect of support in our DFT calculations; we show that it is something that should be examined in the future and report the case against which such calculations should be compared. For the catalyst samples on different supports shown in the main text the apparent nanoparticle size is not the same on each different support (varying from 2 to 8 nm). Yet observed differences in terms of activity and selectivity are one order of magnitude more intense than what was observed on Ni/SiO_2 samples having different particle sizes[1], as discussed above. The effect of the support on the catalytic performance is predominant over nanoparticle size; it is thus interesting to study the effect of these different supports on different particle sizes and their activity in the future e.g. once appropriate mesoscale DFT forcefields have been developed.

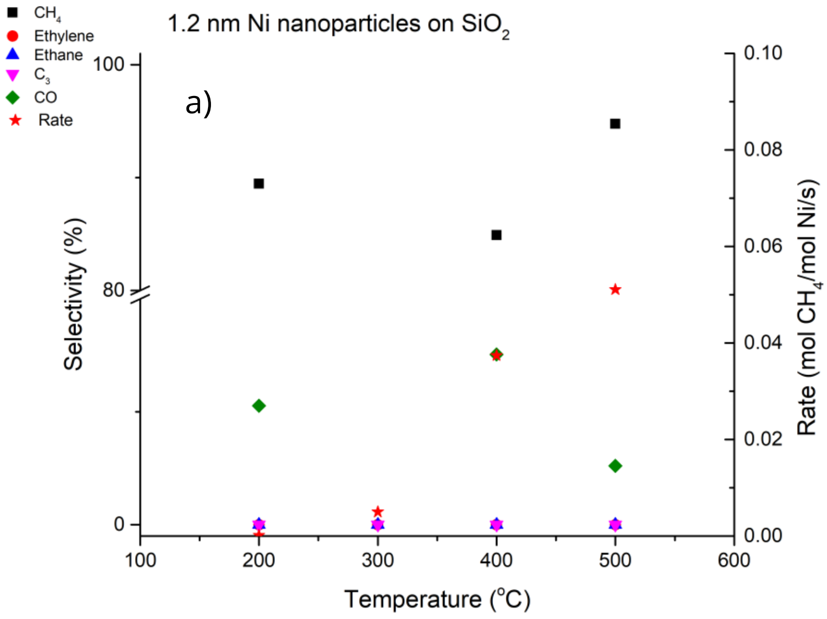

1.2 nm Ni nanoparticles on SiO_2
a)
CH_4
Ethylene
Ethane
C_3
CO
Rate
Selectivity (%)
Rate (mol CH_4/mol Ni/s)
Temperature (°C)

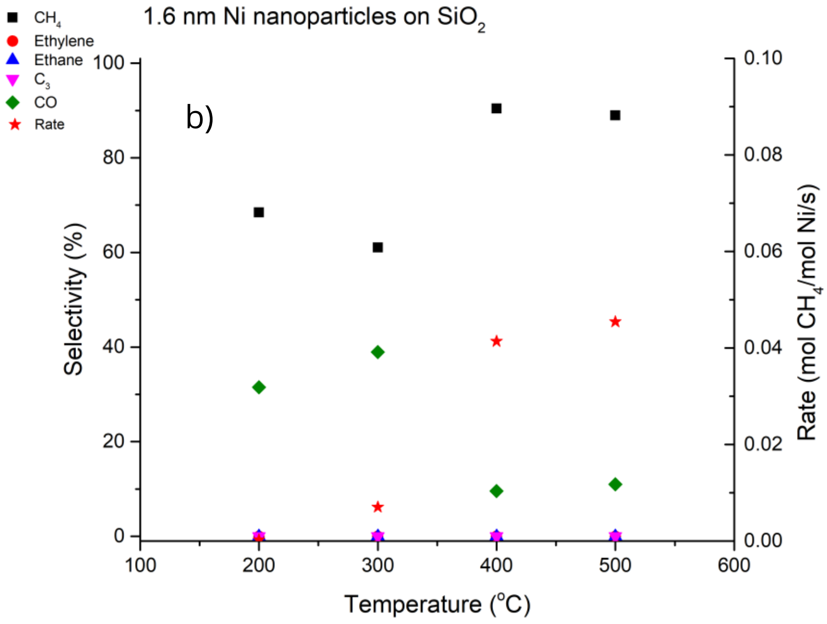

1.6 nm Ni nanoparticles on SiO_2
b)
CH_4
Ethylene
Ethane
C_3
CO
Rate
Selectivity (%)
Rate (mol CH_4/mol Ni/s)
Temperature (°C)

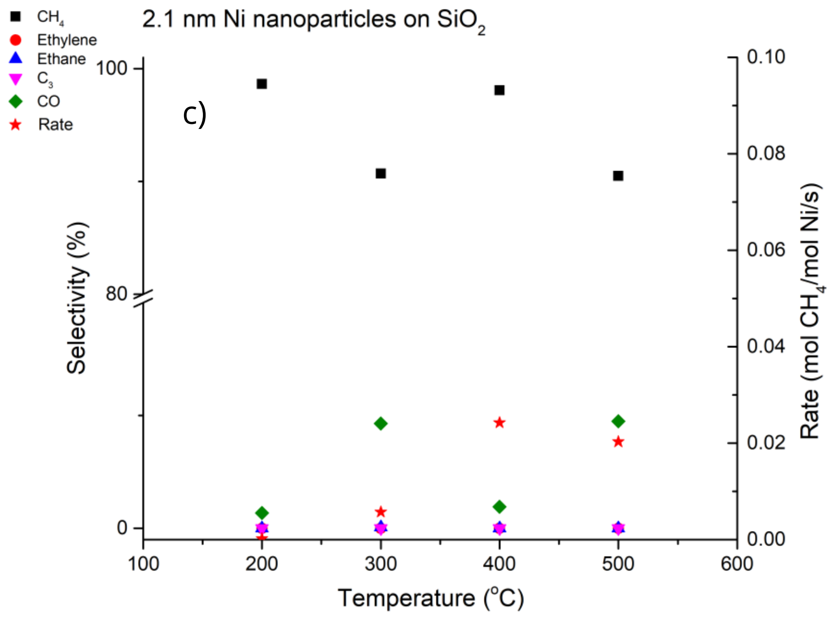
2.1 nm Ni nanoparticles on SiO2
CH4
Ethylene
Ethane
C3
CO
Rate
c)
Selectivity (%)
Rate (mol CH4/mol Ni/s)
Temperature (°C)
100
80
0
100
200
300
400
500
600
0.10
0.08
0.06
0.04
0.02
0.00

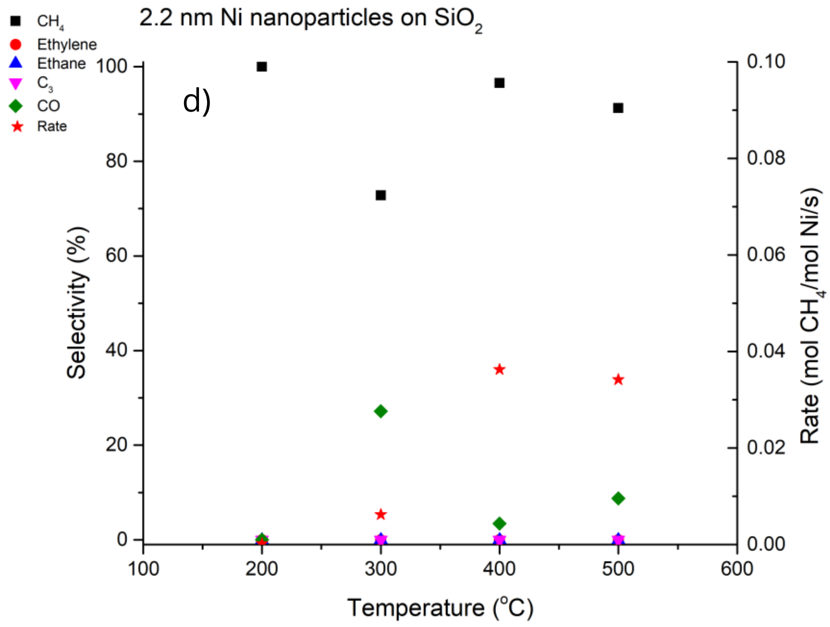
2.2 nm Ni nanoparticles on SiO2
CH4
Ethylene
Ethane
C3
CO
Rate
d)
Selectivity (%)
Rate (mol CH4/mol Ni/s)
Temperature (°C)
100
80
60
40
20
0
100
200
300
400
500
600
0.10
0.08
0.06
0.04
0.02
0.00

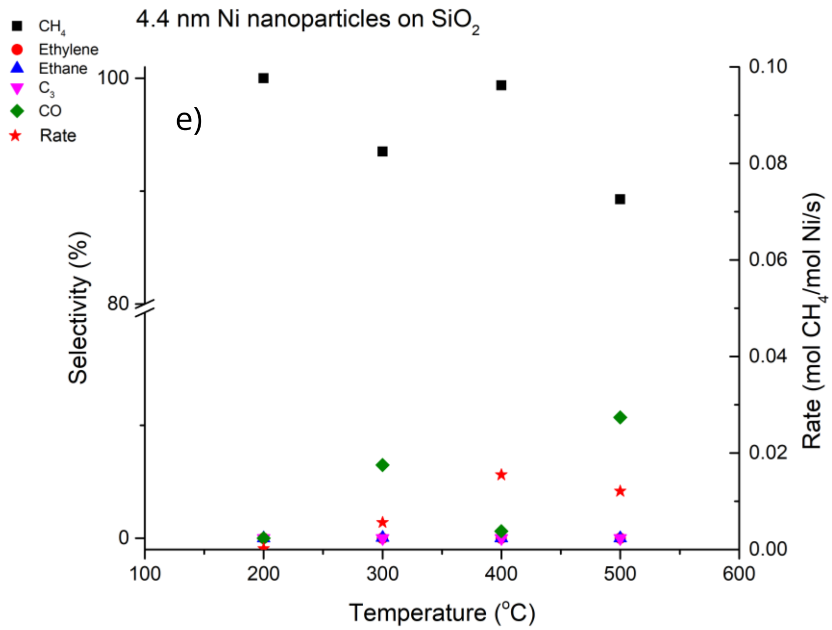

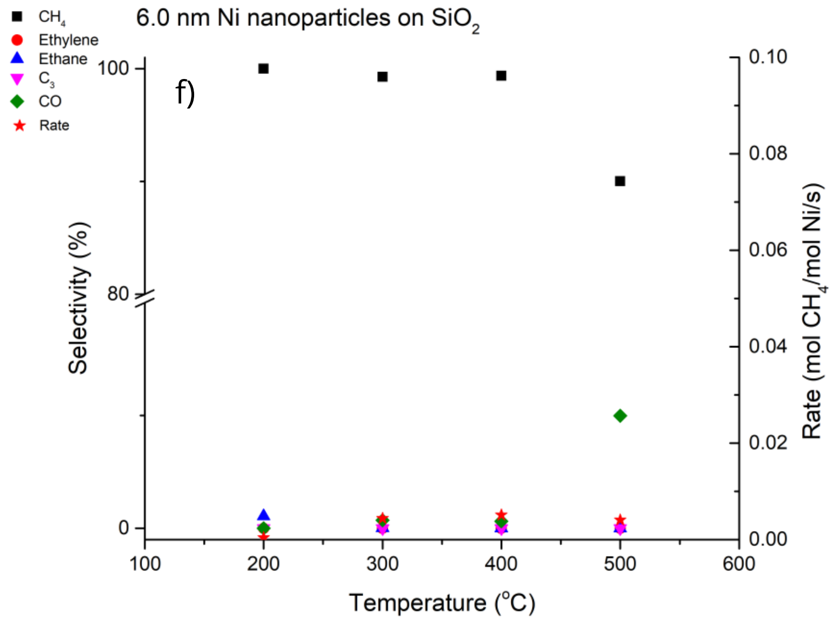

Supplementary Figure 4. Selectivity to all measured end products, and rates of methane formation in CO_2 hydrogenation over SiO_2 supported Ni catalyst samples 1-6 (a-f) tested in a classical fixed bed reactor setup.

Supplementary Data Varied Supports

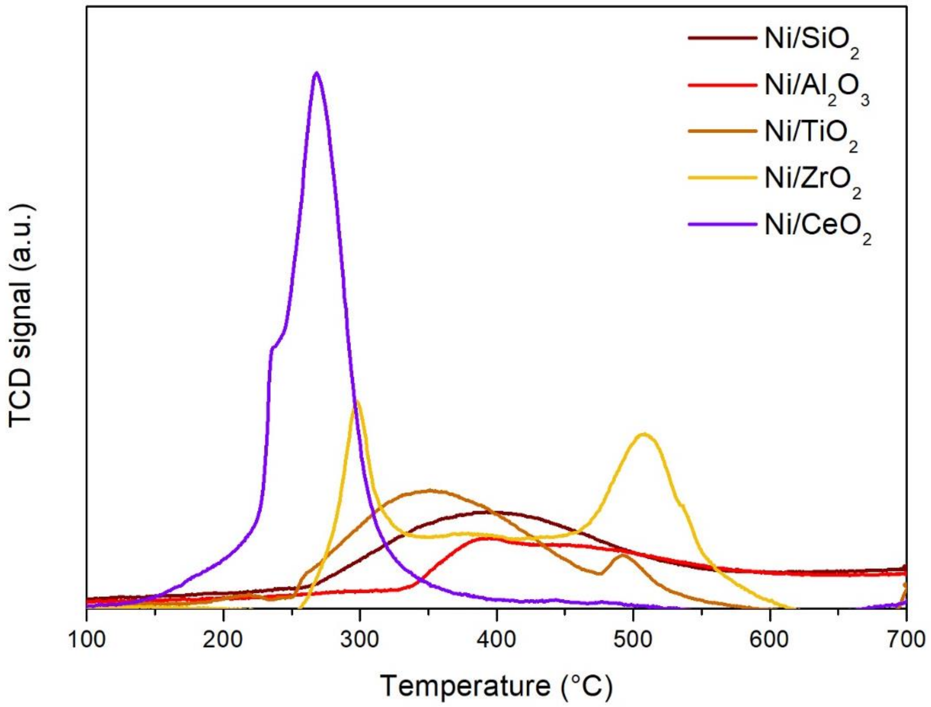

Supplementary Figure 5. TPR profiles of the supported Ni catalyst samples.

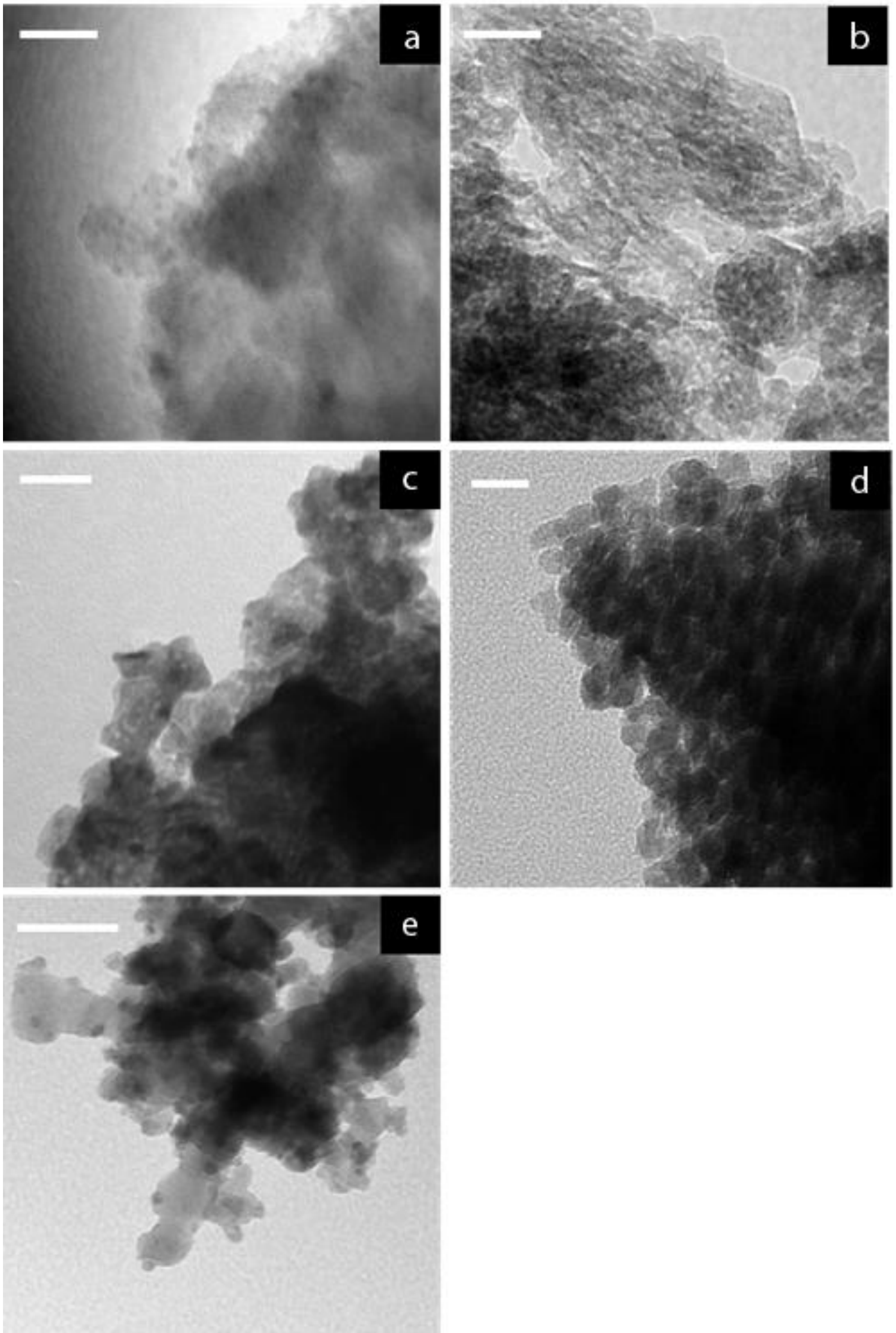

Supplementary Figure 6. Representative TEM images of reduced (a) Ni/SiO_2, (b) Ni/Al_2O_3, (c) Ni/CeO_2, (d) Ni/ZrO_2, (e) Ni/TiO_2. Scalebars are 20 nm.

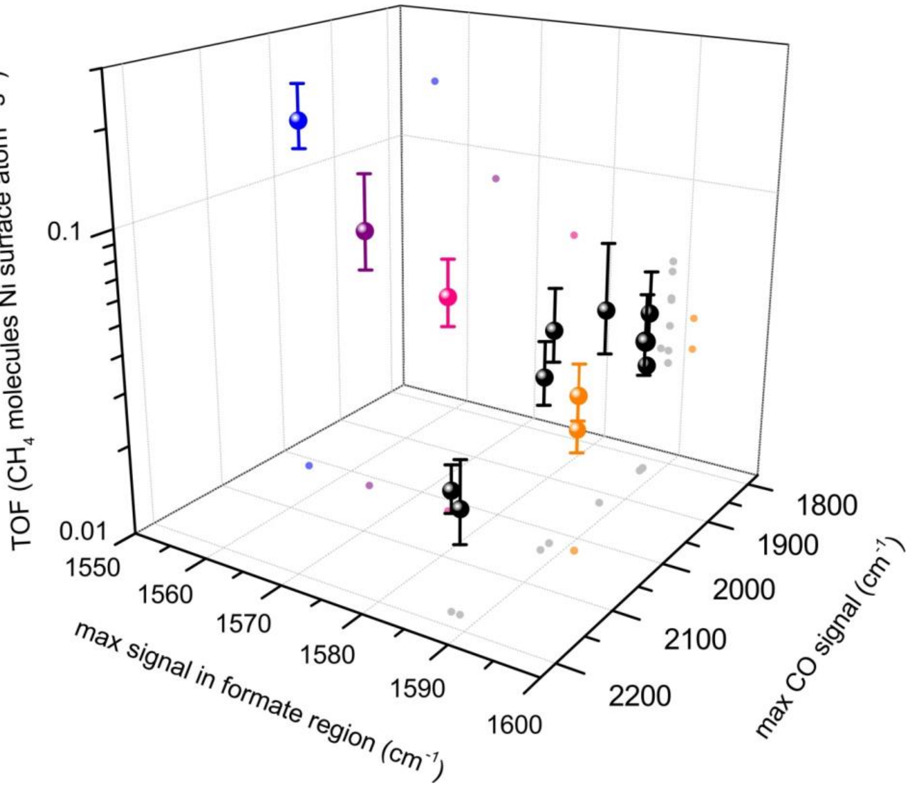

Supplementary Figure 7: TOF of CO_2 methanation at 400°C plotted against the wavenumber at which the maximum absorbance is observed in the $CO_{(ads)}$ (2225-1800 cm^{-1}) and formate regions (1550-1600 cm^{-1}) over Ni/Al_2O_3 (orange), Ni/SiO_2 (black), Ni/CeO_2 (pink), Ni/ZrO_2 (purple) and Ni/TiO_2 (blue). Projections on the XY and XZ axes are shown for clarity. Error bars were calculated based on the standard deviation of nanoparticle size distribution in TEM analysis.

Supplementary Table 1: Nickel loading and particle size analysis results for the studied samples.

Sample	Ni loading by ICP(wt.%)	Ni TEM mean particle size after reduction (nm)
Ni/SiO_2	6,7	2 ± 0,8
Ni/SiO_2	5,0	1,4 ± 0,4
Ni/SiO_2	1,7	1,4 ± 0,4
Ni/SiO_2	60	6 ± 1,9
Ni/SiO_2	6,2	2,3 ± 0,6
Ni/SiO_2	2,0	2 ± 0,5
Ni/SiO_2	1,0	1,5 ±0,2
Ni/SiO_2	0,5	1,3 ±0,4
Ni/Al_2O_3	2,8	1,3 ±0,2
Ni/Al_2O_3	5,6	5,3 ± 1,1
Ni/CeO_2	6,0	3,6 ± 0,9
Ni/ZrO_2	5,9	6 ± 2,1
Ni/TiO_2	6,0	5 ± 1,2

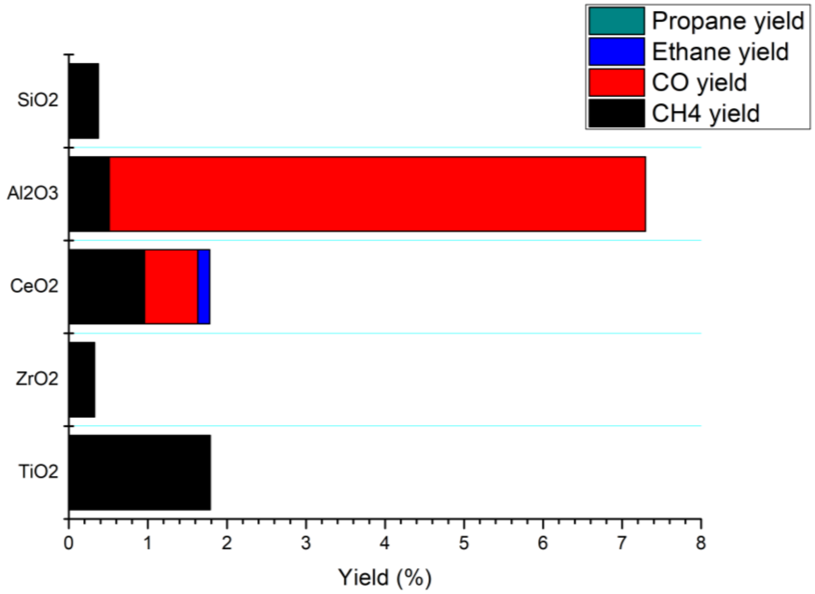

Supplementary Figure 8. Conversion of CO_2 hydrogenation over Ni on varying supports at 200 °C.

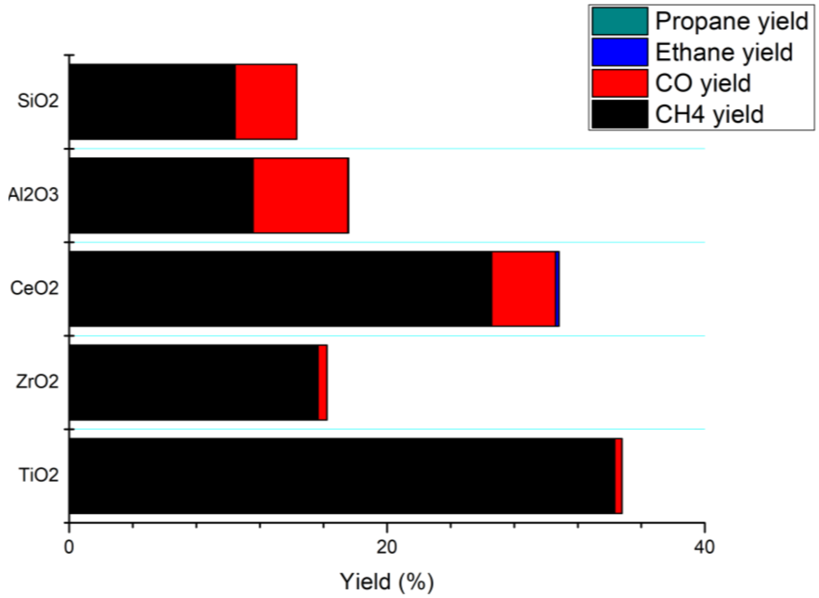

Supplementary Figure 9. Conversion of CO_2 hydrogenation over Ni on varying supports at 300 °C.

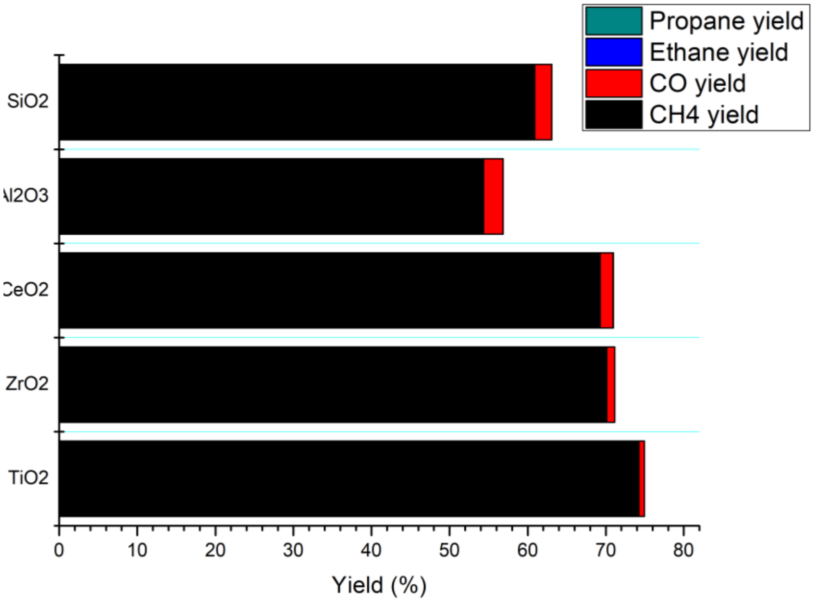

Supplementary Figure 10. Conversion of CO_2 hydrogenation over Ni on varying supports at 400 °C

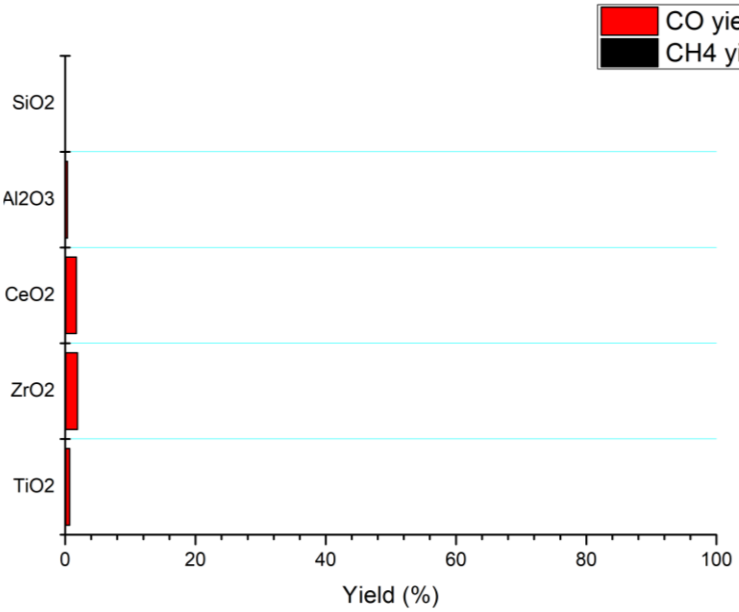

Supplementary Figure 11. Conversion to CO and CH_4 of bare supports at 400 °C. At lower temperatures (200, 300 °C) no activity was detected.

DFT Calculations

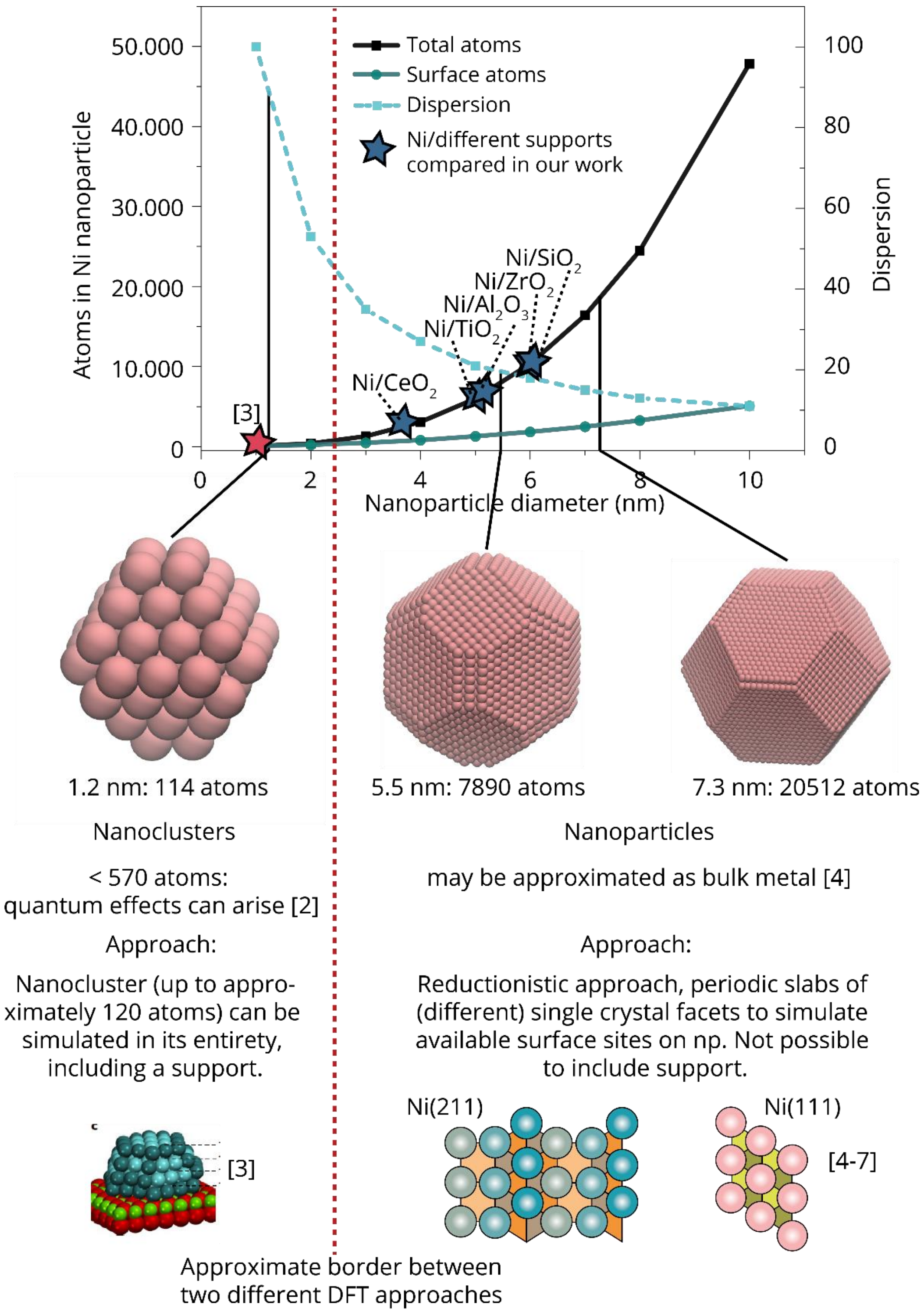

Supplementary Figure 12. Schematic explaining the periodic slab approximation approach and the state-of-the-art in DFT modelling of catalytic reactions on nanoparticles.

Supplementary Note 2

There are currently two approaches to perform DFT modelling of catalytic reactions on nanoparticles. The first is to directly model all atoms of a nanocluster, in this way the support can be included. An example is reference [3], indicated by the red star in the graph in Supplementary Figure 12. This approach is suitable only for nanoclusters as computational power currently simply does not stretch beyond modelling approximately 120 atoms this way. The second approach is to model a nanoparticle (larger than approximately 570 atoms, where no quantum effects are expected) by use of periodic slabs. As our experimental study concerns larger (supported) metal we use theoretical methods that allow to make the connection between theory and experiment.

Nevertheless, one could argue that the support oxide affects the reaction mechanism. For this reason, we had experimentally measured the apparent activation energy across the different supports, which turn out to be nominally the same. This implies that support oxides may change the shape of the metal nanoparticle (and thus the pre-factor, the combination of the entropic term in the Arrhenius equation and the concentration of active sites), but not the mechanism of CO_2 activation over Ni. In other words, we show that our models are in agreement with the theoretical and experimental observations, which serves to prove that the approximation we make, approaching our experimental system by use of periodic Ni slabs, the current state of the art, leads to useful experimental and theoretical descriptors.

Nickel facets and available sites

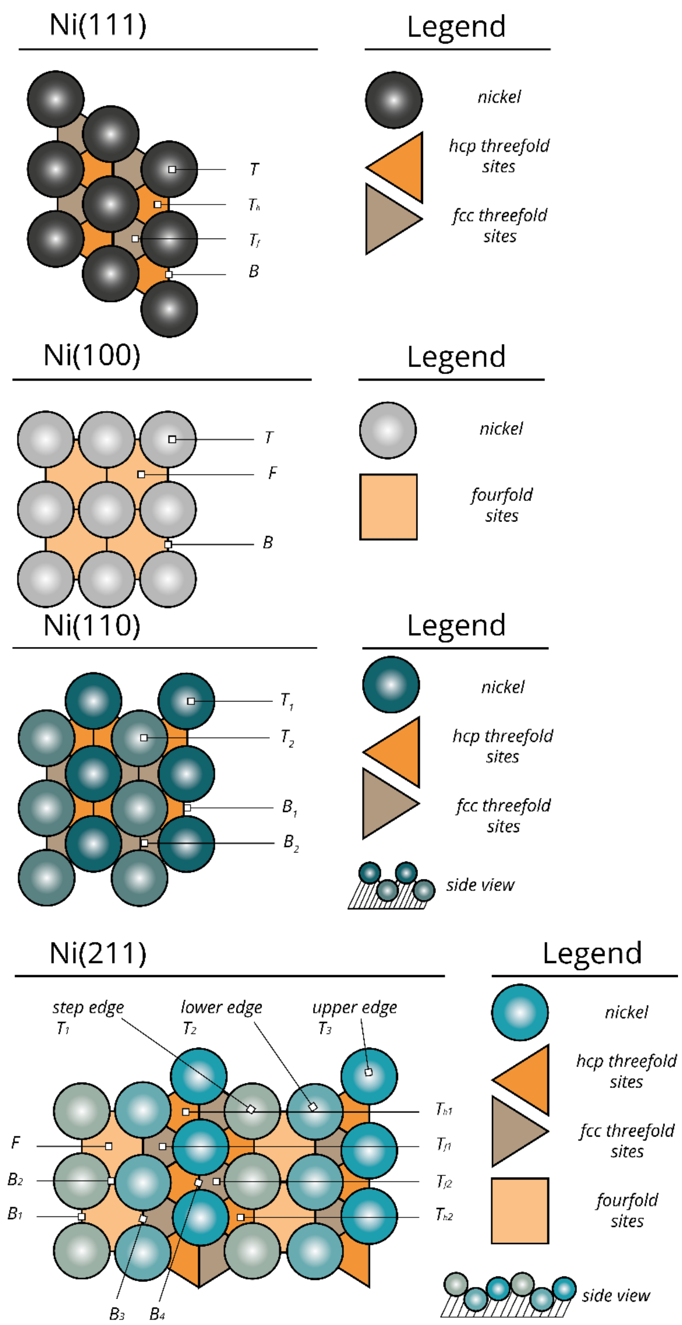

Supplementary Figure 13. Facet Ni(211) is shown in Figure 2B of the main text, the other facets that were calculated, i.e. Ni(111), Ni(100), and Ni(110), and their respective available adsorption sites and the abbreviations thereof are shown in Supplementary Figure 13.

Chemisorption energies

Supplementary Table 2. Calculated chemisorption energies (in eV) of the adsorbate species relative to the respective gaseous species compared to found literature values. The reader is referred to the literature references for details on reaction energy, and activation barrier values.

	Ni(111)	Literature	Ni(100)	Literature	Ni(110)	Literature	Ni(211)	Literature
CO_2	0.25	0.26[8]<br>0.24 [9]	-0.17	-0.14 [8]	-0.43	-0.42 [8]<br>-0.32 [10]	-0.41	-0.42 [11]<br>-0.47 [12]
CO	-1.85	-1.92,<br>-1.91[8]	-1.81	-2.04 [8]<br>-1.88 [13]	-1.81	-1.94 [8]<br>-1.91[14]	-1.93	-1.64 [15]<br>-2.15 [16]<br>-1.97 [13]
HCO	-2.09	-2.26 [17]	-2.74	-3.14 [18]	-2.84	-2.60,<br>-2.91[14]	-2.57	-2.34 − -2.98 [12]
COH	-4.25	-4.39[17], -4.42 [19]	-4.64	-4.68 [20]	-3.93	-4.01[14]	-4.31	-3.94 [15]
HCOO	-2.87	-3.02 [19]	-2.74	-	-3.40	-	-3.12	-3.07 [19]<br>-3.03 -3.07 [12]<br>-3.49 [21]
H_2O	-0.23	-0.20 [22]<br>-0.27 [13]	-0.32	-0.27 [22]<br>-0.36 [13]	-0.40	-0.39 [22]	-0.50	-0.55 [13]

Supplementary Table 3. Overview of most stable adsorption geometry of each intermediate and its respective chemisorption energy for the carbide, formate and alcohol pathway to CO_2 methanation over Ni(111), Ni(100), Ni(110) and Ni(211) sites. Side and top views of the adsorption geometries over the different facets can be found in Supplementary Figure 13.

Adsorbate	Site	Adsorption Geometry	Energy (kJ/mol)
Ni(111)			
OCO	T, B, T	O_1 top, C bridged, O_2 top	24.3
CO	T_h	C threefold hcp	-178.6
CH	T_f	C threefold fcc	-603.3
CH_2	T_f	C threefold fcc	-454.2
CH_3	T_f	C threefold fcc	-238.3
HCOO	T, T	O_1 top, O_2 top	-277.3
HCO	B, T	C bridged, O top<br>*above T_f*	-201.2
H_2CO	T_f , T	C threefold fcc, O top	-57.7
H_3CO	T_h	O threefold hcp	-218.0
COOH	B, T	C bridged, O top	-210.9
COH	T_f	C threefold fcc	-410.5
HCOH	B	C bridged	-270.8
H_2COH	B, T	C bridged, O top<br>*above T_h*	-146.7
C	T_h	C threefold hcp	-773.8
O	T_f	O threefold fcc	-692.4
H	T_f	H threefold fcc	-360.9
H_2O	T	O top	-22.5
OH	T_f	O threefold fcc	-364.9
H_2	B	H_2 vertical above bridge	11.3
Ni(100)			
OCO	T, B, T	O_1 top, C bridged, O_2 top<br>*span a corner*	-16.3
CO	F	C fourfold	-174.8
CH	F	C fourfold	-667.6
CH_2	B	C bridged	-436.8

CH_3	B	C bridged	-233.8
HCOO	T, T	O_1 top, O_2 top<br>*diagonally above F*	-293.8
HCO	B, B	C bridged, O bridged<br>*above F*	-264.6
H_2CO	B, B	C bridged, O bridged<br>*above F*	-127.9
H_3CO	F	O fourfold	-251.9
COOH	T, T	C top, O top<br>*above B*	-232.9
COH	F	C fourfold	-447.5
HCOH	B	C bridged	-285.2
H_2COH	B	C bridged	-159.4
C	F	C fourfold	-909.4
O	F	C fourfold	-724.2
H	F	C fourfold	-364.5
H_2O	T	O top	-31.1
OH	F	O fourfold	-388.4
H_2	T	*H_2 horizontal above top*	-28.5
Ni(110)			
OCO	T_1, T_1, T_1	O_1 top, C top, O_2 top<br>*step edge; span a corner*	-41.9
CO	B_1	C bridged	-174.5
CH	B_l	C long bridged	-613.7
CH_2	T_2	C top<br>*lower edge*	-452.9
CH_3	B_1	C bridged	-252.2
HCOO	T_1, T_1	O_1 top, O_2 top<br>*step edge; long bridged*	-327.8
HCO	B_1,B_1	C bridged, O bridged<br>*above bridged*	-239.6
H_2CO	B_1, T_f	C bridged, O threefold fcc<br>*long bridged*	-190.3
H_3CO	B_1	O bridged	-259.5
COOH	T_1, T_1	C top, O top<br>*step edge; long bridged*	-248.1
COH	B_2	C bridged	-378.9
HCOH	B_1	C bridged	-317.3
H2COH	T_1, T_1	C top, O top<br>*step edge; above B_1*	-173.6
C	B_2	C bridged	-836.1
O	T_h	O threefold hcp	-676.4
H	B_2	H bridged	-351.6
H_2O	T_1	O top	-38.5
OH	B_1	O bridged	-398.6
H_2	T_1	*H_2 horizontal above top*	-62.4
Ni(211)			
OCO	T_1, B_1, T_1	O_1 top, C bridged, O_2 top<br>step edge	-39.1
CO	T_{h2}	C threefold hcp	-186.7
CH	F	C fourfold	-634.2
CH_2	T_{h2}	C threefold hcp	-467.8
CH_3	T_{h2}	C threefold hcp	-265.6
HCOO	T_1, T_1	O_1 top, O_2 top	-340.6

		step edge	
HCO	T_{f1}, B_3	C threefold fcc, O bridged	-248.2
H_2CO	T_{f1}, B_3	C threefold fcc, O bridged	-90.3
		C double bonded to the surface	
H_3CO	B_1	O bridged	-269.1
COOH	B_1, T_1	C bridged, O top	-257.0
		step edge	
COH	F	C fourfold	-415.6
HCOH	B_1	C bridged	-314.4
H_2COH	B_1	C bridged	-192.6
C	F	C fourfold	-874.1
O	T_{h2}	O threefold hcp	-711.4
H	T_{f2}	H threefold fcc	-363.7
H_2O	T_1	O top	-48.5
OH	B_1	O bridged	-415.0
H_2	T_1	*H_2 horizontal above top*	-38.9

Supplementary Table 4. Overview of all other calculated chemisorption energies for all found stable geometries.

Adsorbate Ni(111)	Adsorption Site	Energy (kJ/mol)
CO_2	B, T, T	35.5
	T, T	26.6
	T_f, T, T	24.3
CO	T_h	-178.6
	T_f	-178.1
	B	-158.8
	T	-144.3
CH	T_f	-603.3
	T_h	-600.5
CH_2	T_f	-454.2
	T_h	-449.7
CH_3	T_f	-238.3
	T_h	-235.8
HCOO	T, T	-277.3
	T_f	-224.3
	T_h	-218.1
HCO	B, T *above T_f*	-201.2
	B, T *above T_h*	-200.8
	T	-165.9
H_2CO	T_f, T	-57.7
	T_h, T	-57.3
H_3CO	T_h	-218.0
	T_f	-206.4
COOH	B, T *above T_f*	-210.9
	T, T	-210.8
	B, T *above T_h*	-209.5
	T, T	-188.7
COH	T_h	-410.5
	T_f	-410.4
HCOH	T_h	-270.8
H_2COH	B, T *above T_h*	-146.7
	B, T *above T_f*	-145.5
C	T_h	-773.8
	T_f	-770.0

O	T_f	-692.4
	T_h	-680.9
H	T_f	-361.7
	B	-361.6
	T_h	-360.9
	T	-310.6
H_2O	T	-22.5
OH	T_f	-364.9
	T_h	-356.2
	B	-352.1
H_2	*Above B*	11.6
	Above T	11.6
CCO	T_f	-575.5
	T_h	-568.4
CCH	T_f, T_h	-590.0
	T_h, T_f	-589.2
	T_f	-510.0
	T_h	-503.7
CCH_3	T_h	-572.8
CH_2CH_2	T_f, T	-72.8
CH_2CH_3	B	-182.4
Ni(100)		
CO_2	B, T, T	-16.3
	T, T	-6.5
CO	F	-174.8
	B	-166.3
	T	-147.6
CH	F	-667.6
CH_2	B	-436.8
CH_3	B	-233.8
HCOO	T, T *above F*	-293.8
	B, B	-292.6
	T, T *above B*	-257.8
	F	-240.9
HCO	B, B	-264.6
H_2CO	B, B	-127.9
H_3CO	F	-251.9
COOH	T, T	-232.9
	T, T	-207.9
COH	F	-447.5
	B	-375.1
HCOH	B	-285.2
H_2COH	B	-159.4
C	F	-909.4
O	F	-724.2
H	F	-364.5
	B	-350.5
	T	-311.6
H_2O	T	-31.1
OH	F	-388.4
	B	-381.6
H_2	*Horizontal above T*	-28.5
	Vertical above T	-0.3
	Vertical above B	1.5
CCO	F, T	-636.1
CCH	F, B	-652.7

CCH_3	F	-615.0
CH_2CH_2	B, T	-79.7
CH_2CH_3	B	-200.0
Ni(110)		
CO_2	T_1, T_1, T_1	-41.9
	T_2, B_1, B_1	-38.9
	B_1, T_1, T_1	-35.0
CO	B_1	-174.5
	T_1	-157.3
	B_2	-145.6
CH	T_1, T_1	-613.7
	T_2	-600.7
	B_1	-522.7
CH_2	T_2	-452.9
	B_1	-442.2
CH_3	B_1	-252.2
HCOO	T_1, T_1 *above lower edge*	-327.8
	T_{1-1}, T_{1-1} *(2 times bifold-bound to T_1 sites, see Fig. S4)*	-251.8
	T_{1-1}	-229.3
HCO	T_f, B_1	-239.6
	T_2,T_{1-1}	-217.2
	B_1	-206.5
	T_{1-1}, B_1	-200.8
	T_f, B_1	-176.0
H_2CO	T_f, B_1	-190.3
	B_1, T_f	-164.3
	T_{1-1}, T_{1-1}	-138.1
H_3CO	B_1	-259.5
	T_{1-1}	-211.6
COOH	T_{1-1} *above lower edge*	-248.1
	T_{1-1}	-247.7
	T_{1-1}	-229.6
	T_{1-1}	-224.6
COH	B_2	-378.9
	B_1	-362.0
	T_1	-251.3
HCOH	B_1	-317.3
	T_{1-1}	-250.0
H_2COH	T_1, T_1	-173.6
	B_1	-166.9
C	B_2	-836.1
	T_2	-819.0
	B_1	-699.6
O	T_f	-676.4
	B_1	-671.2
	T_2	-654.5
H	B_2	-351.6
	B_1	-349.1
	T_2	-341.6
	T_1	-308.8
H_2O	T_1	-38.5
OH	B_1	-398.6
	T_{1-1}	-358.1
H_2	*Horizontal above T_1*	-1.2
	Vertical above T_1	1.3
	Vertical above B_1	0.4

	Vertical above T_2	-62.4
CCO	T_2, B_1	-1715.8
	B_2, T_1	-1702.0
	B_1, T_1	-1628.3
CCH	T_f, B_1	-283.4
	B_2, B_1	-254.4
	B_1, T_1	-172.2
CCH_3	T_2, T_1	-555.7
$CHCH_3$	$T_{2\text{-}1\text{-}1}$ *(threefold-bound to T_2 and 2x T_1 site)*	*
CH_2CH_3	B_1	-220.1

* $CHCH_3$ isomerizes to CH_2CH_2

Ni(211)

CO_2	B_1, T_1, T_1	-39.1
	B_1 B_3, T_2	-39.0
	T_{f2}, B_1	-20.9
	T_{h1}, T_1, B_4	-14.8
	T_1, T_1	4.6
	B_1, T_f	10.3
	T_2, T_2	17.8
CO	T_{h2}	-186.7
	B_1	-182.5
	T_{f2}	-177.2
	T_{h1}	-172.9
	T_1	-165.0
	T_{f1}	-160.3
	F	-159.2
	B_2	-154.2
CH	F	-634.2
	T_{h2}	-603.0
	T_{f2}	-592.2
	T_{h1}	-590.3
CH_2	T_{h2}	-467.8
	T_{f2}	-443.9
	T_{h1}	-438.8
CH_3	T_{h2}	-265.6
	T_{f2}	-258.8
	T_{h1}	-211.6
HCOO	T_1, T_1	-340.6
	T_1, T_2	-301.6
	B_1 *above upper edge*	-278.6
	T_3, T_3	-269.0
	B_1 *above lower edge*	-264.2
	T_{f2}	-224.8
HCO	T_{f1}, B_1	-248.2
	T_{h2}, B_1	-237.0
	$T_{1\text{-}2\text{-}3}$, B_1 *(C threefold-bound to T_1 and T_2 and T_3 site)*	-227.4
	T_{h2}, T_1	-227.2
	B_2, B_1	-224.2
	B_2, B_2	-219.2
H_2CO	$T_{2\text{-}3}$, B_1	-90.3
	T_1, $T_{1\text{-}3}$	-86.6
	B_1, B_2	-71.2
	T_3, $T_{3\text{-}1}$	-70.7
	T_3, B_2	-55.2
H_3CO	B_1	-269.1
	T_{h2}	-256.1

	T_{f2}	-233.2
	B_2	-208.2
	T_{h1}	-204.7
	T_{f1}	-187.7
COOH	B_1, T_1	-257.0
	T_1, T_1	-229.3
	$T_{3\text{-}1}$, T_3	-202.5
COH	F	-415.6
	T_{h2}	-413.0
	T_{f2}	-401.0
	T_{h1}	-397.8
	T_{f1}	-388.0
	B_1	-374.1
	B_2	-362.0
HCOH	B_1 *OH down*	-314.4
	B_1 *OH up*	-302.9
	T_{f2}	-270.4
	T_2	-267.9
	T_{h1}	-262.2
	$T_{2\text{-}3}$	-255.1
H_2COH	B_1	-192.6
	T_1T_1	-189.5
	$T_{h1}T_3$	-137.0
C	F	-874.1
	T_{h2}	-786.8
	T_{f1}	-762.1
	T_{h1}	-761.1
O	T_{h2}	-711.4
	T_{f2}	-686.5
	F	-680.7
	T_{h1}	-663.4
	T_{f1}	-661.8
H	T_{f2}	-363.7
H_2O	T_1 *H's above lower edge*	-48.5
	T_1 *H's above step edge*	-41.0
	T_1 *H's above upper edge*	-40.3
	B_1	-37.2
	T_3 *H's above lower edge*	-27.4
	T_3 *H's above upper edge*	-14.1
OH	B_1 *H above lower edge*	-415.0
	B_1 *H above upper edge*	-412.5
	T_{h2}	-388.0
	T_{f2}	-363.7
	T_{h1}	-335.4
	T_{f1}	-323.0
H_2	*Horizontal above T_1*	-38.9
	Horizontal above T_3	-12.9
CCO	F, T_3	-609.7
	T_{h1}, B_3, B_1	-554.6
CCH	T_{f1}, B_1	-659.8
	F, $T_{2\text{-}3}$	-635.1
	T_{h2}, T_{f2}	-604.7
	T_{f2}, T_{h2}	-594.2
	T_{h1}, T_{f2}	-575.3
	T_{f2}, T_{h1}	-572.7
CCH_3	F	-580.0

CH_2CH_2	B_1, T_1	-119.5
CH_2CH_3	B_1	-26.0

Stable Geometry Figures

Ni(111)

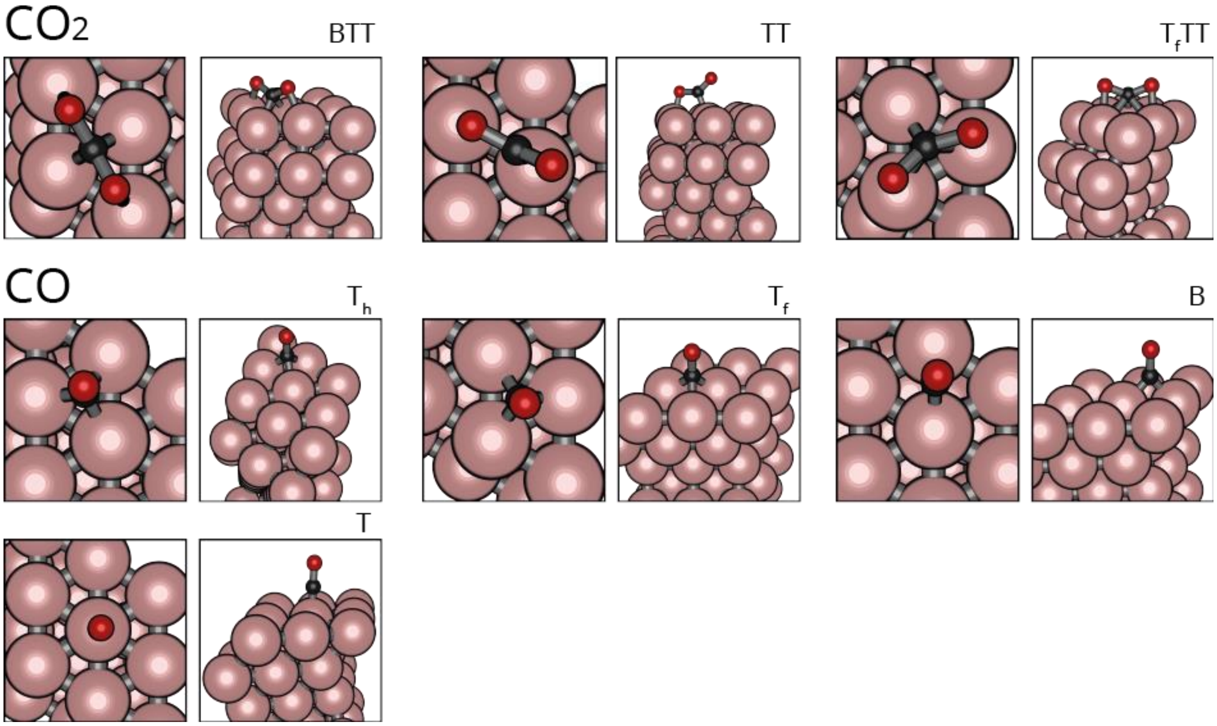

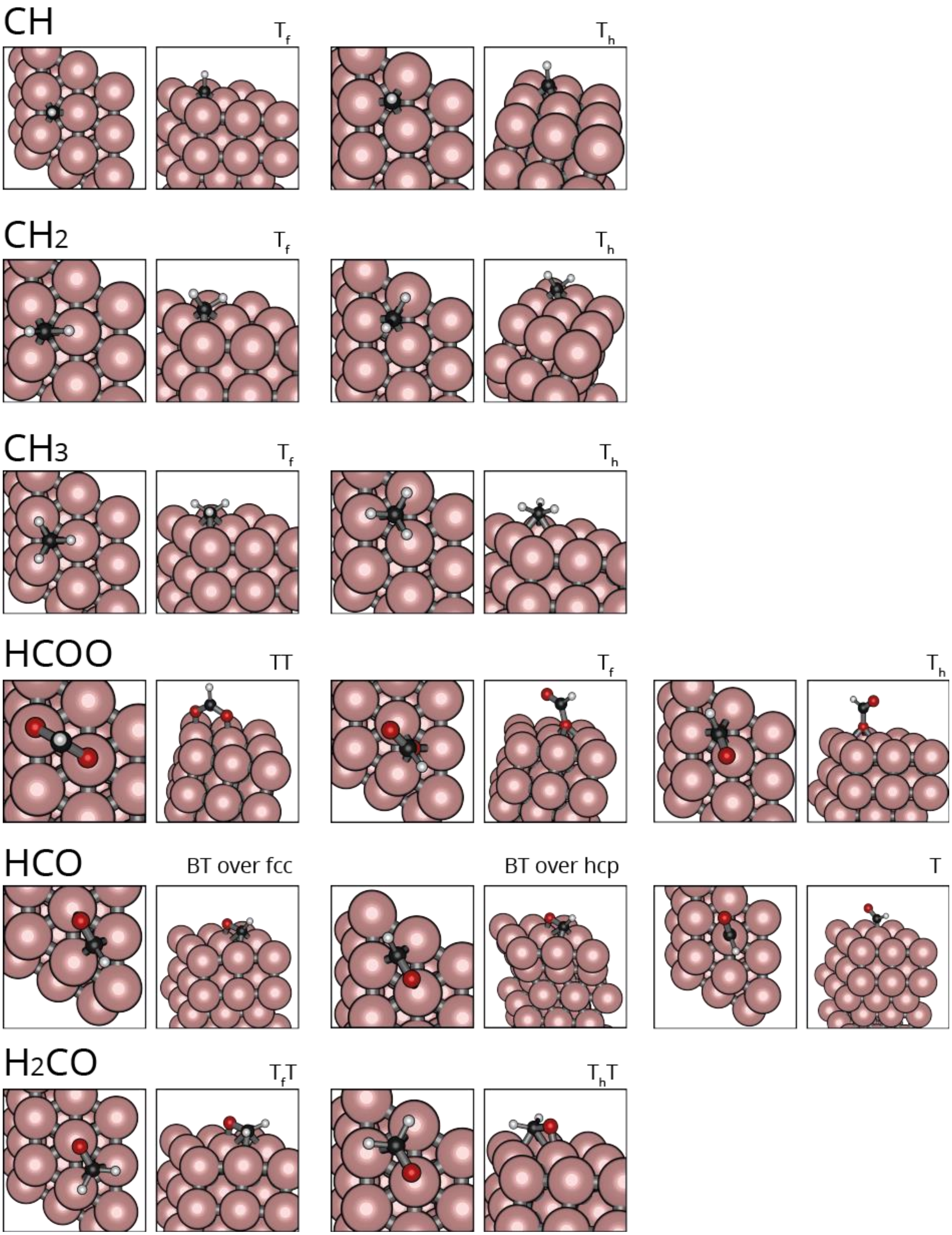
CH
T_f
T_h
CH_2
T_f
T_h
CH_3
T_f
T_h
HCOO
TT
T_f
T_h
HCO
BT over fcc
BT over hcp
T
H_2CO
T_fT
T_hT

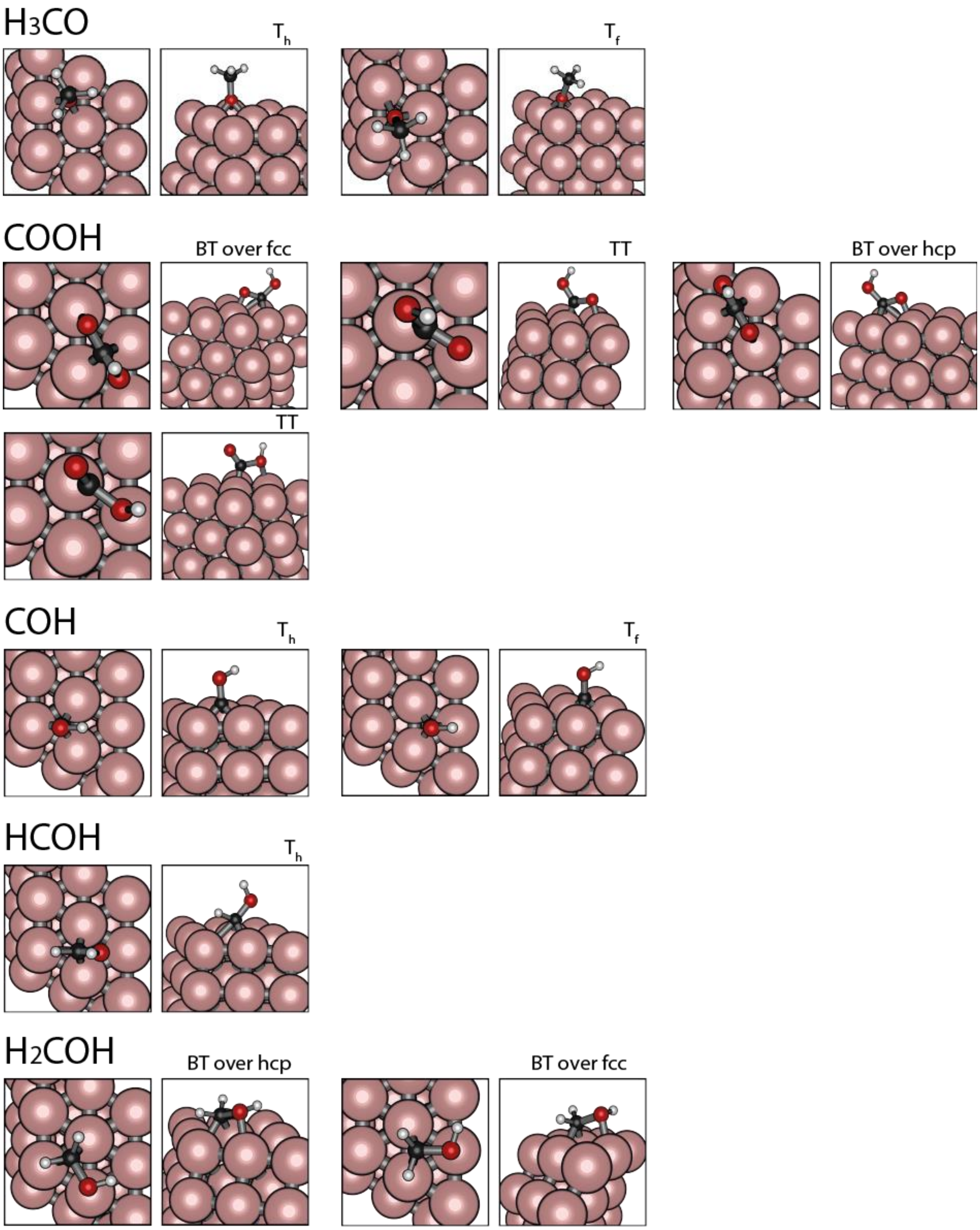
H3CO
Th
Tf
COOH
BT over fcc
TT
BT over hcp
TT
COH
Th
Tf
HCOH
Th
H2COH
BT over hcp
BT over fcc

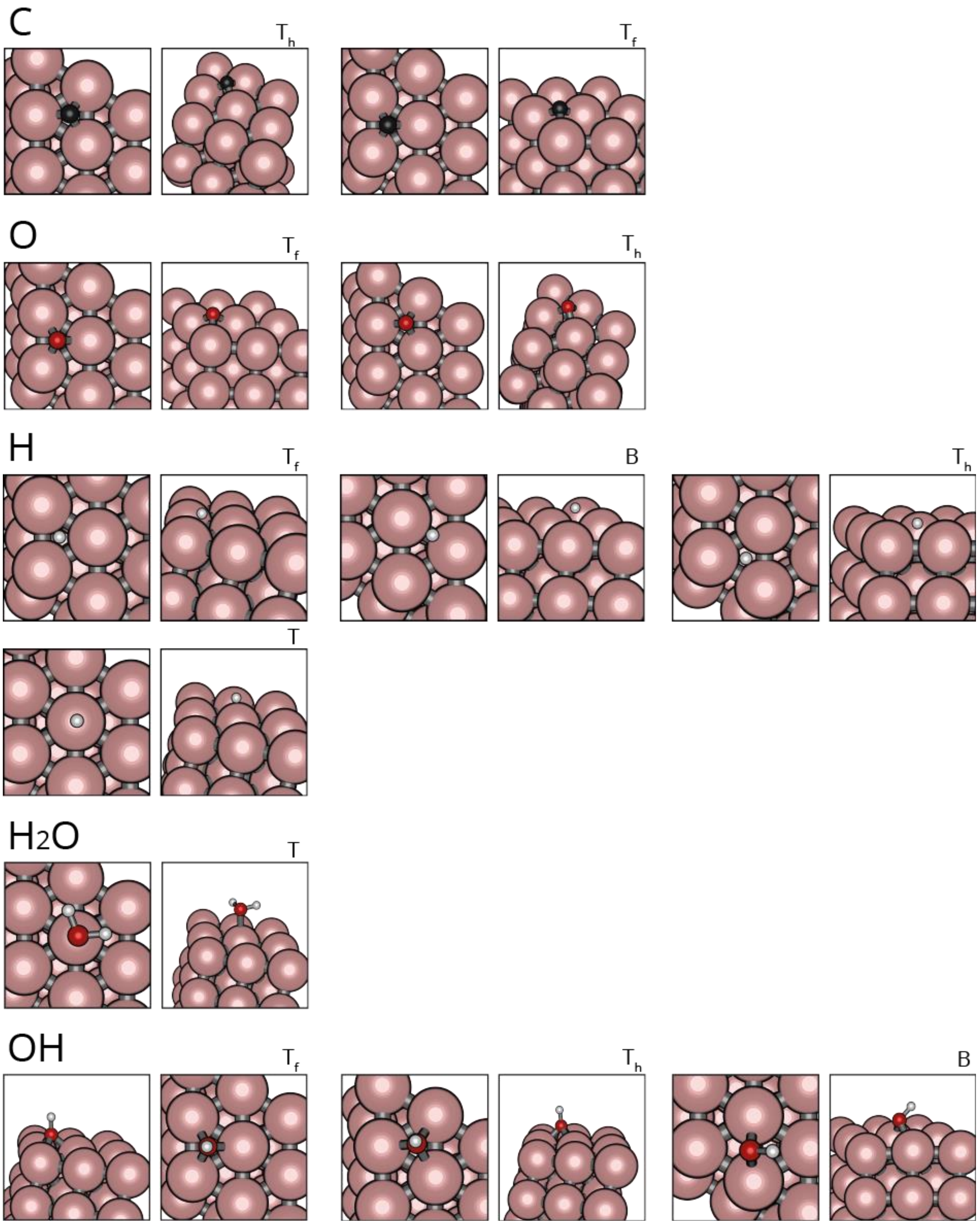
C
T_h
T_f
O
T_f
T_h
H
T_f
B
T_h
T
H_2O
T
OH
T_f
T_h
B

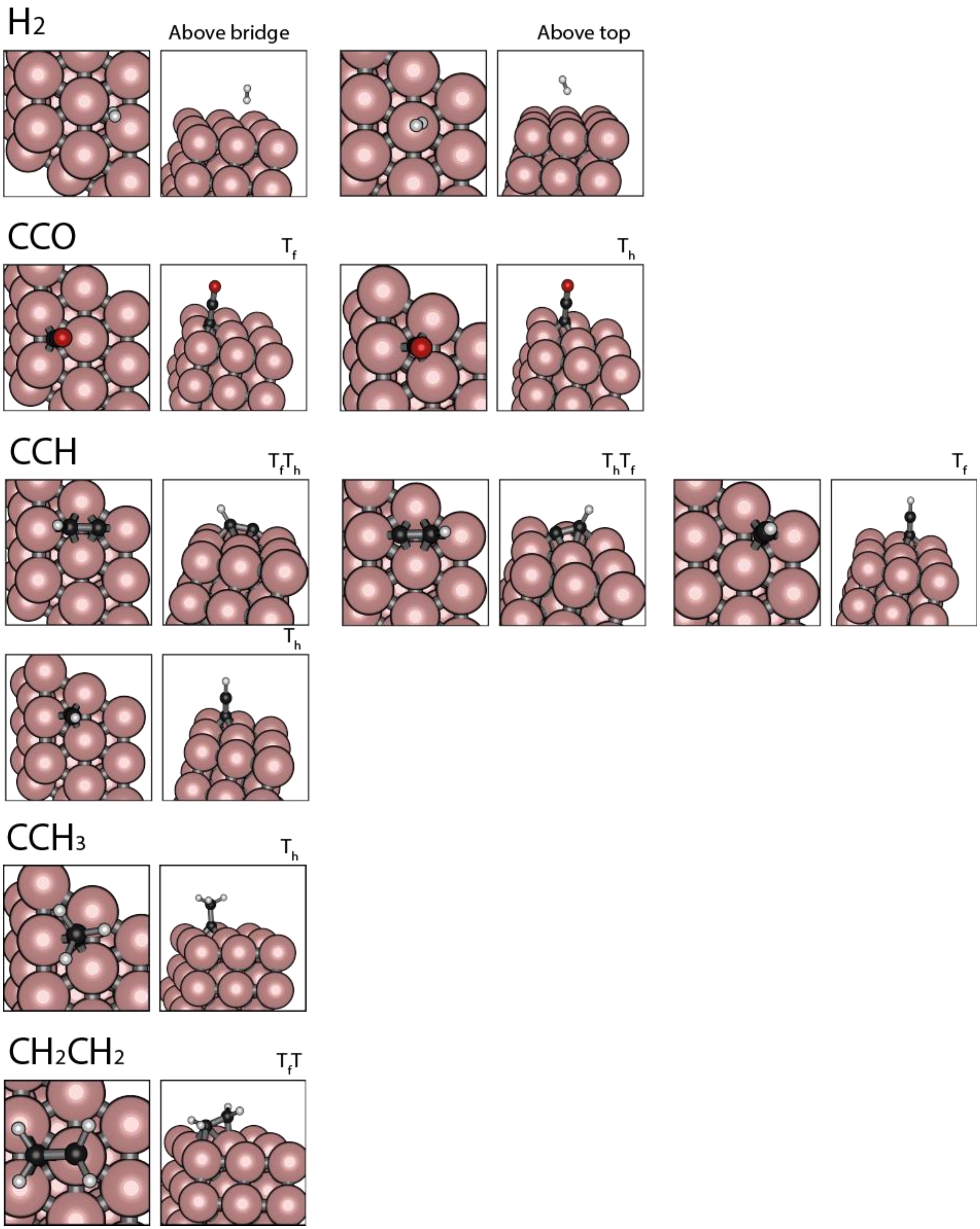
H2
Above bridge
Above top
CCO
Tf
Th
CCH
TfTh
ThTf
Tf
Th
CCH3
Th
CH2CH2
TfT

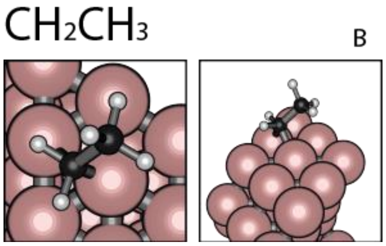

Supplementary Figure 14. Top and side view of stable geometries on Ni(111) calculated for each adsorbate of the carbide, formate and alcohol pathway. The stable geometries are ordered from left to right in descending stability, as can be seen in Supplementary Table 4.

Ni(100)

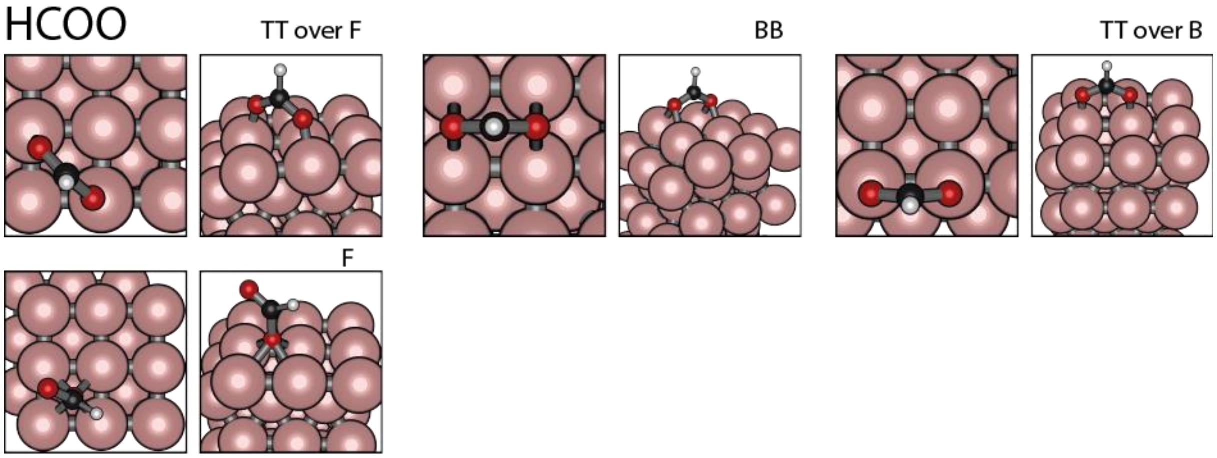

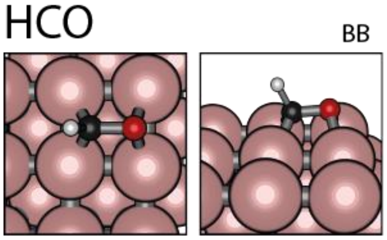

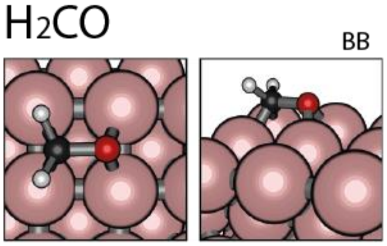

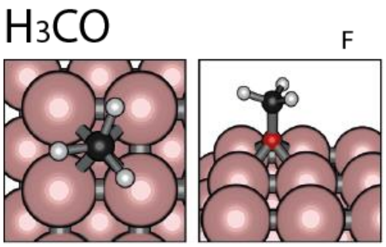

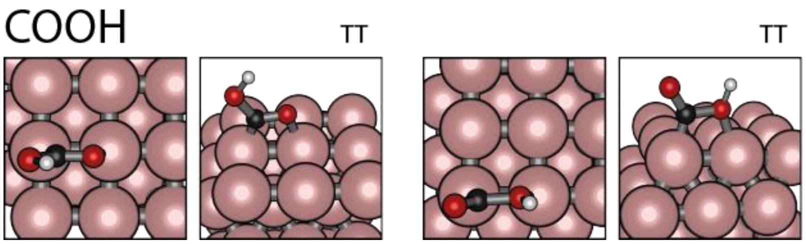

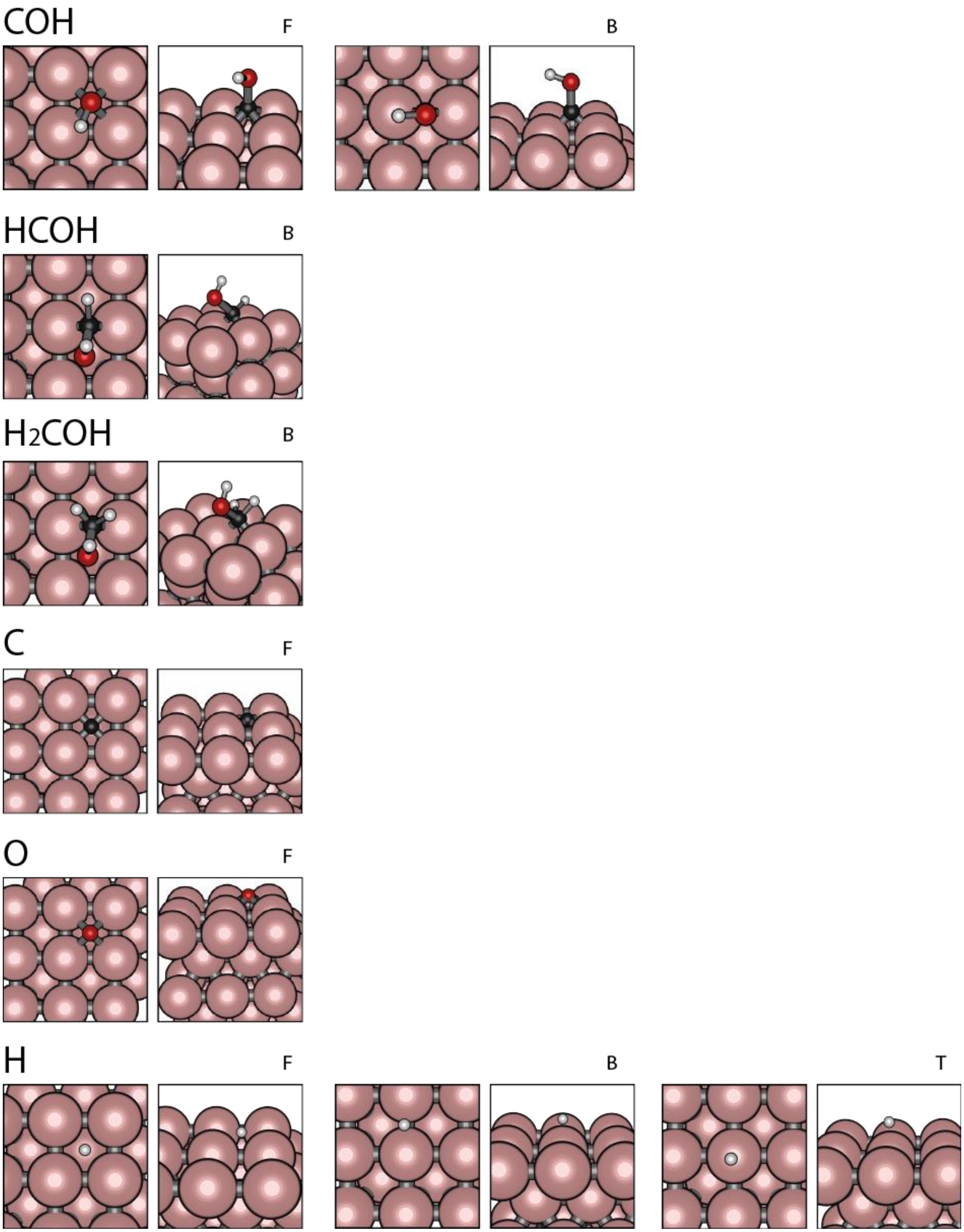
COH
F
B
HCOH
B
H2COH
B
C
F
O
F
H
F
B
T

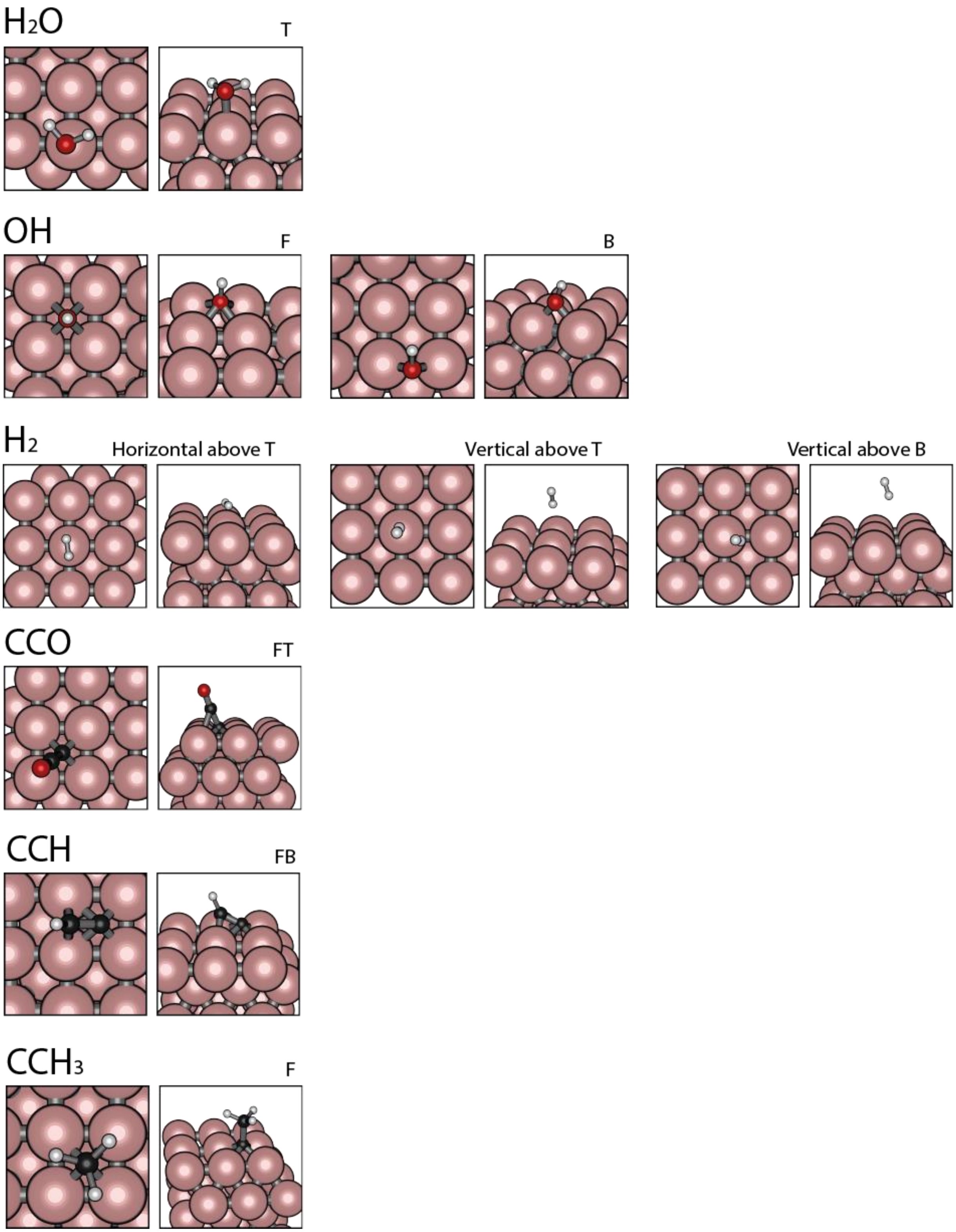
H2O
T
OH
F
B
H2
Horizontal above T
Vertical above T
Vertical above B
CCO
FT
CCH
FB
CCH3
F

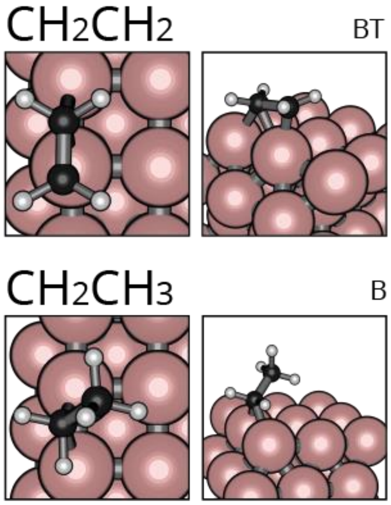
CH2CH2
BT
CH2CH3
B

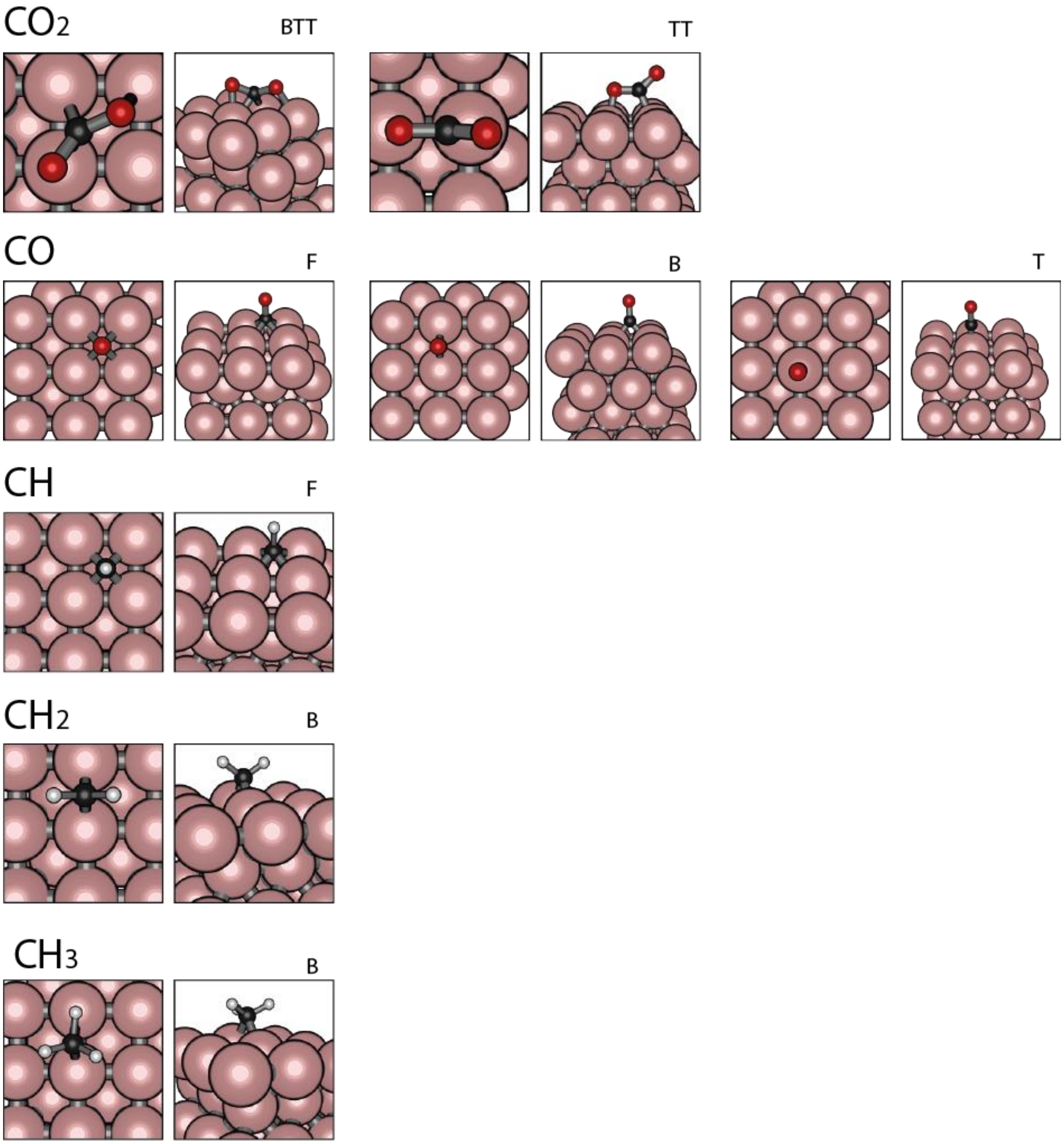

Supplementary Figure 15. Stable geometries on Ni(100) calculated for each adsorbate of the carbide, formate and alcohol pathway. The stable geometries are ordered from left to right in descending stability, as can be seen in Supplementary Table 4.

<u>Ni(110)</u>

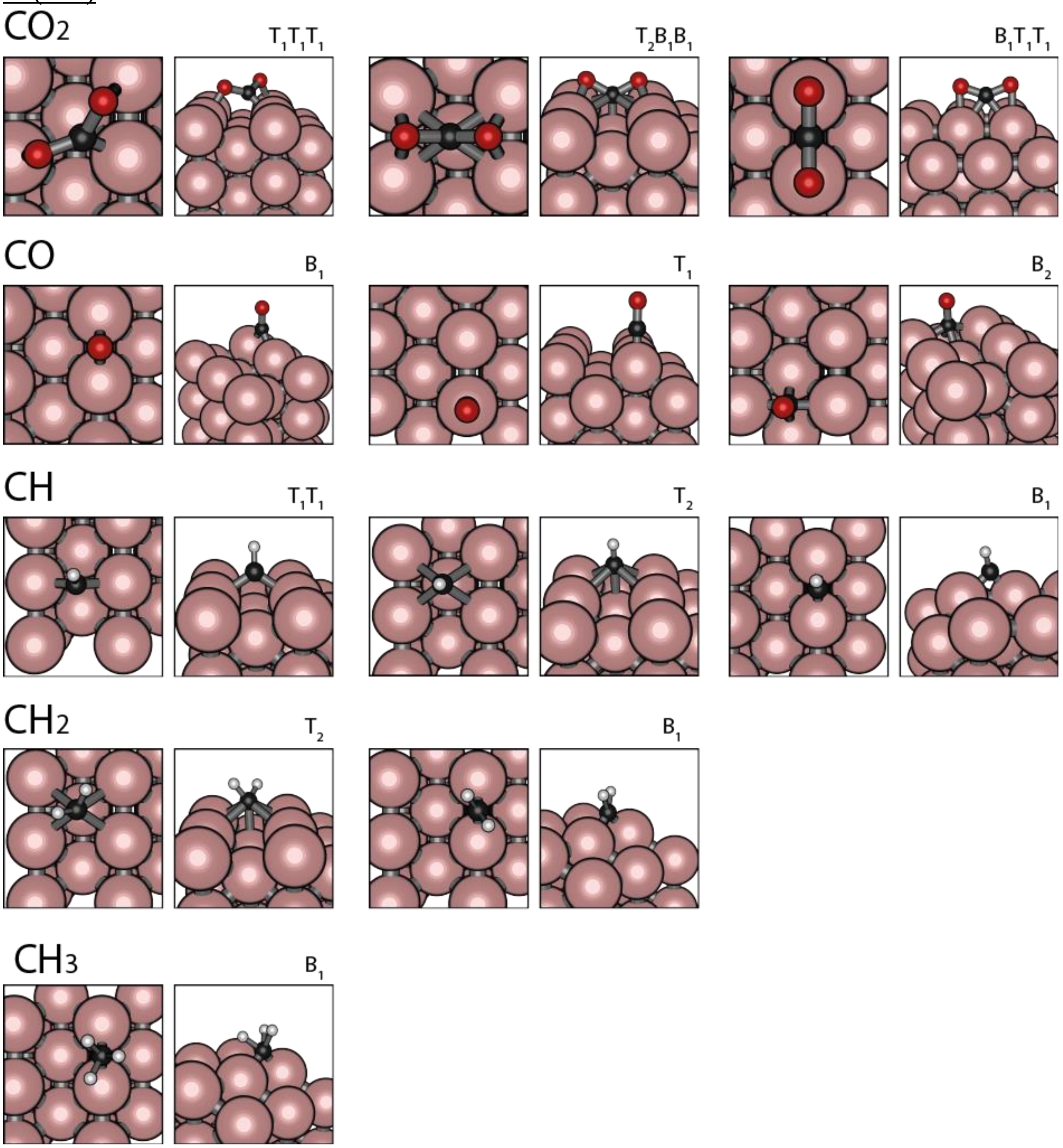
CO_2
$T_1T_1T_1$
$T_2B_1B_1$
$B_1T_1T_1$
CO
B_1
T_1
B_2
CH
T_1T_1
T_2
B_1
CH_2
T_2
B_1
CH_3
B_1

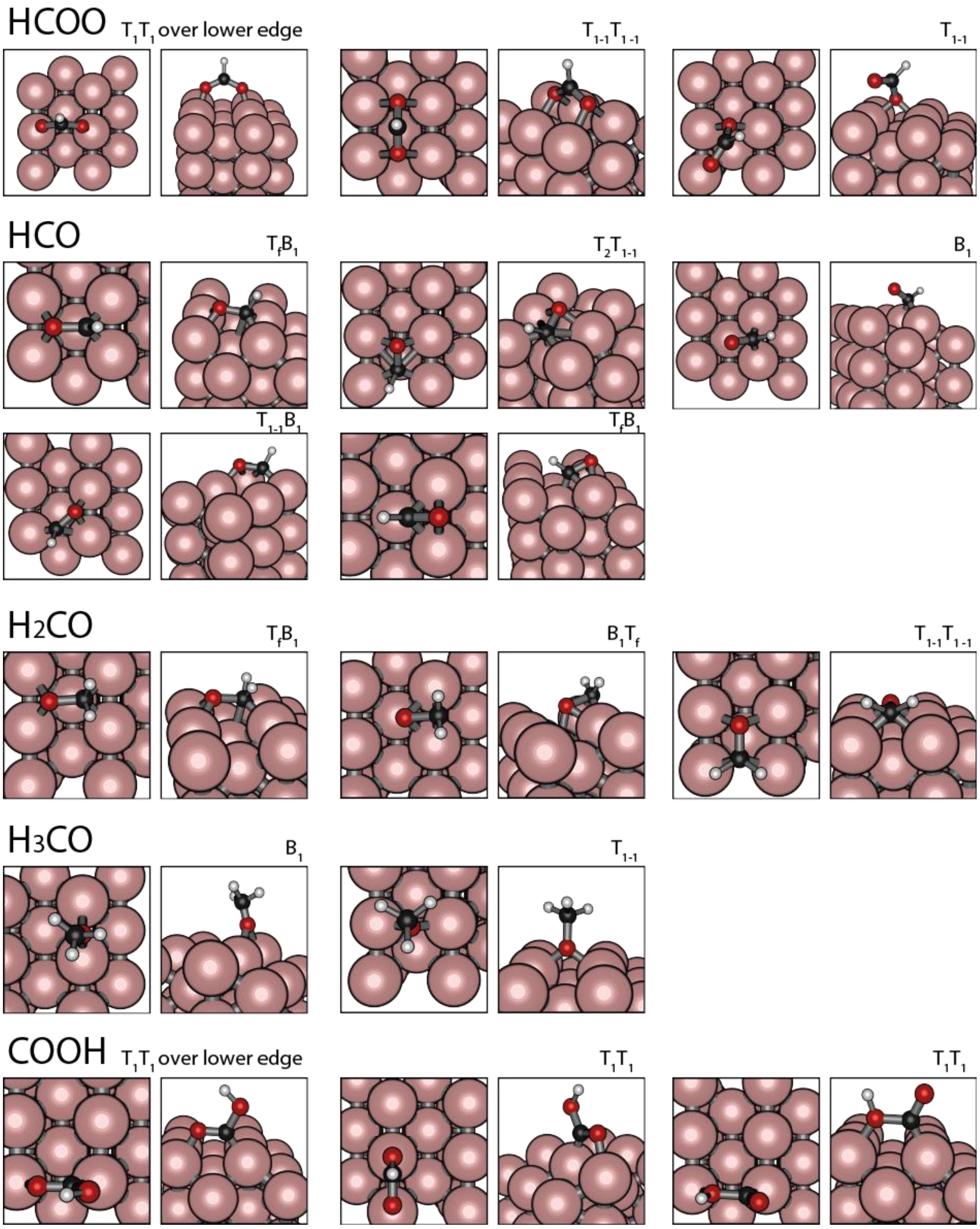
HCOO
T_1T_1 over lower edge
$T_{1\text{-}1}T_{1\text{-}1}$
$T_{1\text{-}1}$
HCO
T_fB_1
$T_2T_{1\text{-}1}$
B_1
$T_{1\text{-}1}B_1$
T_fB_1
H_2CO
T_fB_1
B_1T_f
$T_{1\text{-}1}T_{1\text{-}1}$
H_3CO
B_1
$T_{1\text{-}1}$
COOH
T_1T_1 over lower edge
T_1T_1
T_1T_1

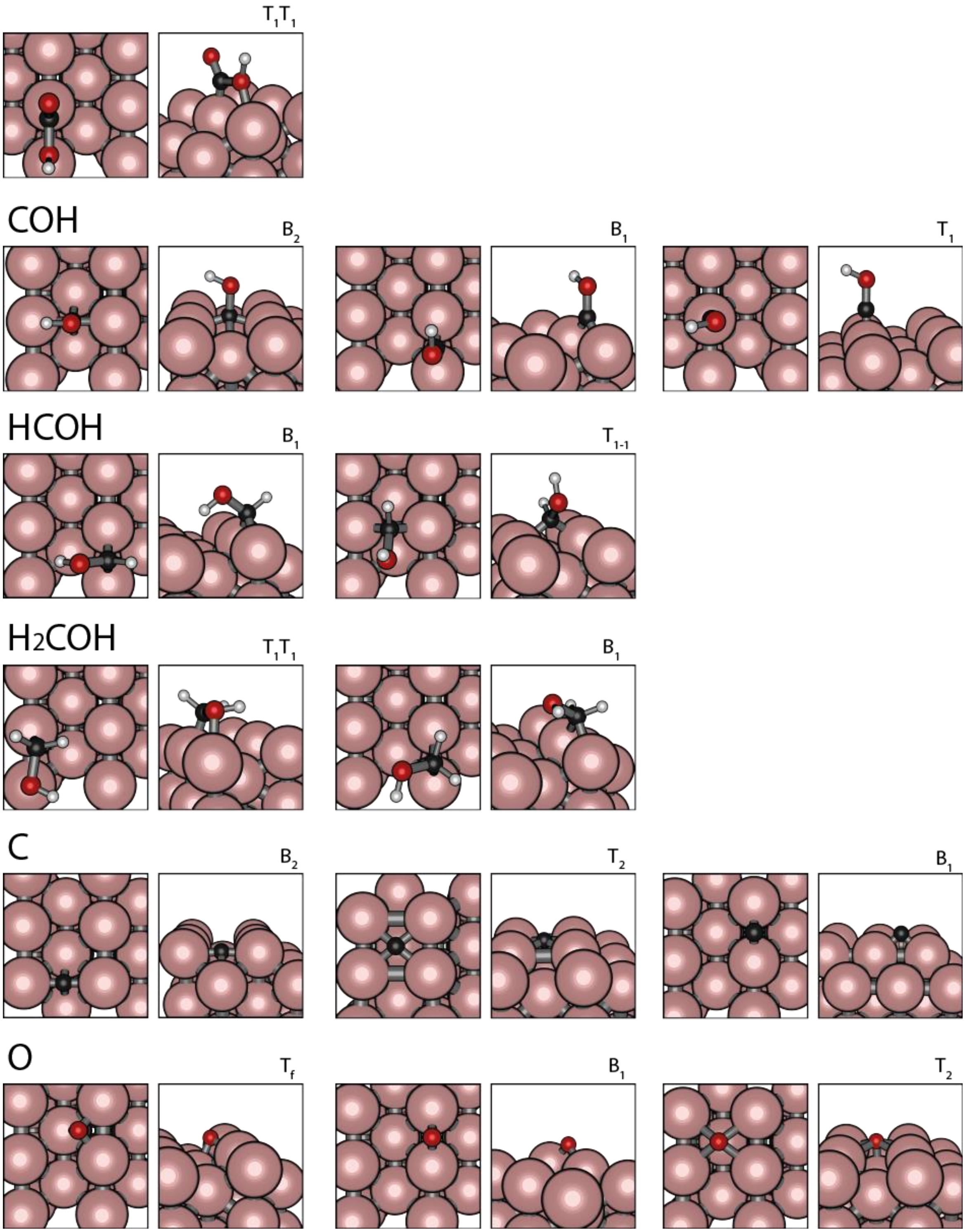
T_1T_1
COH
B_2
B_1
T_1
HCOH
B_1
T_{1-1}
H_2COH
T_1T_1
B_1
C
B_2
T_2
B_1
O
T_f
B_1
T_2

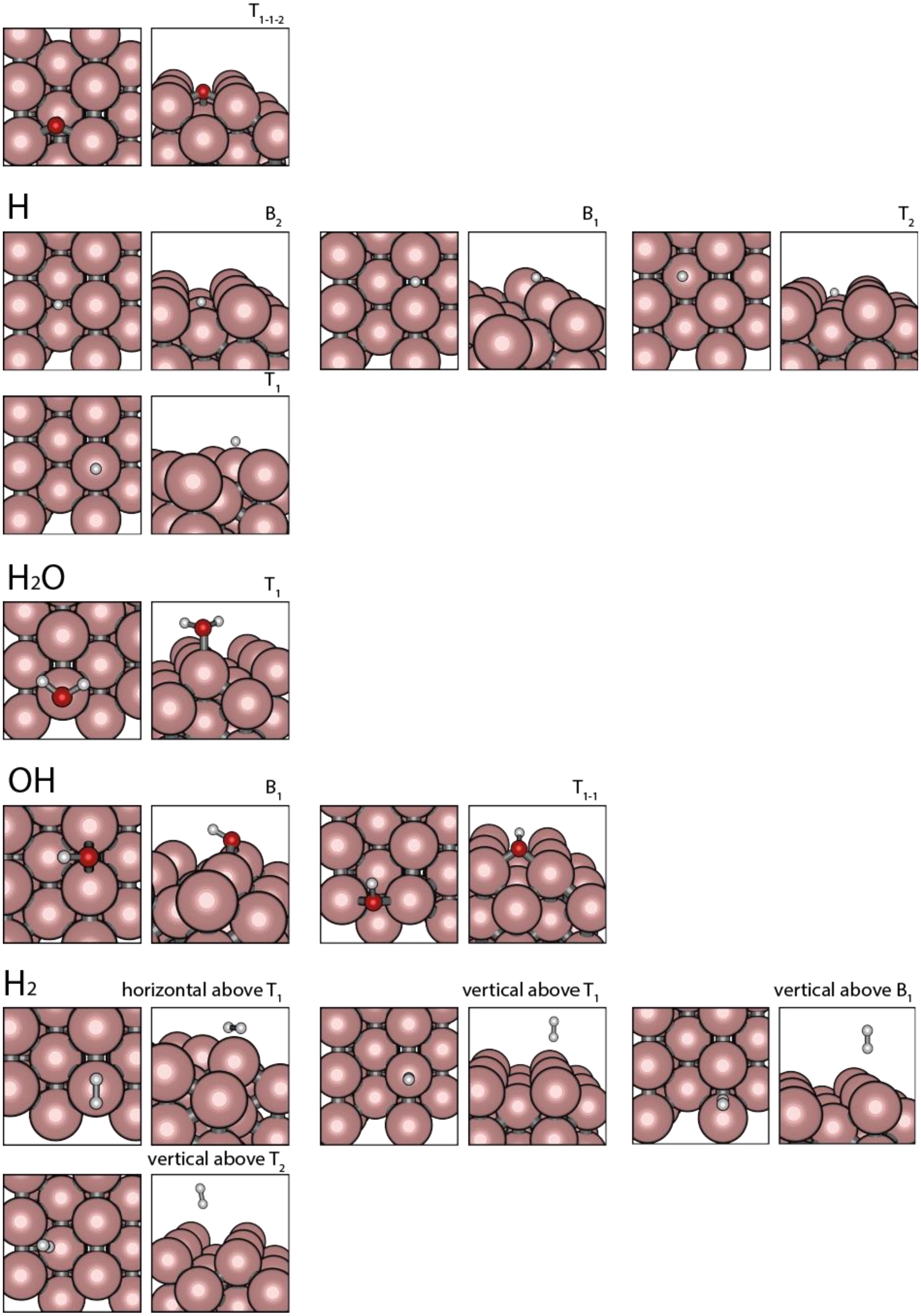

Supplementary Figure 16. Stable geometries on Ni(110) calculated for each adsorbate of the carbide, formate and alcohol pathway. The stable geometries are ordered from left to right in descending stability, as can be seen in Supplementary Table 4.

Ni(211)

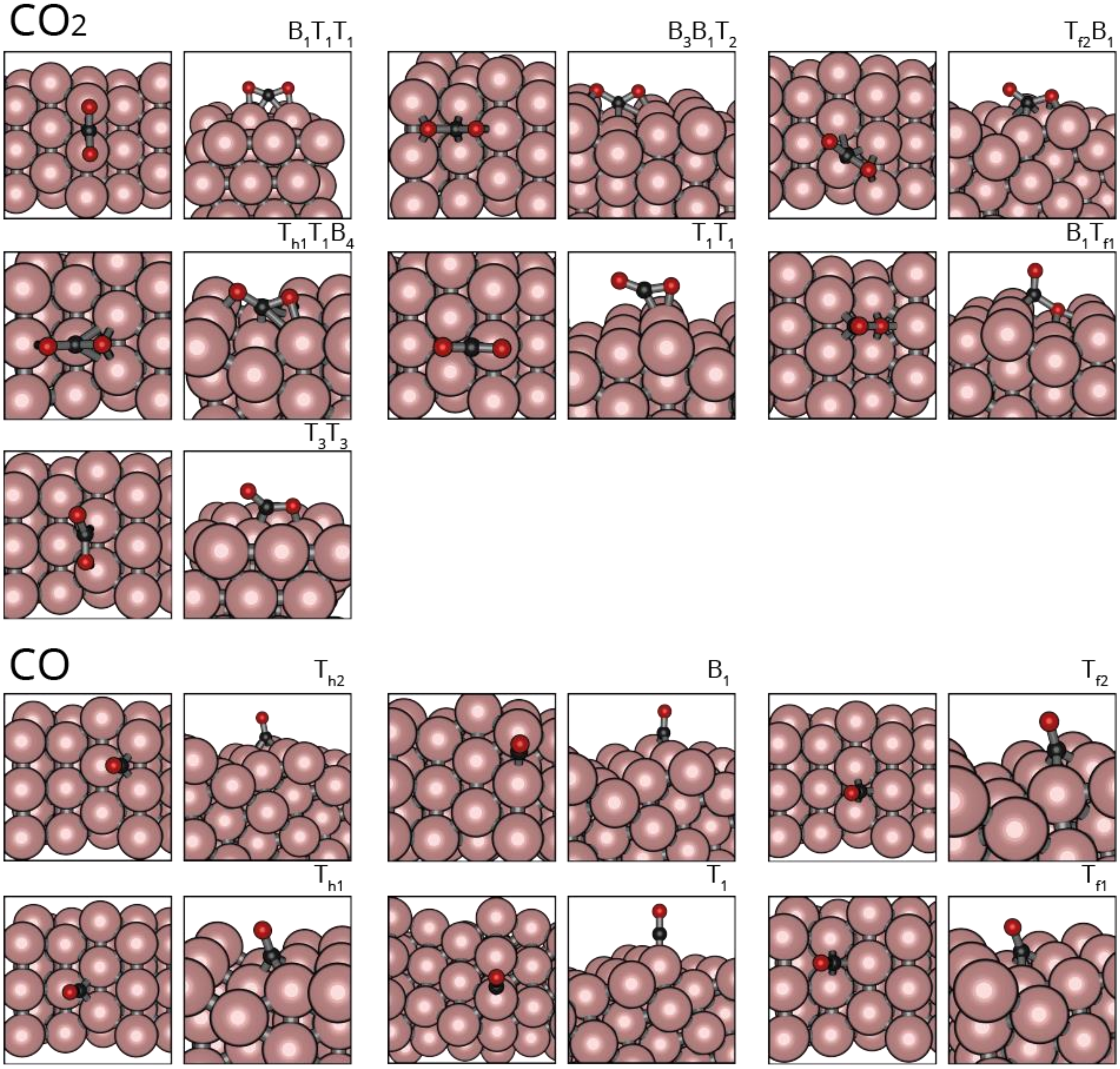

CO_2
$B_1T_1T_1$
$B_3B_1T_2$
$T_{f2}B_1$
$T_{h1}T_1B_4$
T_1T_1
B_1T_{f1}
T_3T_3
CO
T_{h2}
B_1
T_{f2}
T_{h1}
T_1
T_{f1}

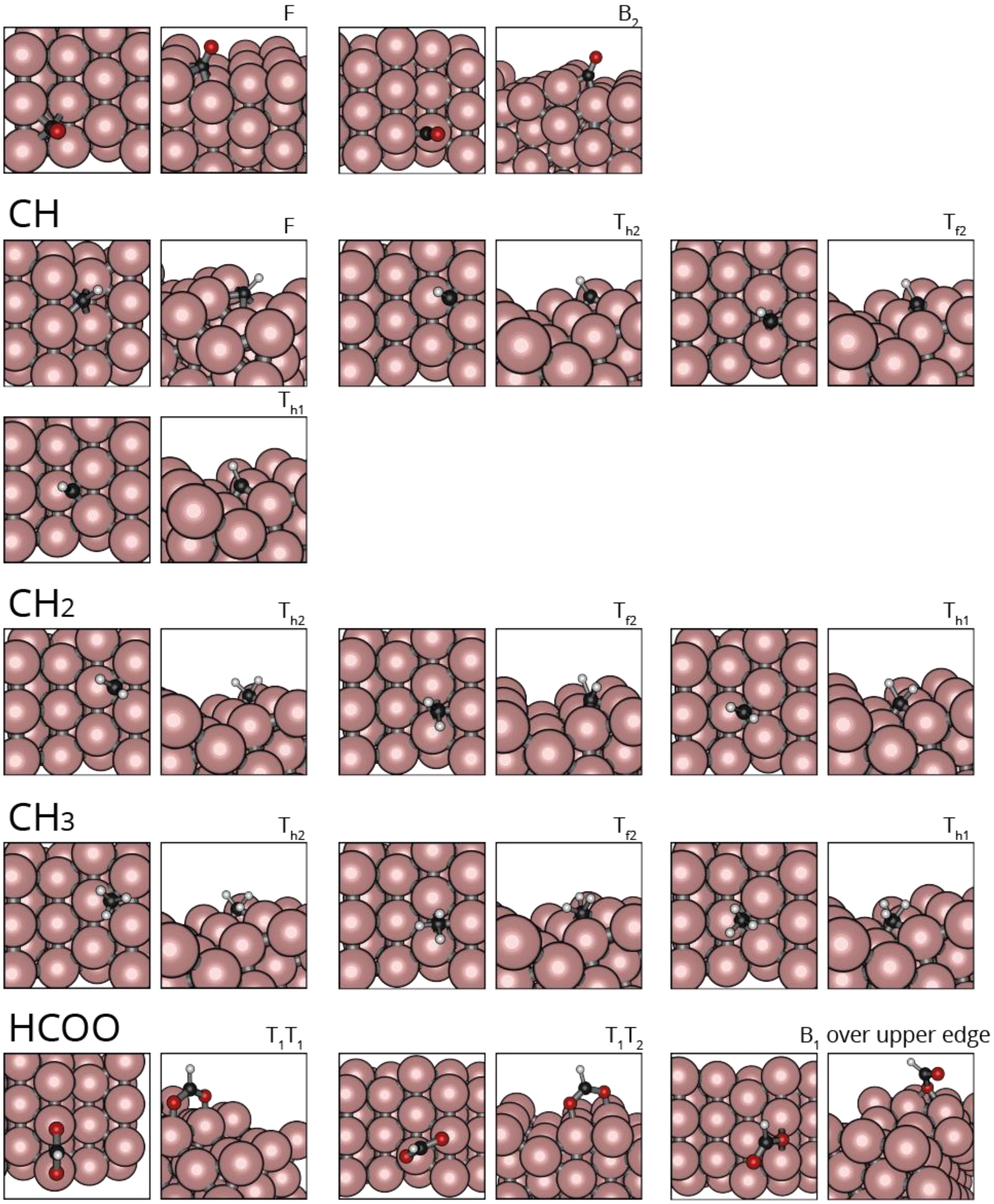
F
B_2
CH
F
T_{h2}
T_{f2}
T_{h1}
CH_2
T_{h2}
T_{f2}
T_{h1}
CH_3
T_{h2}
T_{f2}
T_{h1}
HCOO
T_1T_1
T_1T_2
B_1 over upper edge

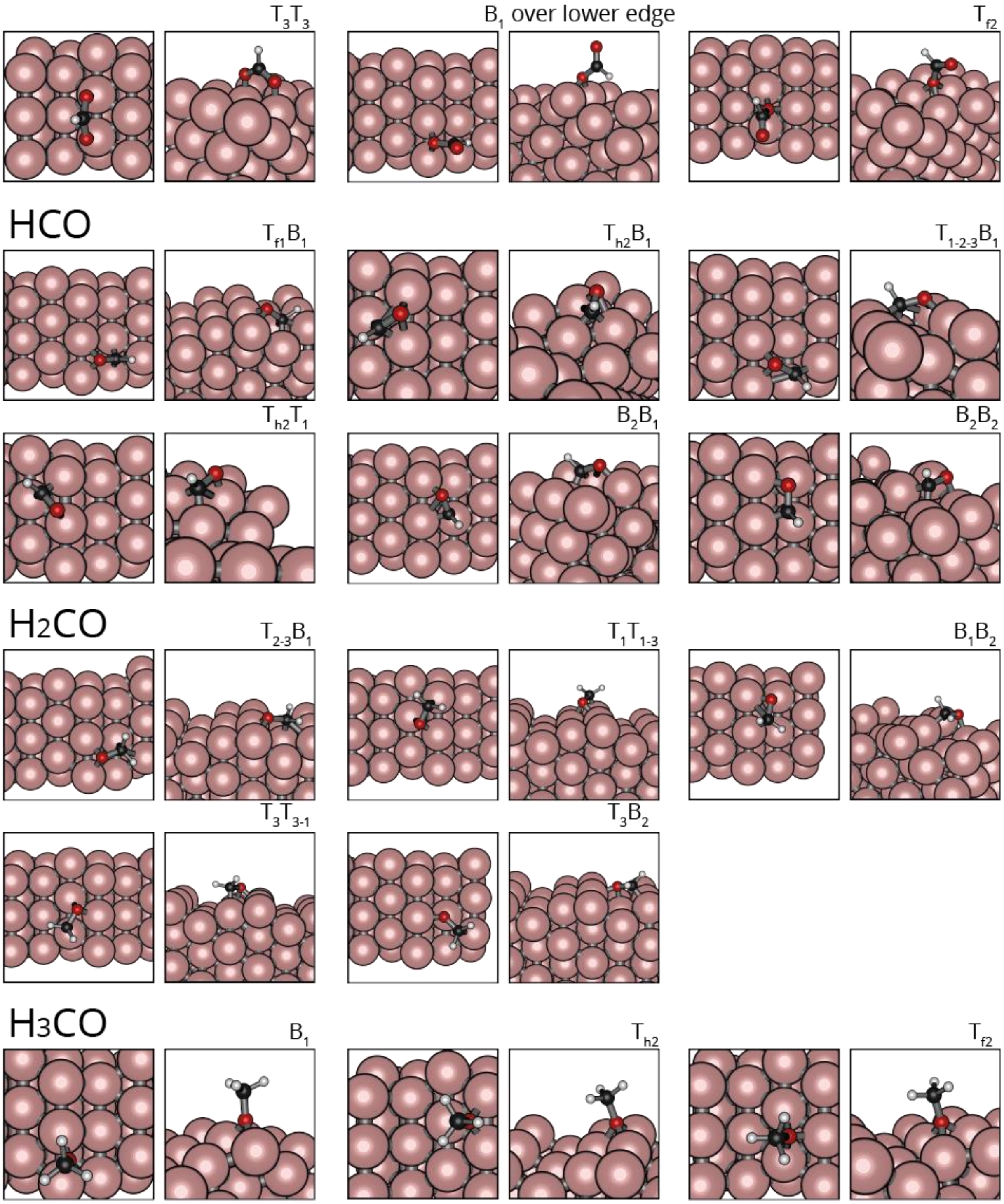
T_3T_3
B_1 over lower edge
T_{f2}
HCO
$T_{f1}B_1$
$T_{h2}B_1$
$T_{1\text{-}2\text{-}3}B_1$
$T_{h2}T_1$
B_2B_1
B_2B_2
H_2CO
$T_{2\text{-}3}B_1$
$T_1T_{1\text{-}3}$
B_1B_2
$T_3T_{3\text{-}1}$
T_3B_2
H_3CO
B_1
T_{h2}
T_{f2}

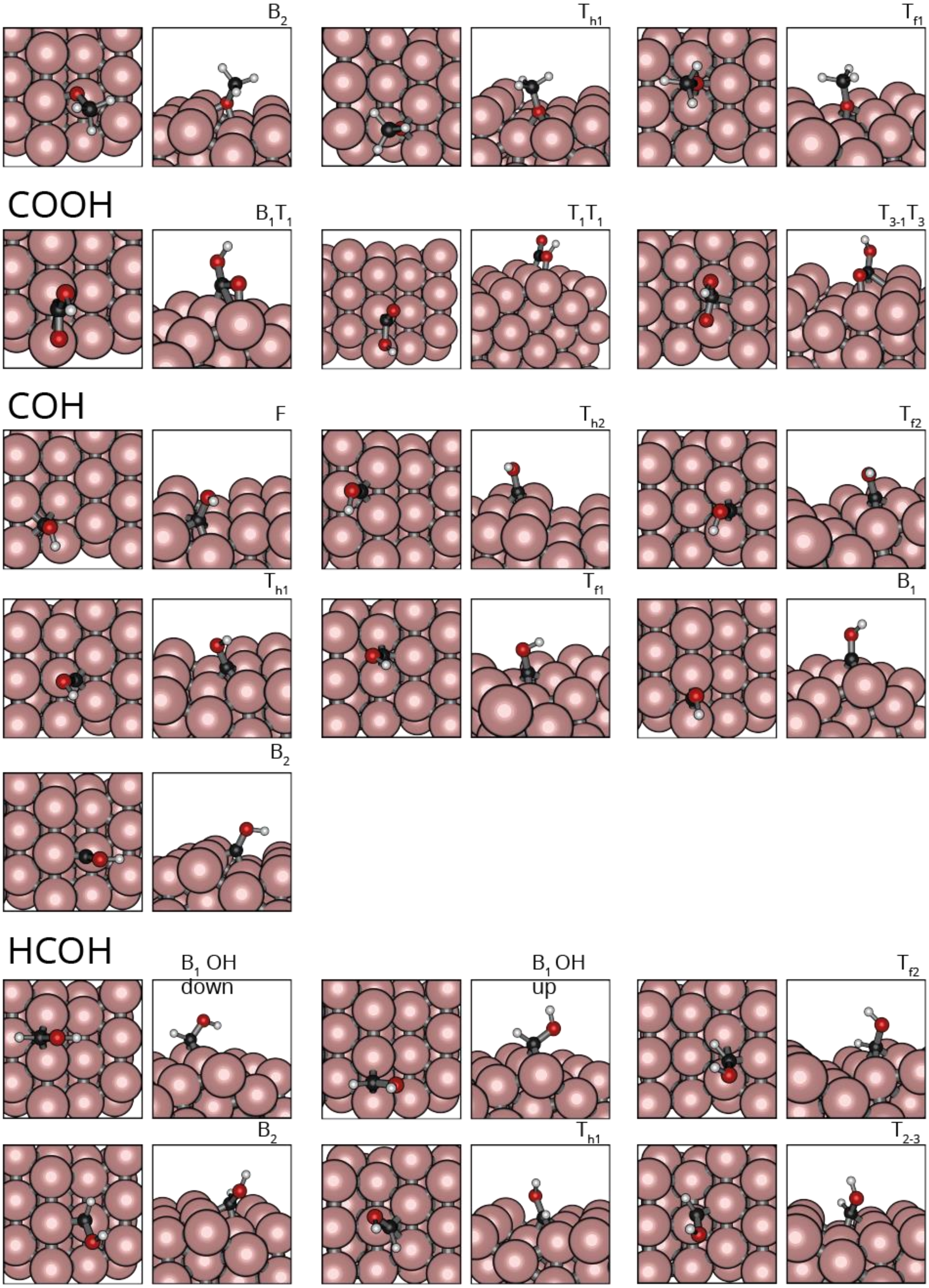

B_2
T_{h1}
T_{f1}
COOH
B_1T_1
T_1T_1
$T_{3-1}T_3$
COH
F
T_{h2}
T_{f2}
T_{h1}
T_{f1}
B_1
B_2
HCOH
B_1 OH down
B_1 OH up
T_{f2}
B_2
T_{h1}
T_{2-3}

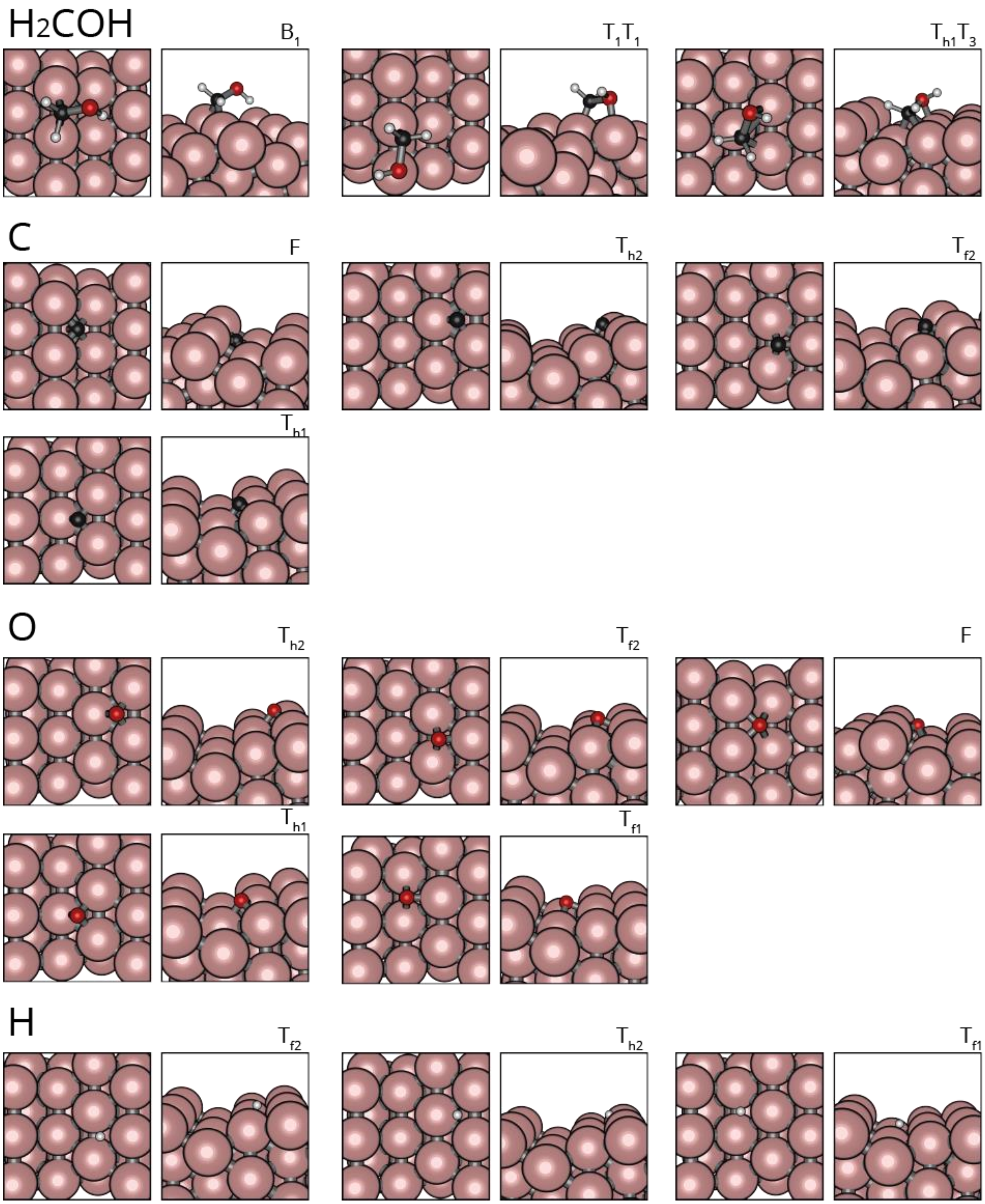

H_2COH
B_1
T_1T_1
$T_{h1}T_3$
C
F
T_{h2}
T_{f2}
T_{h1}
O
T_{h2}
T_{f2}
F
T_{h1}
T_{f1}
H
T_{f2}
T_{h2}
T_{f1}

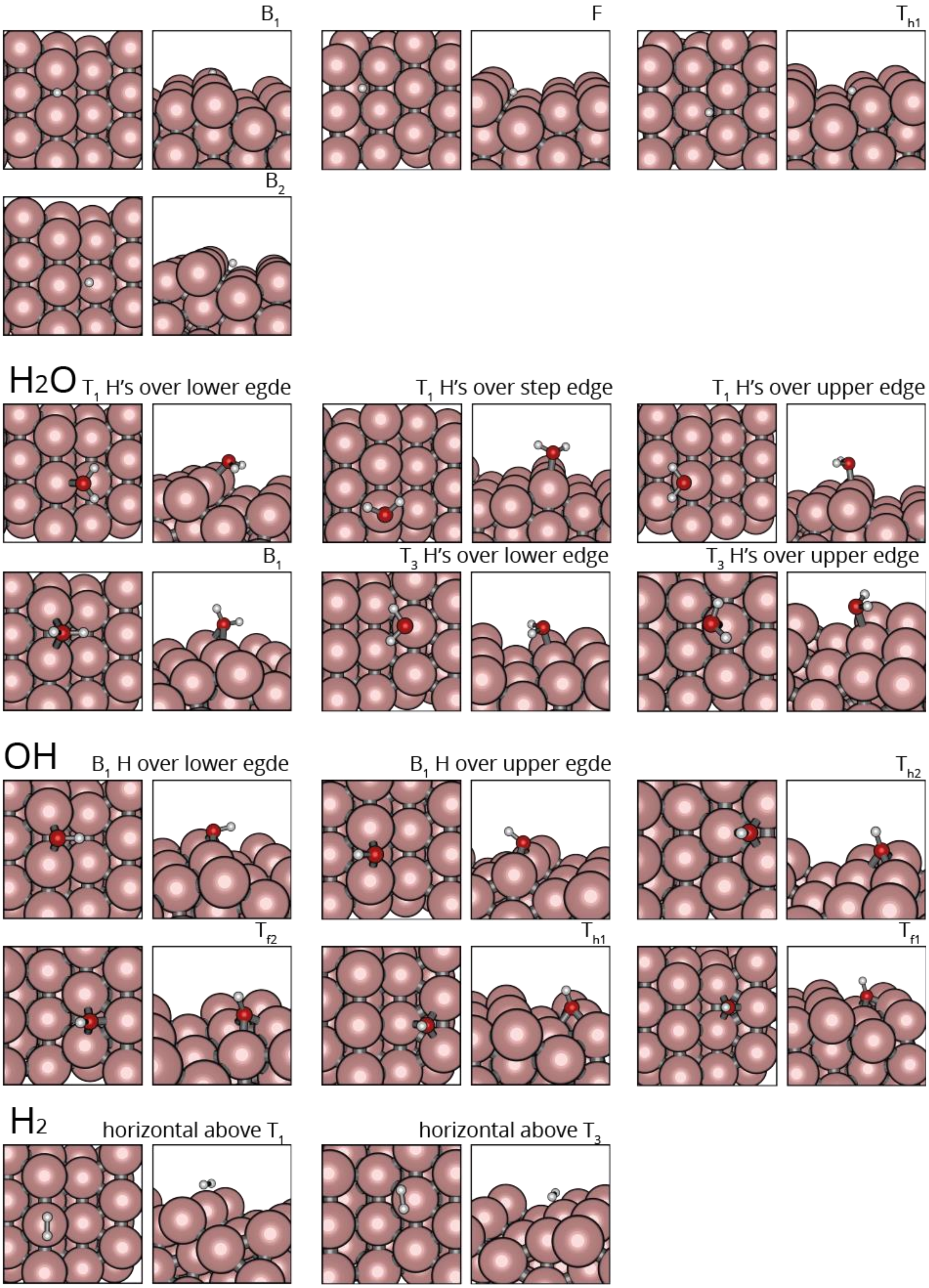
B_1
F
T_{h1}
B_2
H_2O
T_1 H's over lower egde
T_1 H's over step edge
T_1 H's over upper edge
B_1
T_3 H's over lower edge
T_3 H's over upper edge
OH
B_1 H over lower egde
B_1 H over upper egde
T_{h2}
T_{f2}
T_{h1}
T_{f1}
H_2
horizontal above T_1
horizontal above T_3

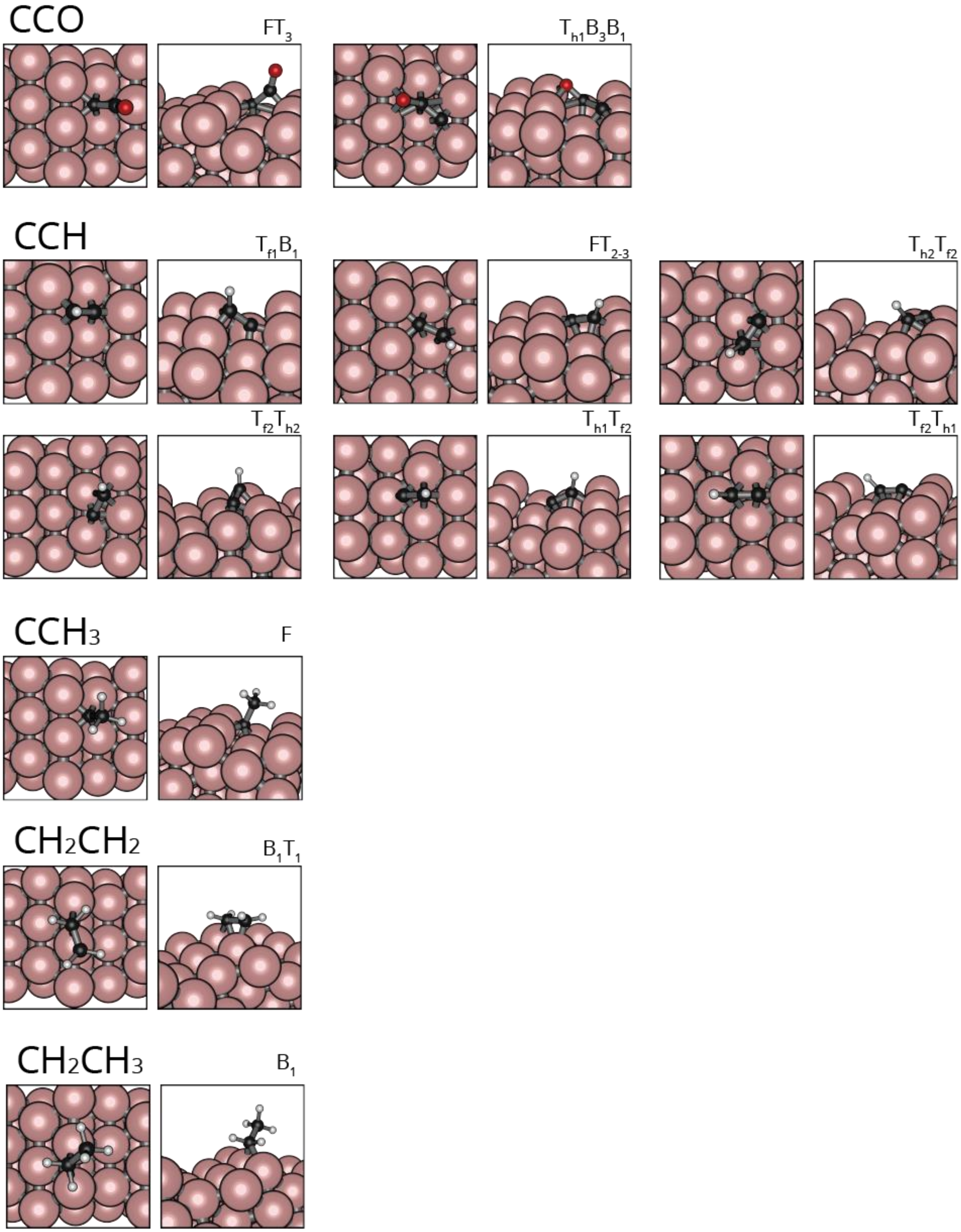

Supplementary Figure 17. Stable geometries on Ni(211) calculated for each adsorbate of the carbide, formate and alcohol pathway. The stable geometries are ordered from left to right in descending stability, as can be seen in Supplementary Table 4.

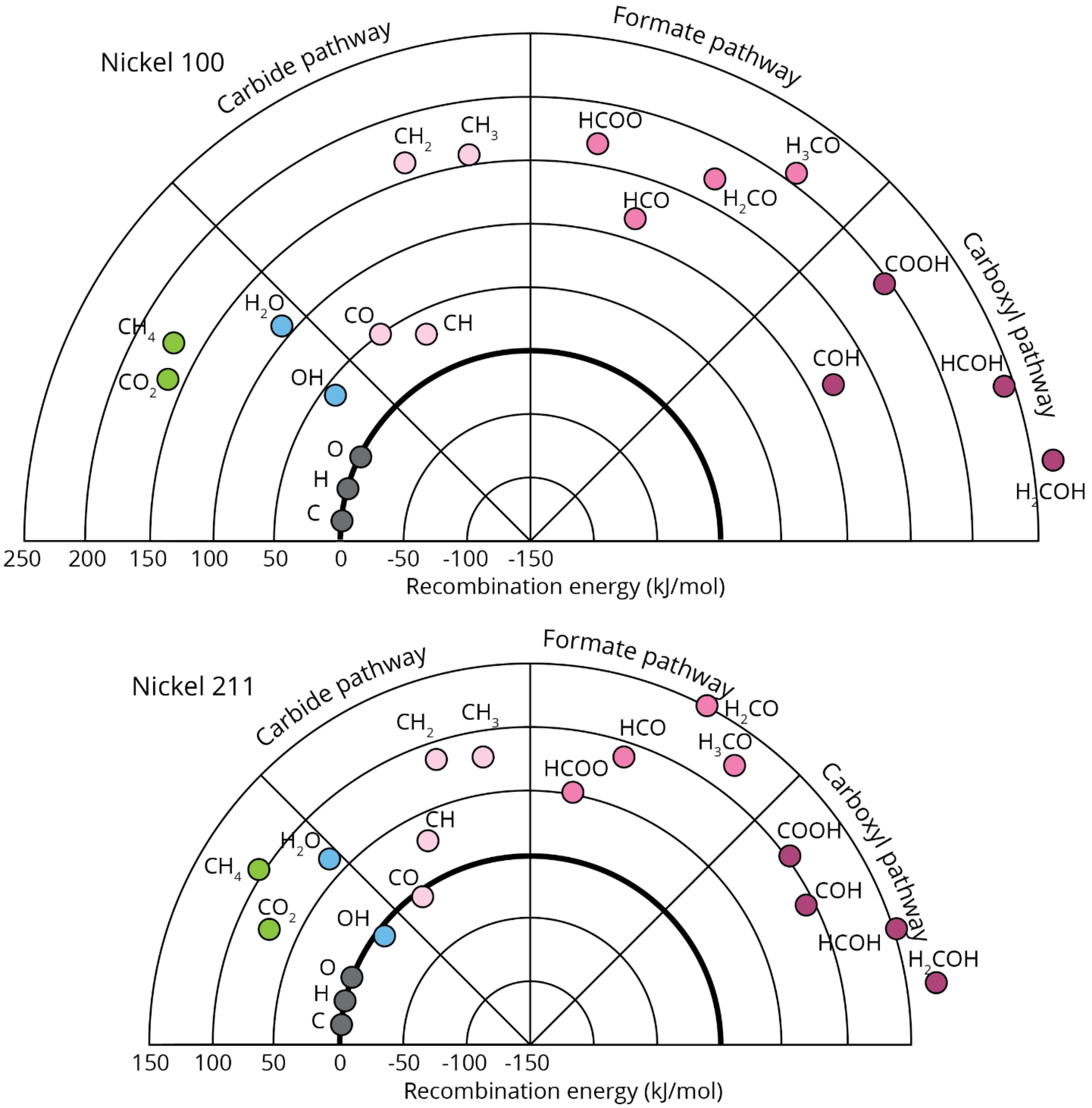

Supplementary Figure 18. Recombination energies of the carbide, formate and carboxyl pathways on a terrace Ni(100), and a stepped Ni(211) crystal facet. The left panel in each respective figure shows the C, H, and O adatoms from which each intermediate is recombined, as well as reaction intermediates OH and H_2O. C, H and O on Ni(100) are in a highly-stable F site (see Supplementary Figure 13) which leads to seemingly unfavorable recombination energies for this facet.

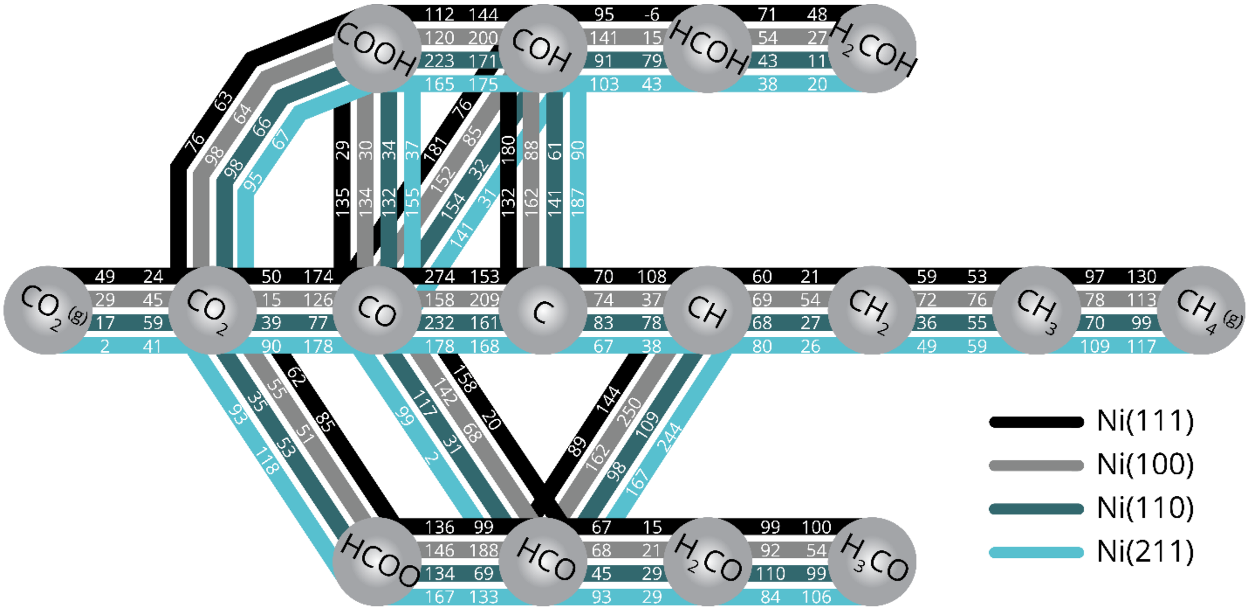

Supplementary Figure 19. Energy barriers overview for all calculated reaction steps in the different pathways and in-between pathways. In each segment, the values on the left denote the forward activation barrier (E_{aF}) over each Ni facet, while the values on the right denote the backward activation barrier (E_{aB}). Values are in kJ/mol.

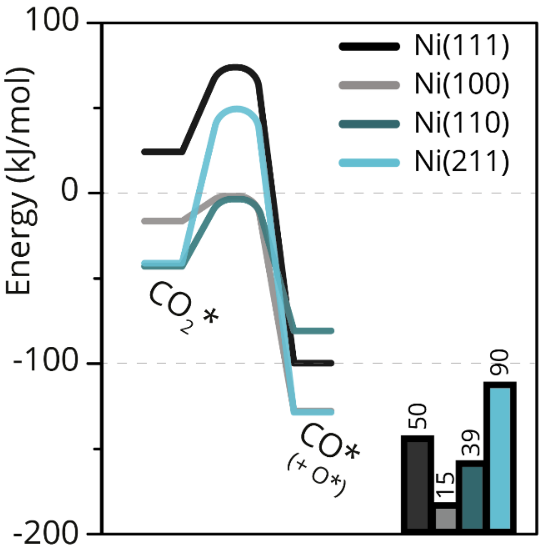

Supplementary Figure 20. Energy barriers for the direct CO_2 to CO dissociation on terrace facets Ni(111) and Ni(100), and stepped facets Ni(110) and Ni(211) as calculated by nudged elastic band.

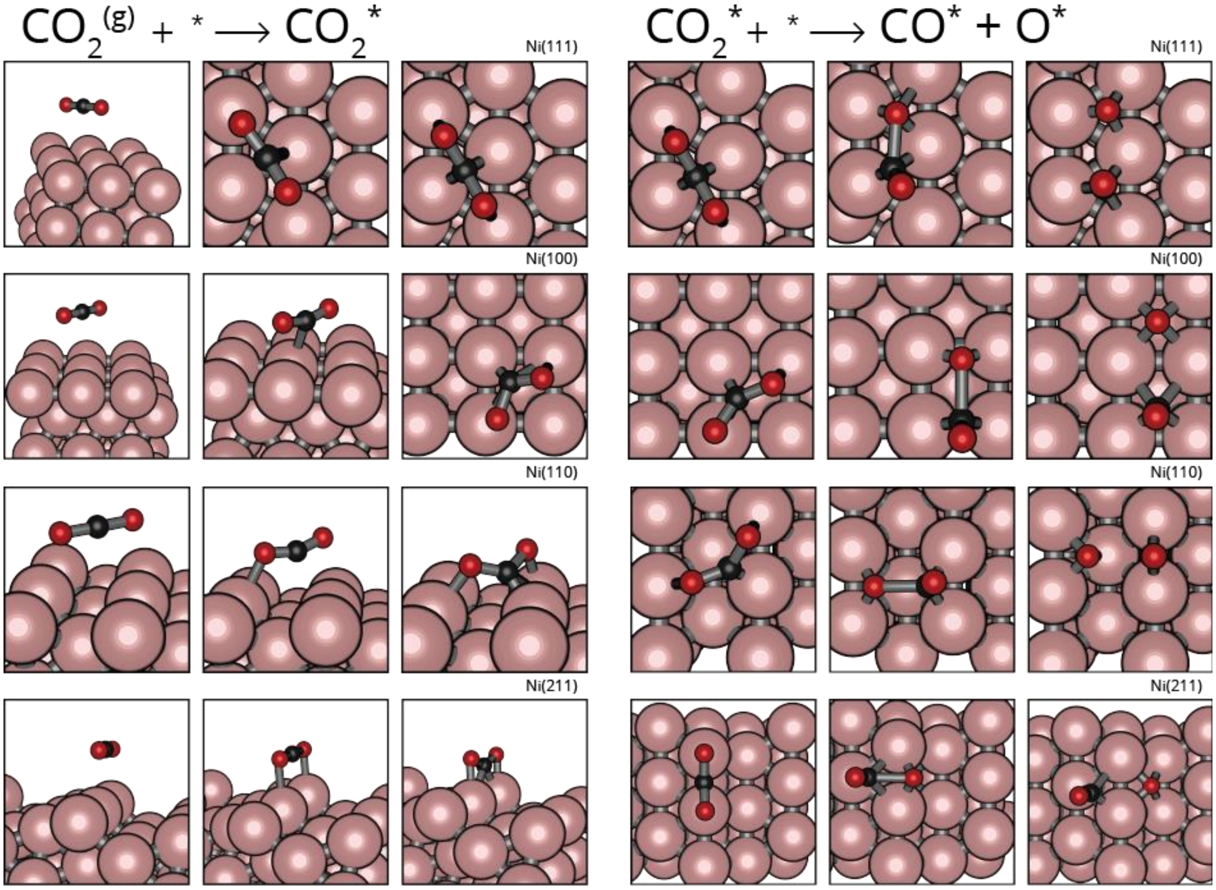
$CO_2^{(g)} + {}^* \longrightarrow CO_2^*$
$CO_2^* + {}^* \longrightarrow CO^* + O^*$
Ni(111)
Ni(100)
Ni(110)
Ni(211)
Ni(111)
Ni(100)
Ni(110)
Ni(211)

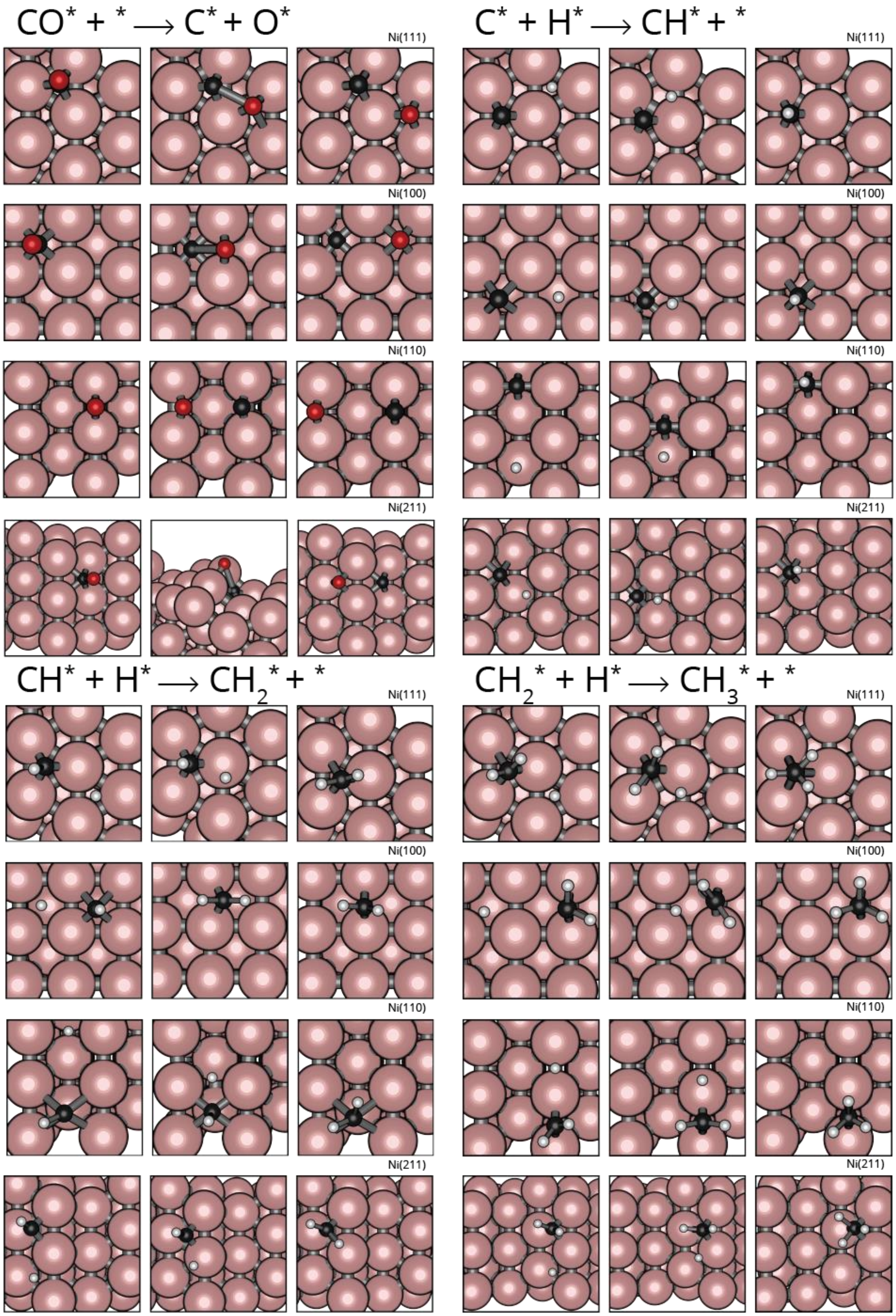
CO* + * ⟶ C* + O*
C* + H* ⟶ CH* + *
CH* + H* ⟶ CH2* + *
CH2* + H* ⟶ CH3* + *
Ni(111)
Ni(100)
Ni(110)
Ni(211)

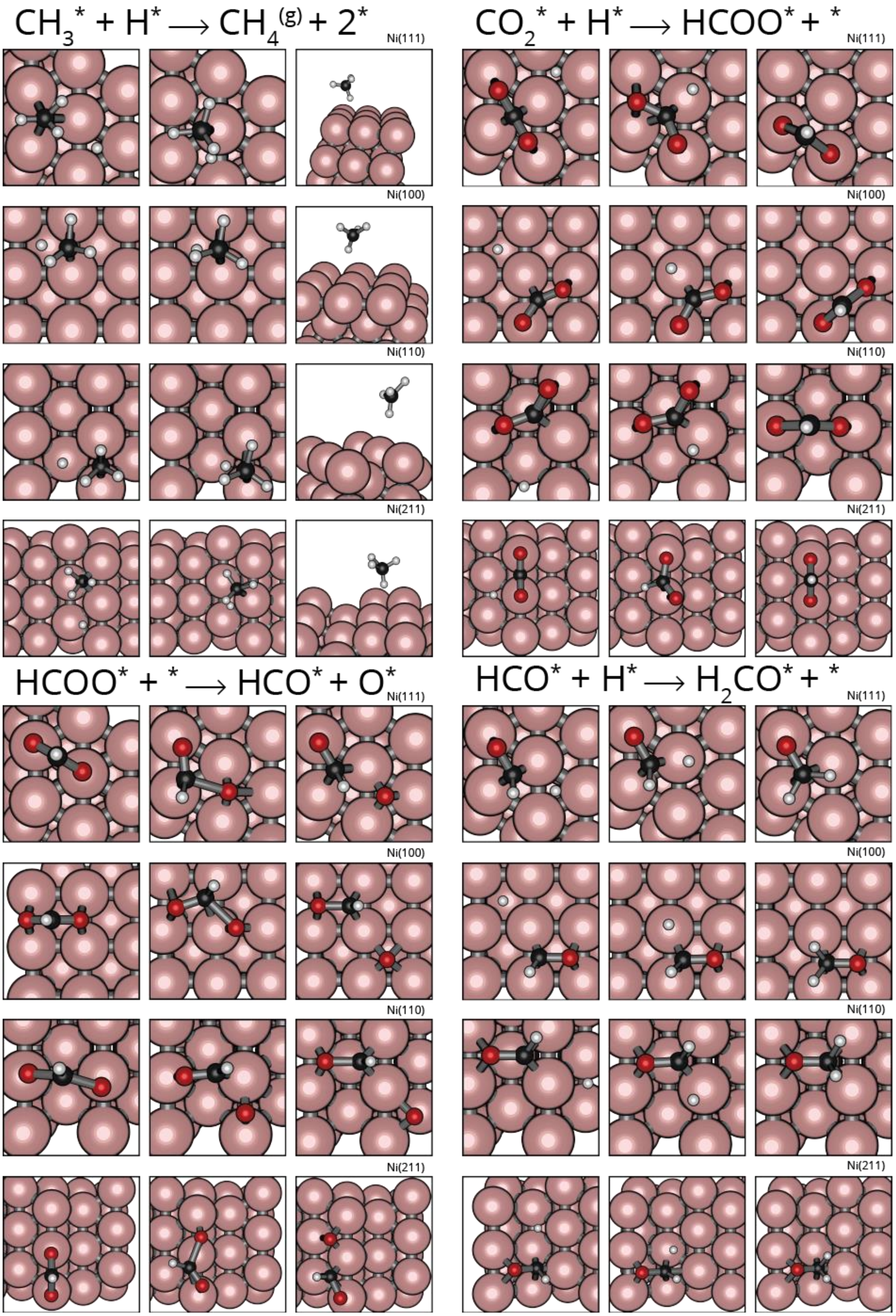
$CH_3^* + H^* \longrightarrow CH_4^{(g)} + 2^*$
Ni(111)
Ni(100)
Ni(110)
Ni(211)
$CO_2^* + H^* \longrightarrow HCOO^* + ^*$
Ni(111)
Ni(100)
Ni(110)
Ni(211)
$HCOO^* + ^* \longrightarrow HCO^* + O^*$
Ni(111)
Ni(100)
Ni(110)
Ni(211)
$HCO^* + H^* \longrightarrow H_2CO^* + ^*$
Ni(111)
Ni(100)
Ni(110)
Ni(211)

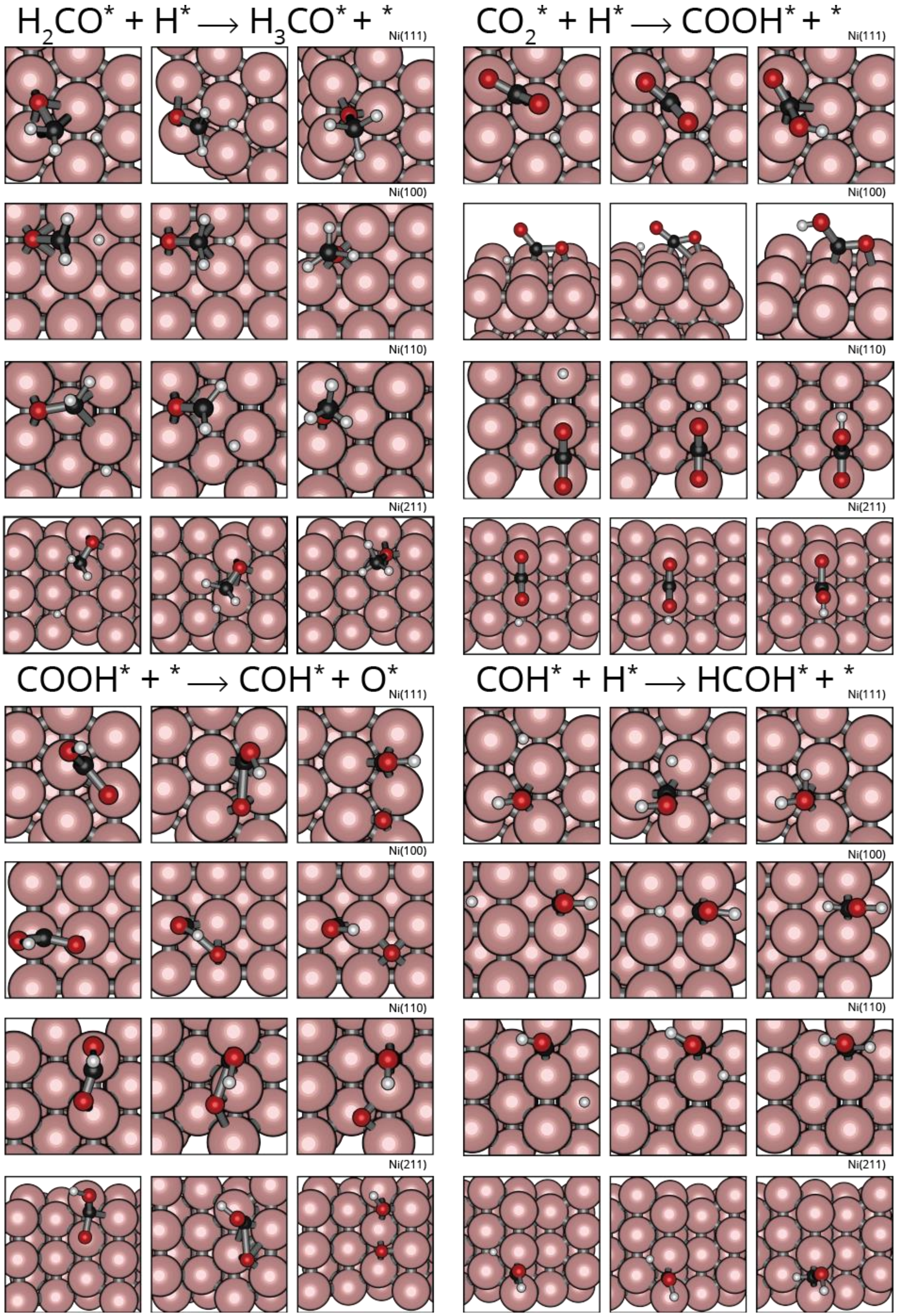
$H_2CO^* + H^* \longrightarrow H_3CO^* + {}^*$
Ni(111)
Ni(100)
Ni(110)
Ni(211)
$CO_2{}^* + H^* \longrightarrow COOH^* + {}^*$
Ni(111)
Ni(100)
Ni(110)
Ni(211)
$COOH^* + {}^* \longrightarrow COH^* + O^*$
Ni(111)
Ni(100)
Ni(110)
Ni(211)
$COH^* + H^* \longrightarrow HCOH^* + {}^*$
Ni(111)
Ni(100)
Ni(110)
Ni(211)

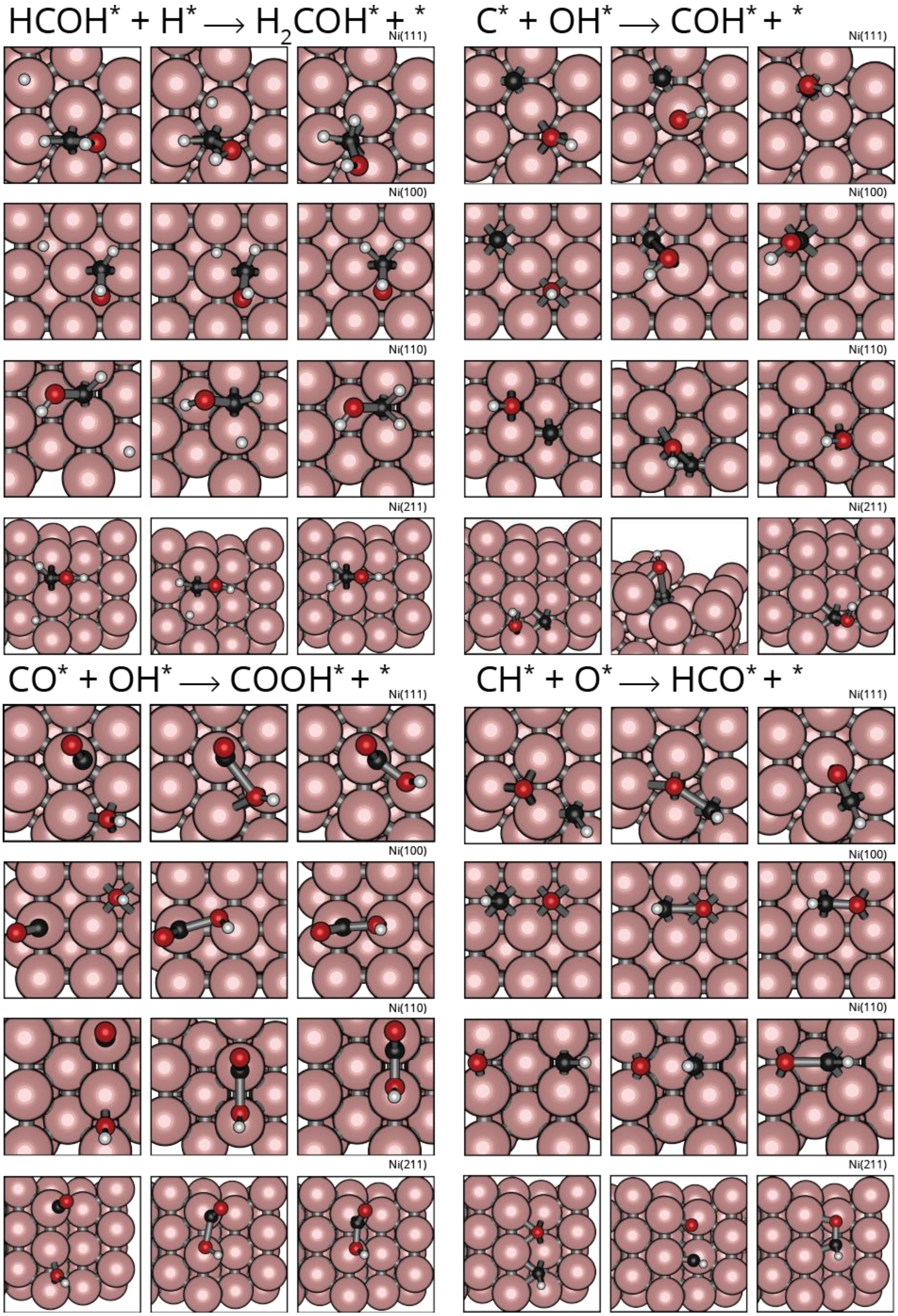
HCOH* + H* ⟶ H2COH* + *
Ni(111)
Ni(100)
Ni(110)
Ni(211)
C* + OH* ⟶ COH* + *
Ni(111)
Ni(100)
Ni(110)
Ni(211)
CO* + OH* ⟶ COOH* + *
Ni(111)
Ni(100)
Ni(110)
Ni(211)
CH* + O* ⟶ HCO* + *
Ni(111)
Ni(100)
Ni(110)
Ni(211)

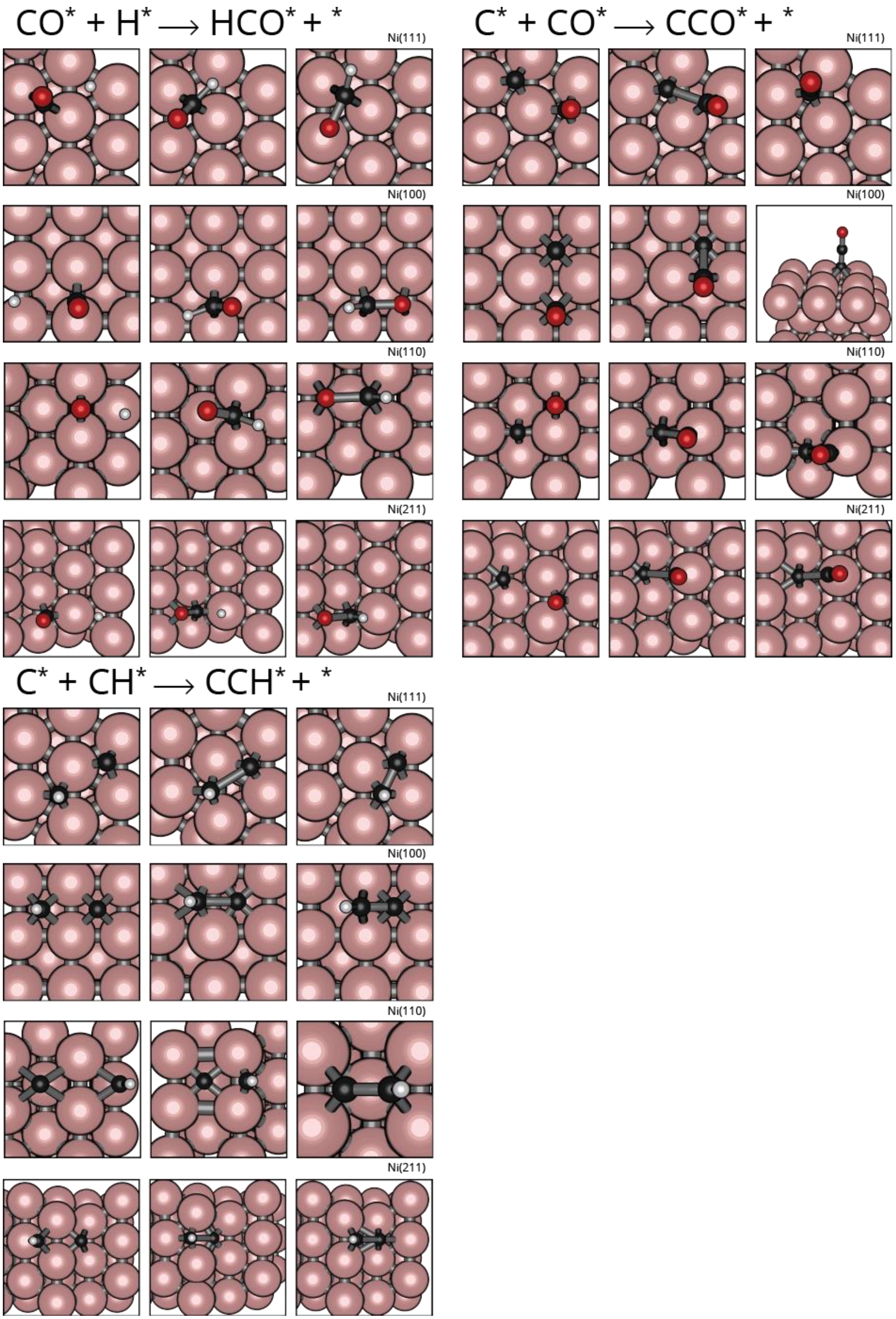

Supplementary Figure 21. The geometries of the initial- transition- and final states of the elementary reaction steps relevant in CO_2 hydrogenation over the 4 nickel facets (Ni(111), Ni(100), Ni(110), and Ni(211)) under study are shown.

Density of States Analysis CO_2* → CO* + O*

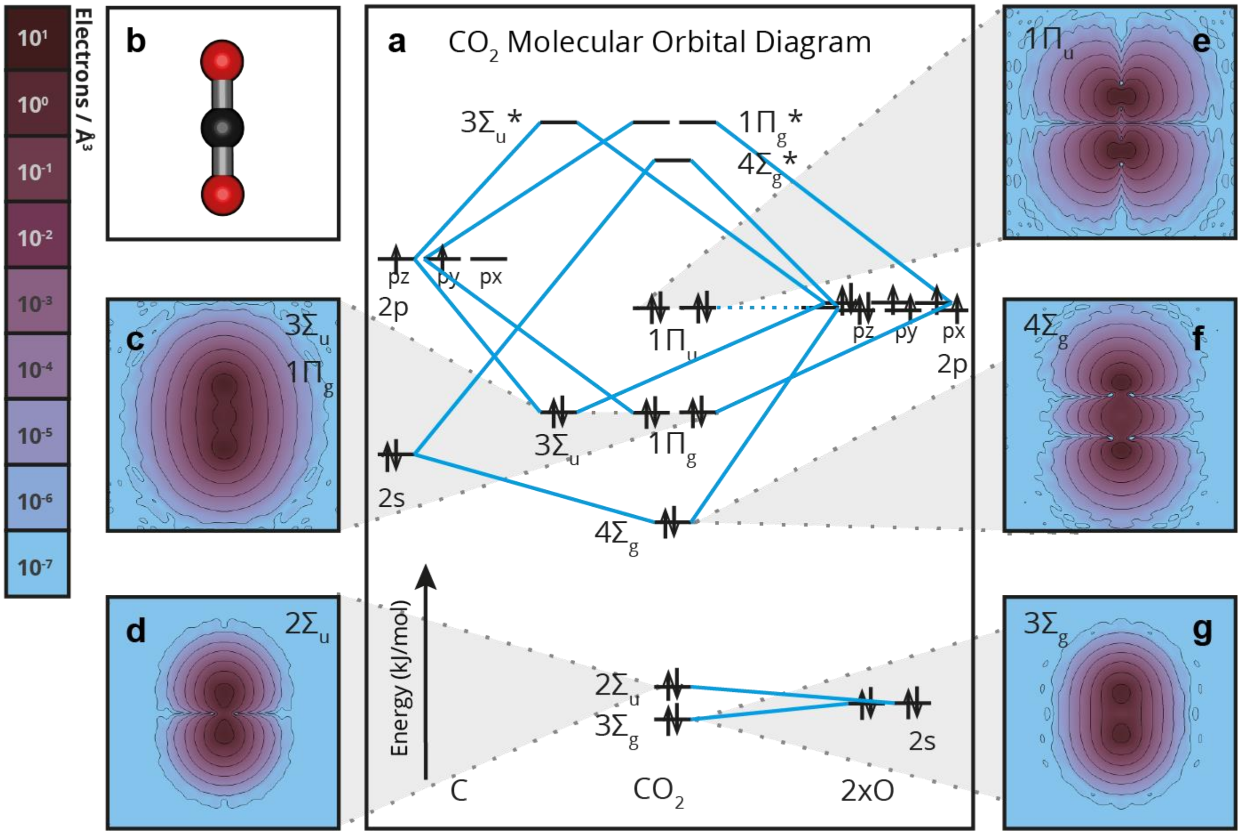

Supplementary Figure 22. a) Molecular orbital (MO) diagram for CO_2 by valence electrons, **b)** Orientation of CO_2 in images C-G, **c)** electron density of both $3\Sigma_u$ and $1\Pi_g$. The orbitals (as can be seen in A) or energy intervals lie too closely together to separate the contribution of the electron density of each and assign it to either. **d-g)** The electron density of each separate MO.

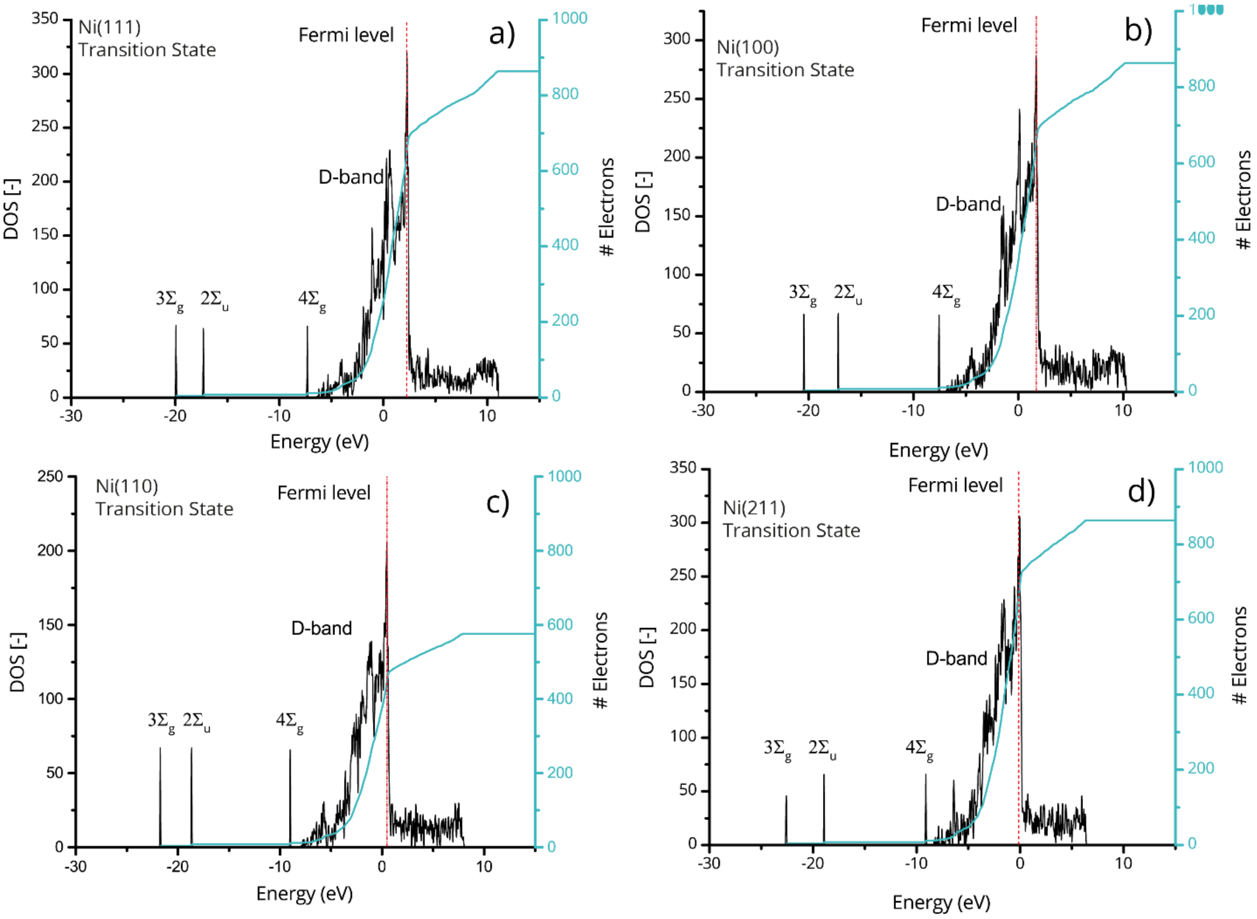

Supplementary Figure 23. Density of states analysis of (# = number of electrons) in the transition state of $CO_2^* \rightarrow CO^* + O^*$ ($CO_2^{\ddagger}$) on all four examined nickel facets a) Ni(111), b) Ni(100), c) Ni(110) and d) Ni(211).

Supplementary Table 5. Energy (in eV) of the bonding molecular orbitals 3Σg, 2Σu and 4Σg and the LUMO $4\Sigma_g^*$ of the transition state of CO_2* → CO* + O* ($CO_2^{‡}$) on the four examined nickel facets.

Molecular Orbital	111	100	110	211
$3\Sigma_g$	-22.64	-22.58	-22.64	-23.01
$2\Sigma_u$	-20.02	-19.31	-19.58	-19.34
$4\Sigma_g$	-10.02	-9.70	-9.93	-9.50
$4\Sigma_g^*$ LUMO	-6.29	-6.12	-5.77	-6.13

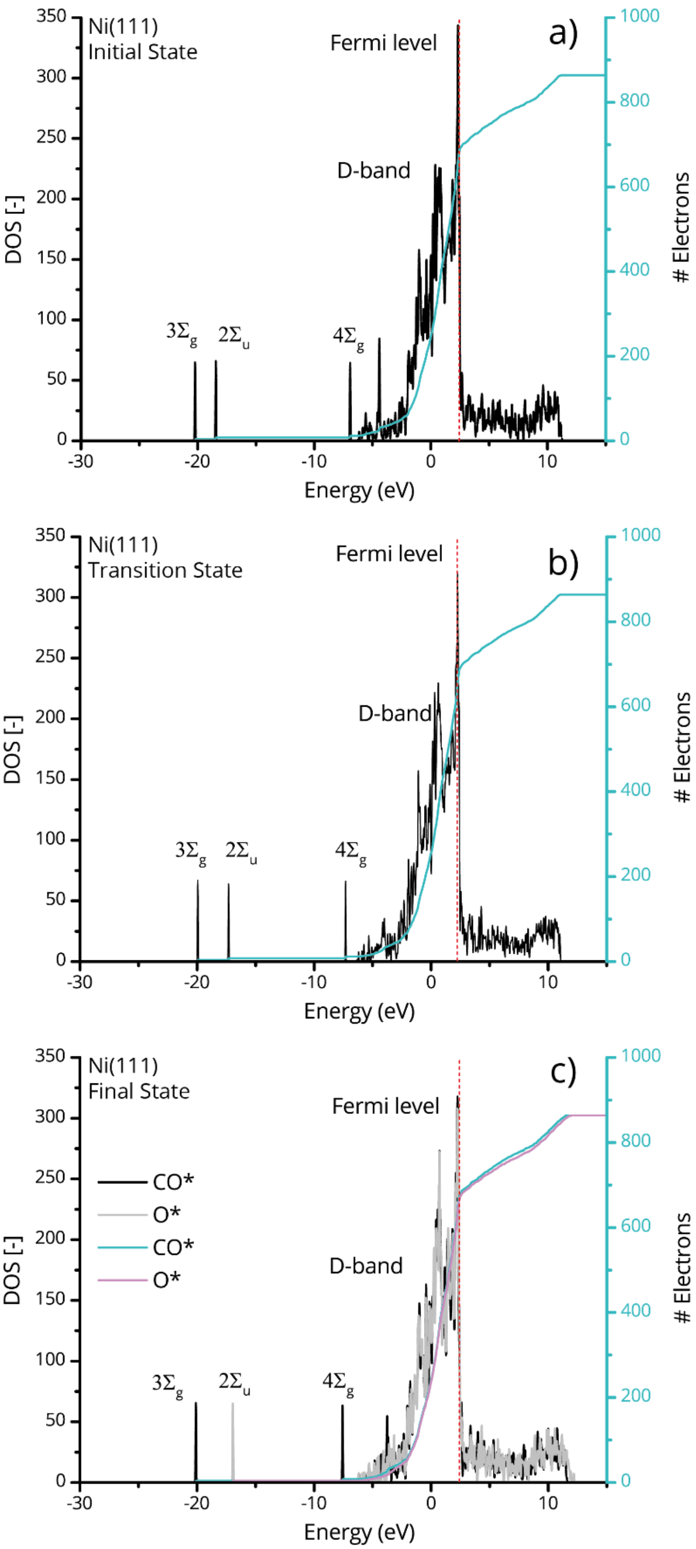

Supplementary Figure 24. Density of states analysis of CO_2* → CO* + O* on Ni(111) for a) the initial state, b) the transition state and c) the final state.

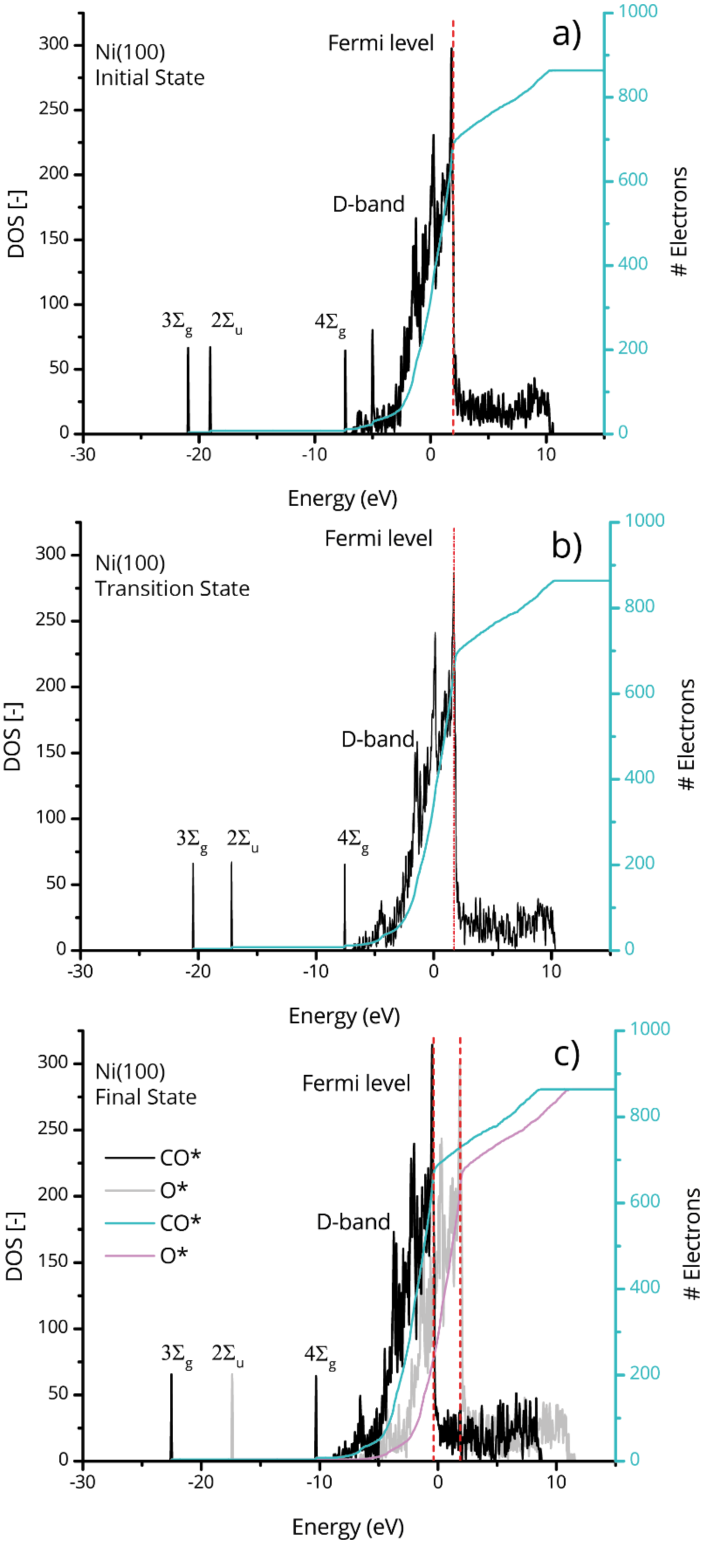

Supplementary Figure 25. Density of states analysis of CO_2* → CO* + O* on Ni(100) for a) the initial state, b) the transition state and c) the final state.

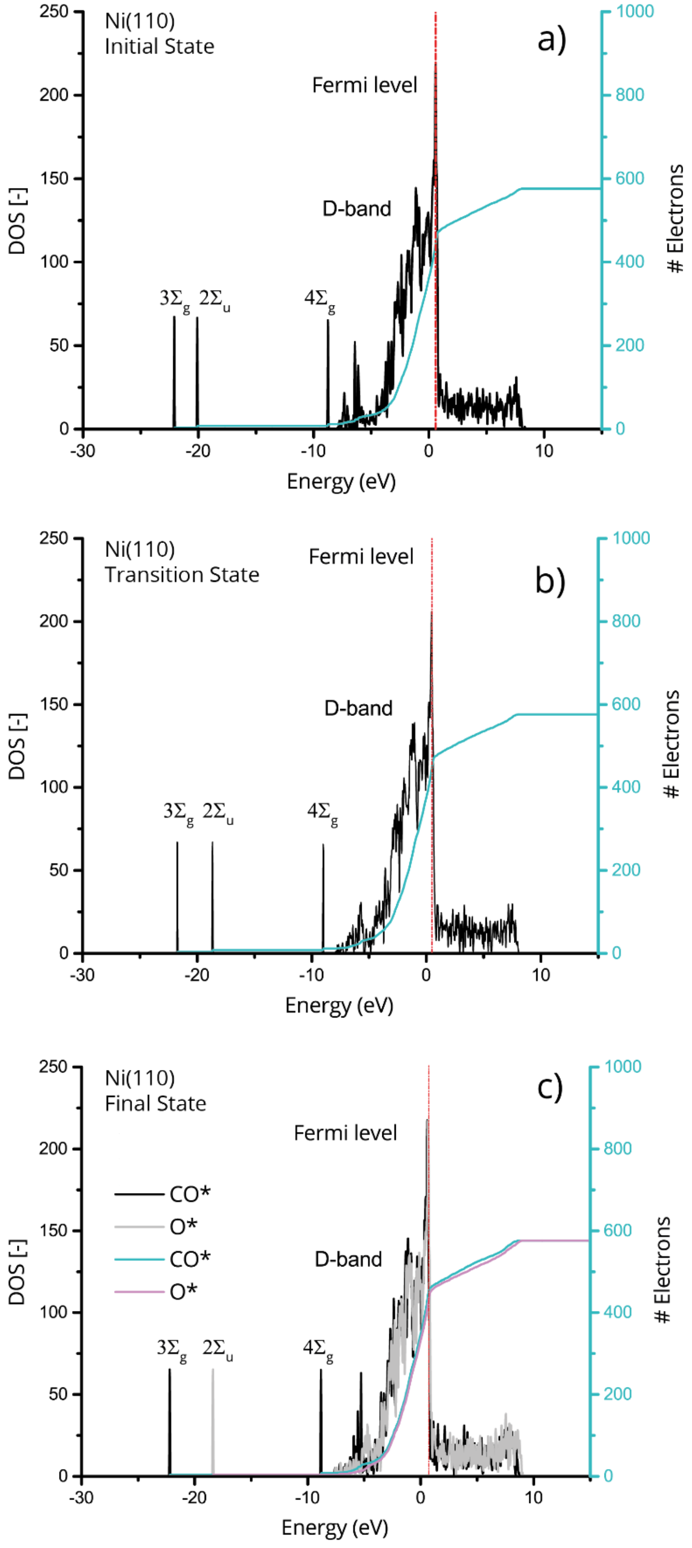

Figure 26. Density of states analysis of CO_2* → CO* + O* on Ni(110) for a) the initial state, b) the transition state and c) the final state.

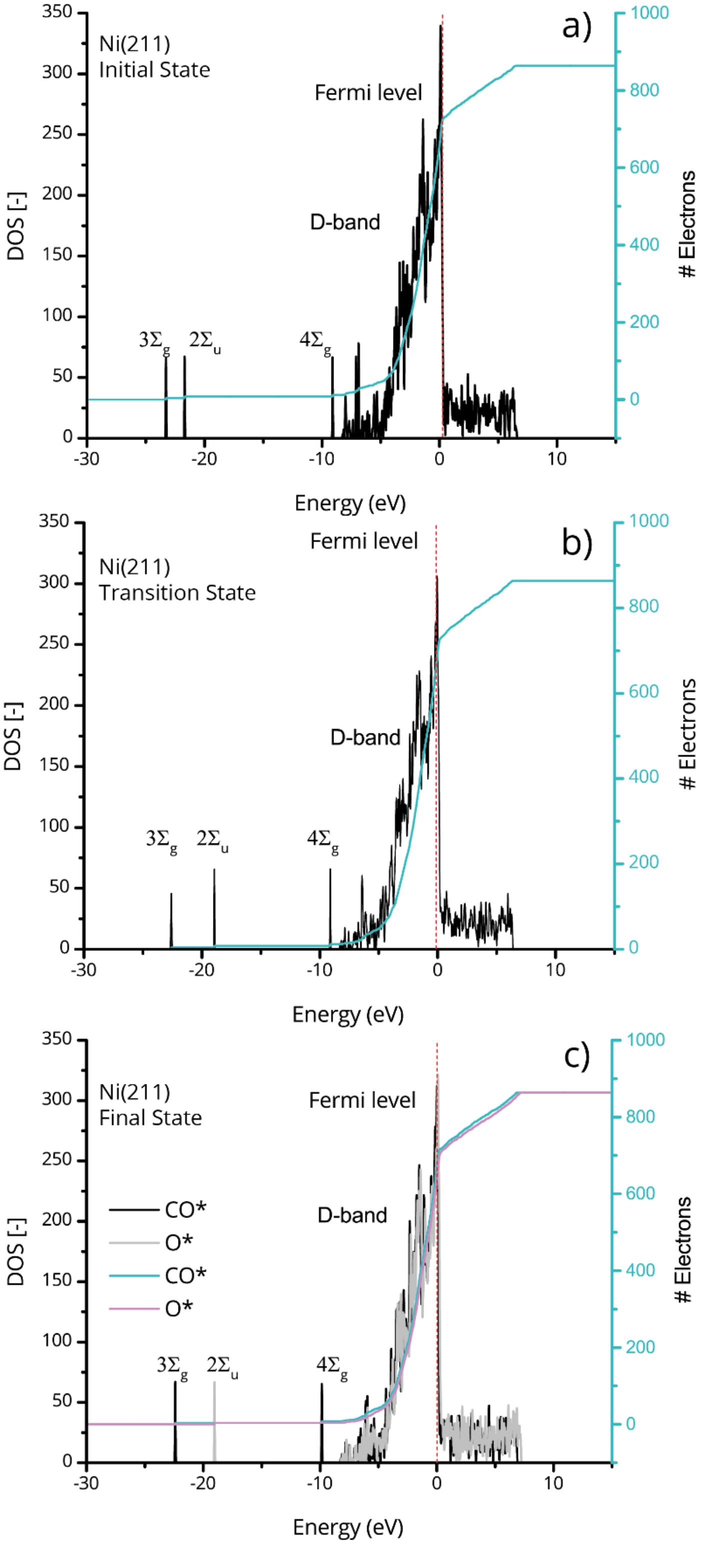

Figure 27. Density of states analysis of $CO_2^* \rightarrow CO^* + O^*$ on Ni(211) for a) the initial state, b) the transition state and c) the final state.

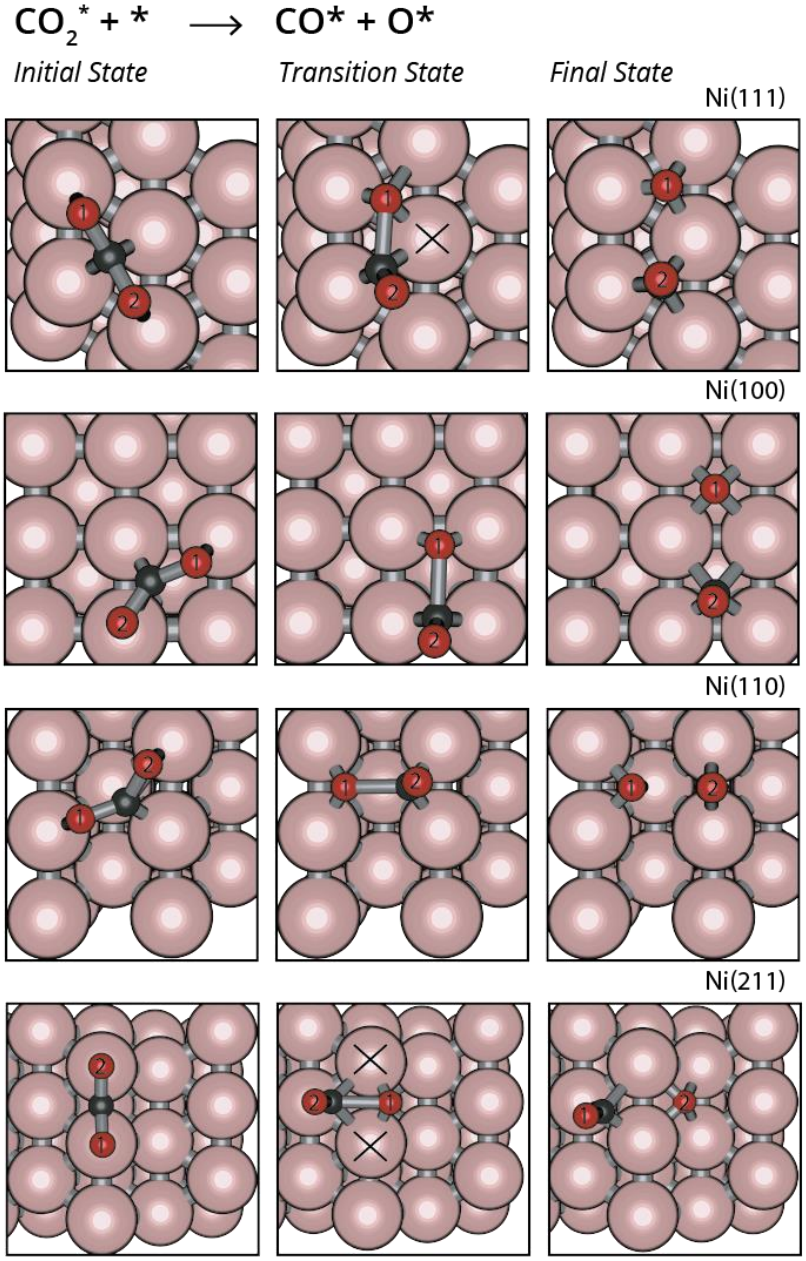

Supplementary Figure 28. Geometries of CO_2 on four nickel facets in the initial, transition and final state for direct CO_2 dissociation. The nickel atoms that are shared by the adsorbate in the transition state are indicated with a cross.

Supplementary Table 6. The preferred adsorption site, bond distances (d, in Å) and bond angle (θ, in °) of the adsorbates in the initial, transition, and final state. The coordination number (CN) of the transition state for direct CO_2 dissociation on the four nickel facets is also reported.

	Ni(111)	Ni(100)	Ni(110)	Ni(211)
Initial State				
Adsorption site OCO	T, B, T	T, B, T	T_1, T_1, T_1	T_1, B_1, T_1
d_{C-O1}	1.28	1.28	1.28	1.24
d_{C-O2}	1.28	1.27	1.28	1.24
θ_{OCO}	132.15	129.17	125.65	139.88
Transition State				
Adsorption site OCO	T_h, B	B, B	T_f, B_1	B_1, T_{h2}
CN	5	4	5	5
d_{C-O1}	1.68	1.82	1.78	1.83
d_{C-O2}	1.20	1.19	1.20	1.19
θ_{OCO}	117.20	111.03	110.51	112.45
Final State				
Adsorption site CO, O	T_h, T_h	F, F	B_1, T_f	T_{h2}, F
d_{C-O}	1.19	1.21	1.18	1.20

Microkinetic modelling

Supplementary Table 7. Summary of maximum CH_4 production, if $E_{app.}$ For CH_4 = 0. Per facet first a Sabatier Optimum was searched for in the temperature range of 400-1250 K. If the Sabatier Optimum could not be found, the temperature range was increased. The temperature for which the apparent activation barrier for CH_4 production is 0, is where the rate of methane production is highest. This temperature and speed were noted, and after this the surface coverage, CH_4 production, E_{app}, and finally the degree of rate control (DRC) in the window of interest (= 440 - 660 K) was evaluated. Summary of Most Abundant Reaction Intermediate (440-660 K) evaluated from coverage-graphs (see respective sections below).

	$E_{app.act\ for\ CH4\ prod}$ = 0 (mol/s)	MARI (440-660 K)	DRC (440-660 K)
**Ni(110)**	T=2250 K; CH_4 production = 0.17	CO*	HCO* + * → CH* + O*
**Ni(100)**	T=1650 K; CH_4 production = 10.4	H_2CO*	CH_2* + H* → CH_3* + *
**Ni(211)**	T=1650 K; CH_4 production = 0.63	CO*	CO* + * → C* + O*
**Ni(111)**	T = 2500 K; CH_4 production = 0.17	CO*	HCO* + * → CH* + O*

Supplementary Table 8. Summary of maximum CH_4 production inside operation window (440-660 K). E_{app} for CH_4 production inside operation window at T where CH_4 production is most active.

	$E_{app.act\ for\ CH4\ prod}$ [kJ/mol]	$T_{highest\ CH4\ production\ in\ 440\text{-}660K}$ [K]	Rate CH_4 production [mol/s]
**Ni(110)**	130	660	8.58e-5
**Ni(100)**	280	660	6.03e-6
**Ni(211)**	159	660	1.67e-2
**Ni(111)**	235	660	1.46e-5

<u>**Ni(111)**</u>

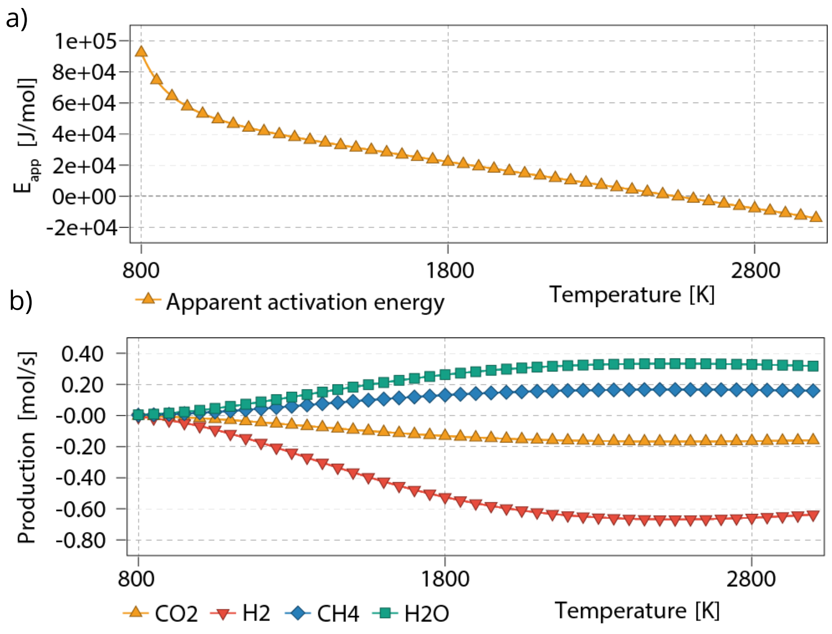

Supplementary Figure 29. Microkinetic modelling on a Ni(111) surface in a temperature range from 800-3000 K. A Sabatier Optimum is found at 2550 K, and maximum CH_4 production = 0.167 mol/s @ 2550 K. **a)** shows the apparent activation energy at a given temperature while **b)** shows the production rate in moles per second of different products.

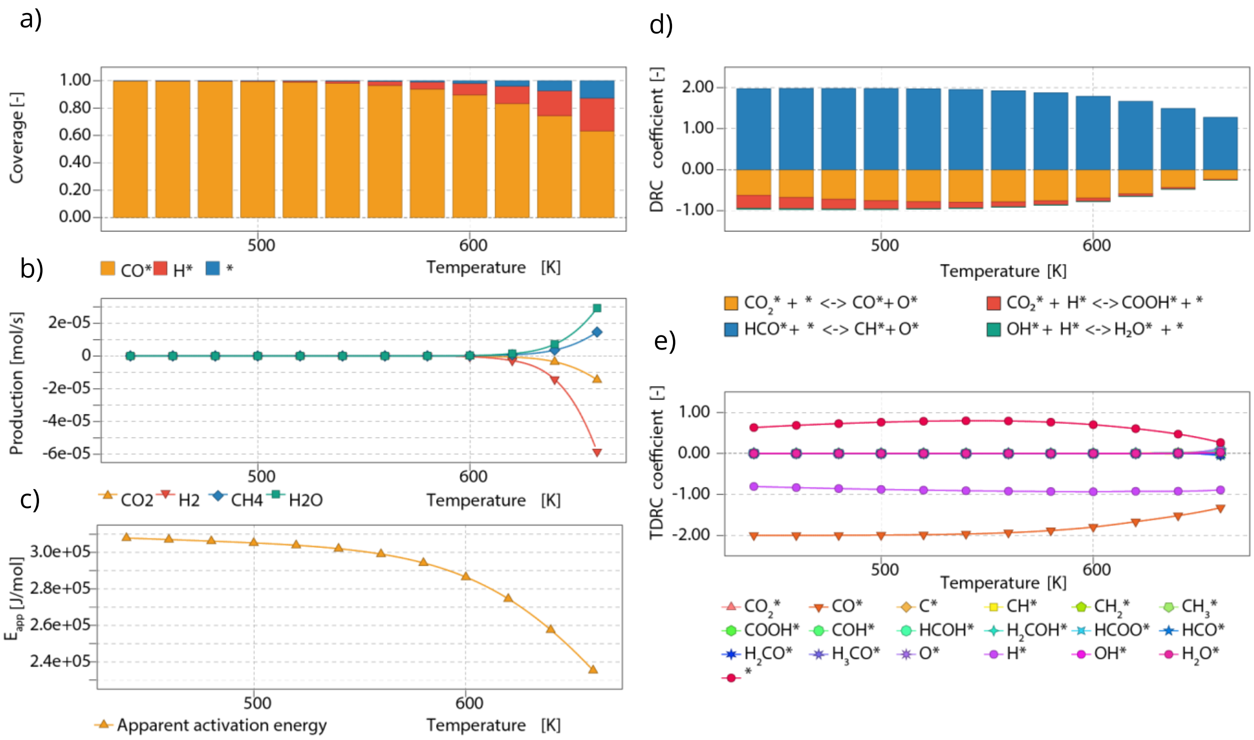

Supplementary Figure 30. Microkinetic modelling of individual reaction steps on a Ni(111) surface in a temperature range from 440-660 K. **a)** Coverage as a function of temperature, **b)** production rate of methane in mol/s, **c)** the apparent activation energy at different temperatures, **d)** degree of rate control (DRC) coefficient per reaction, **e)** the thermodynamic degree of rate control as a function of temperature.

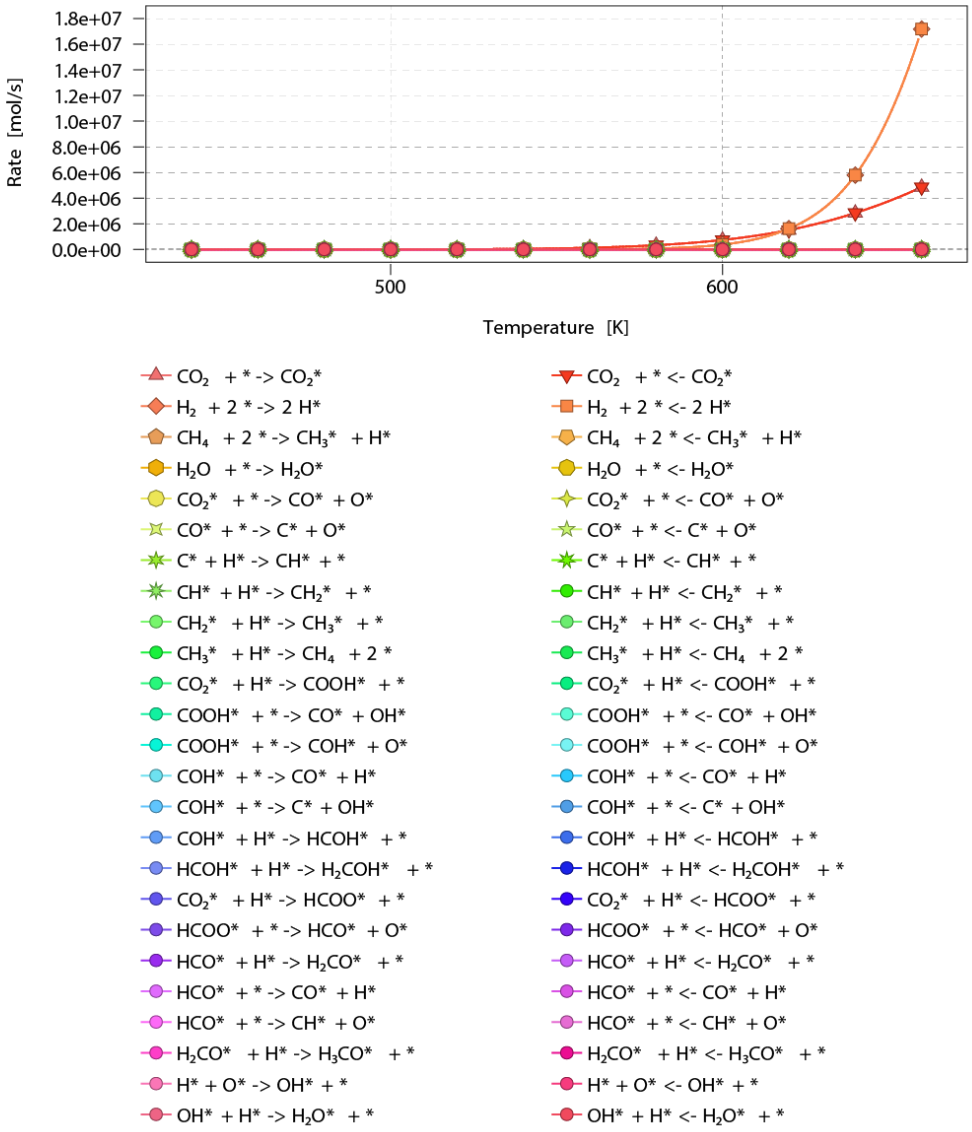

Supplementary Figure 31. Microkinetic modelling of individual reaction steps on a Ni(111) surface in a temperature range from 440-660 K, the rate in mol/s is shown for each reaction step.

Ni(110)

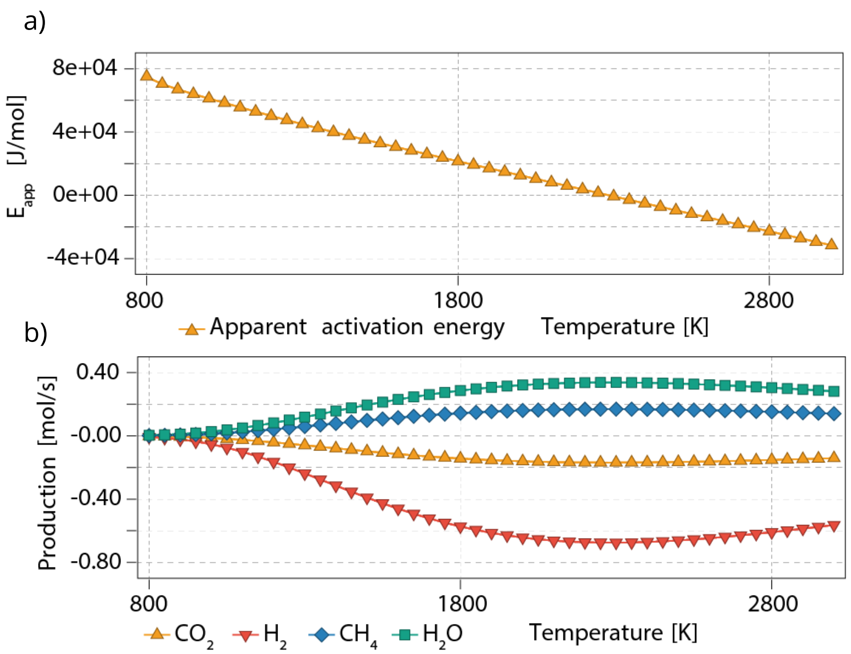

Supplementary Figure 32. Microkinetic modelling of individual reaction steps on a Ni(110) surface in a temperature range from 800-3000 K. Sabatier Optimum is found at 2250 K, and maximum CH_4 production = 0.169 mol/s @ 2250 K. **a)** shows the apparent activation energy at a given temperature while **b)** shows the production rate in moles per second of different products.

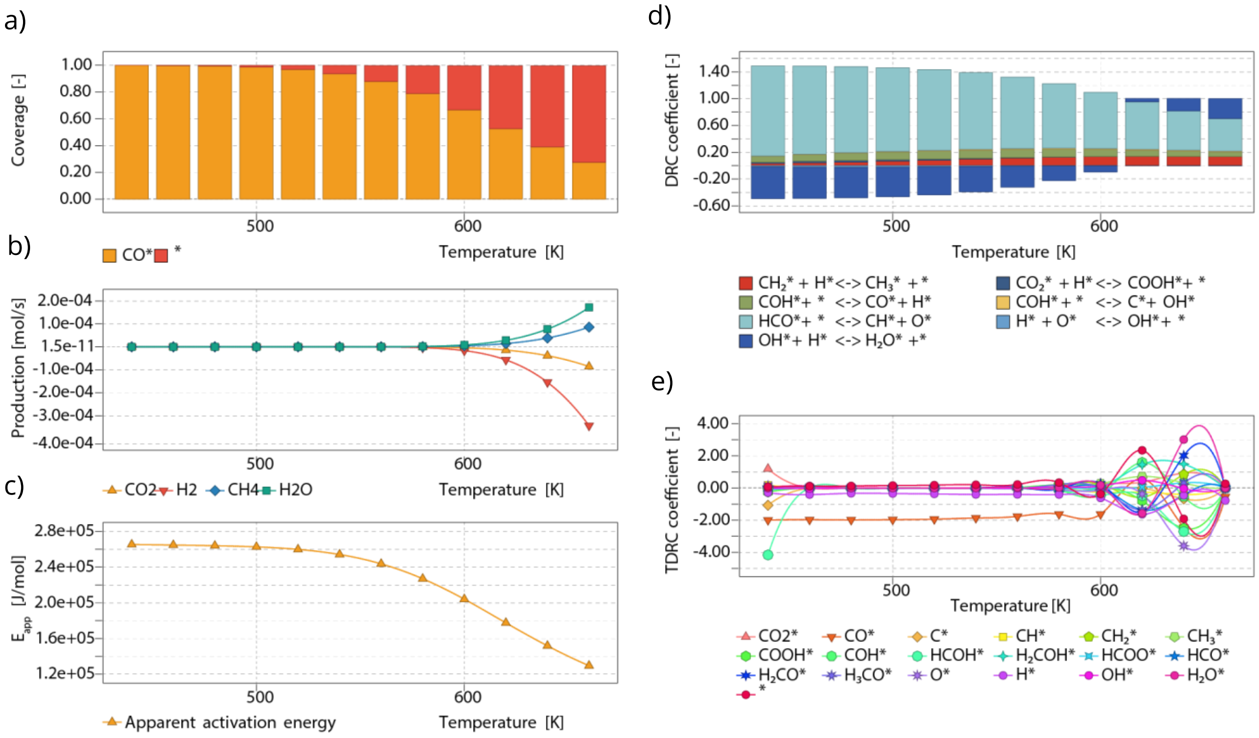

Supplementary Figure 33. Microkinetic modelling of individual reaction steps on a Ni(110) surface in a temperature range from 440-660 K. **a)** Coverage as a function of temperature, **b)** production rate of methane in mol/s, **c)** the apparent activation energy at different temperatures, **d)** degree of rate control (DRC) coefficient per reaction, **e)** the thermodynamic degree of rate control as a function of temperature.

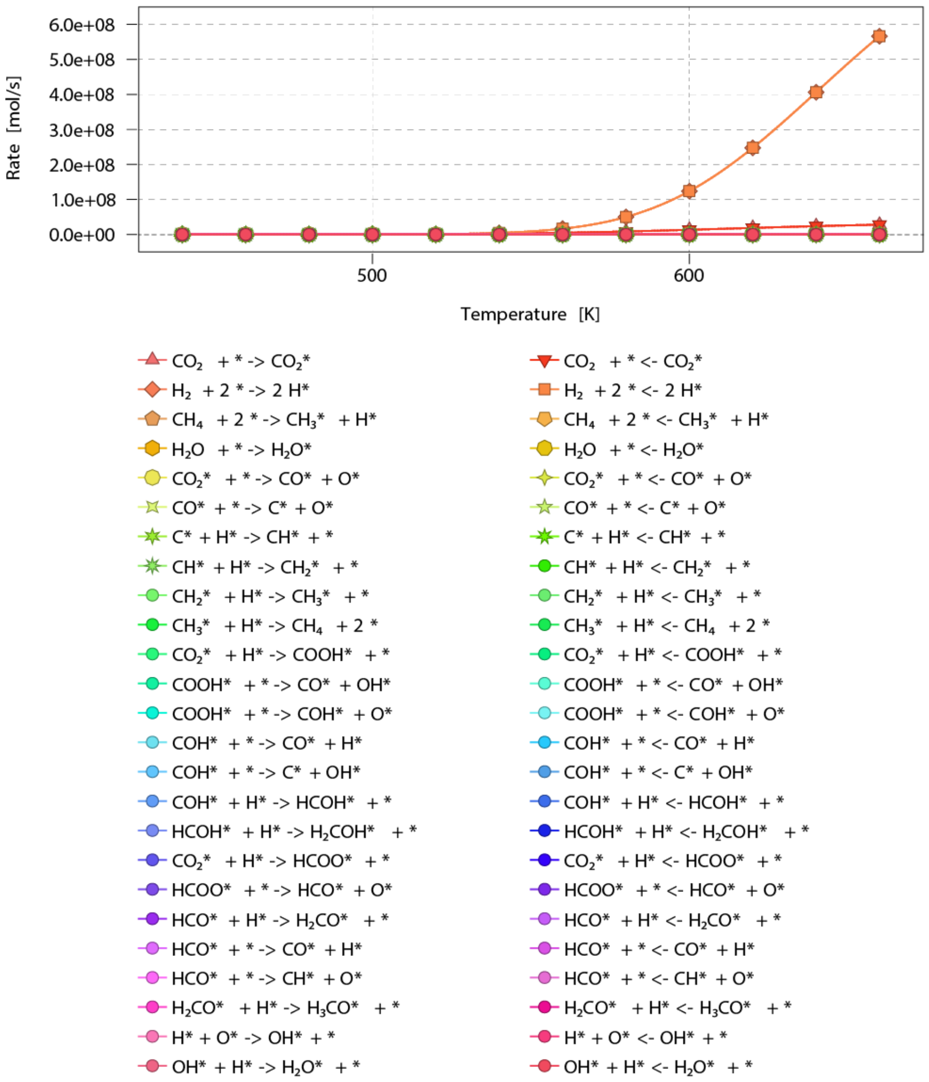

Supplementary Figure 34. Microkinetic modelling of individual reaction steps on a Ni(110) surface in a temperature range from 440-660 K, the rate in mol/s is shown for each reaction step.

Ni(100)

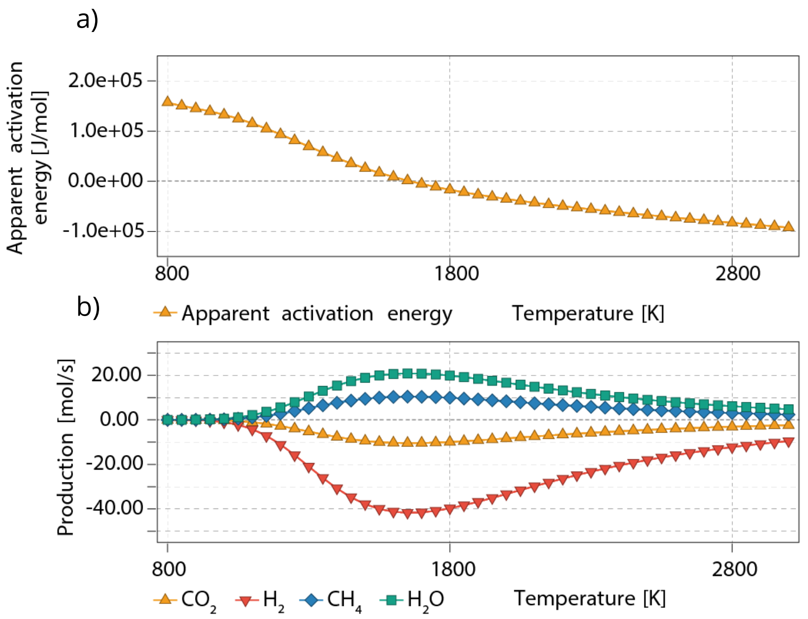

Supplementary Figure 35. Microkinetic modelling of individual reaction steps on a Ni(100) surface in a temperature range from 800-3000 K. Sabatier Optimum is found at 1650 K, and maximum CH_4 production = 10.4 mol/s @ 1650 K.

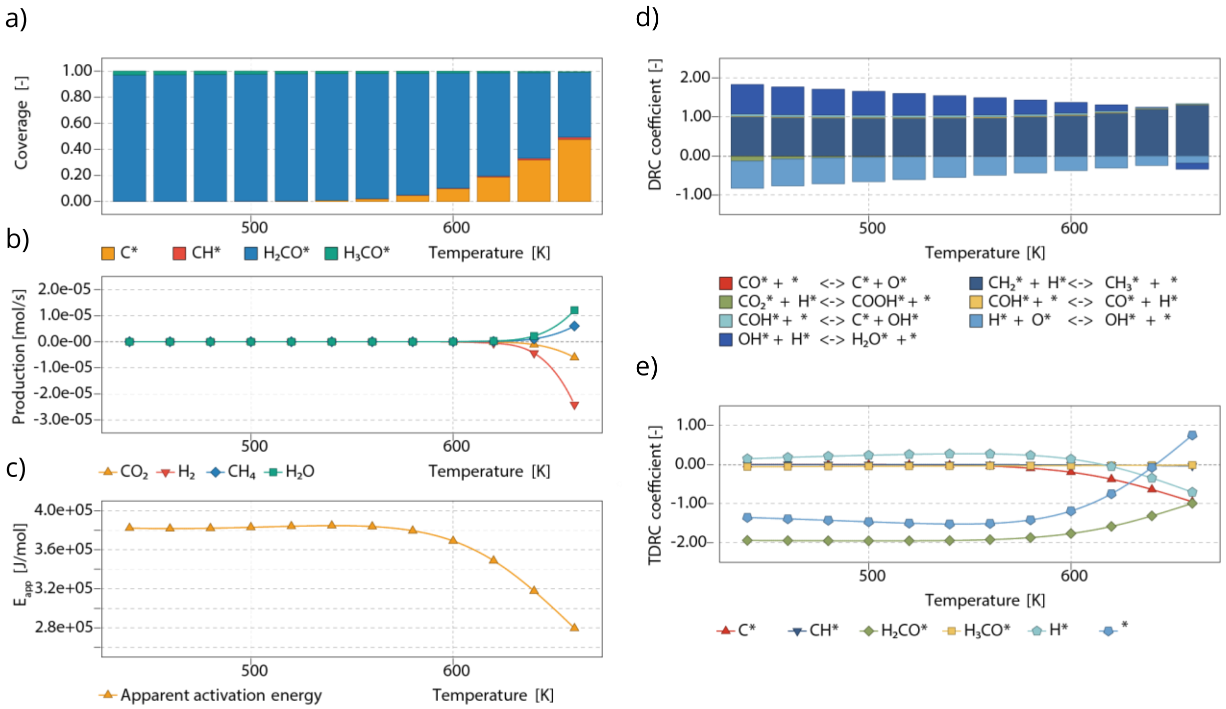

Supplementary Figure 36. Microkinetic modelling of individual reaction steps on a Ni(100) surface in a temperature range from 440-660 K. **a)** Coverage as a function of temperature, **b)** production rate of methane in mol/s, **c)** the apparent activation energy at different temperatures, **d)** degree of rate control (DRC) coefficient per reaction, **e)** the thermodynamic degree of rate control as a function of temperature.

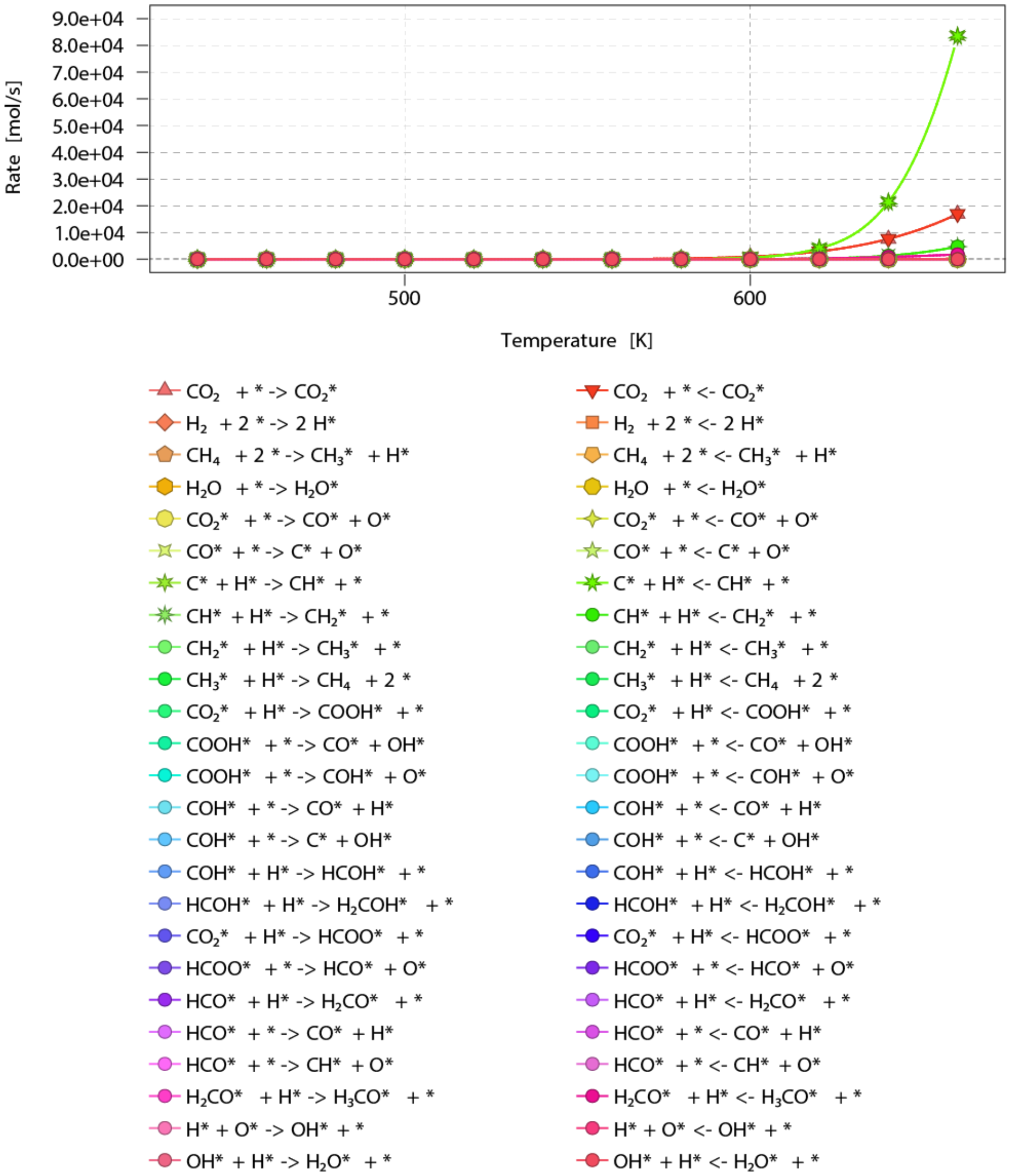

Supplementary Figure 37. Microkinetic modelling of individual reaction steps on a Ni(100) surface in a temperature range from 440-660 K, the rate in mol/s is shown for each reaction step.

Ni(211)

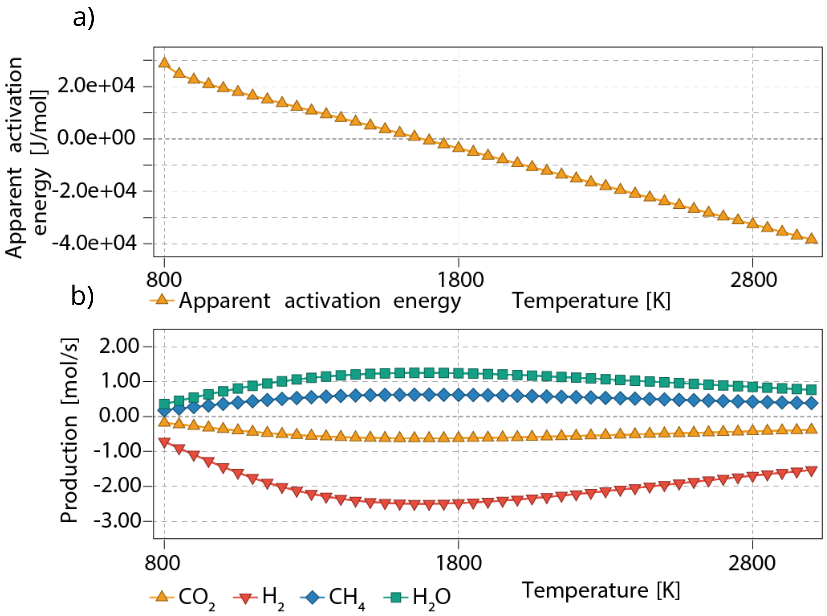

Supplementary Figure 38. Microkinetic modelling of individual reaction steps on a Ni(211) surface in a temperature range from 800-3000 K. Sabatier Optimum is found at 1650 K, and maximum CH_4 production = 0.625 mol/s @ 1650 K.

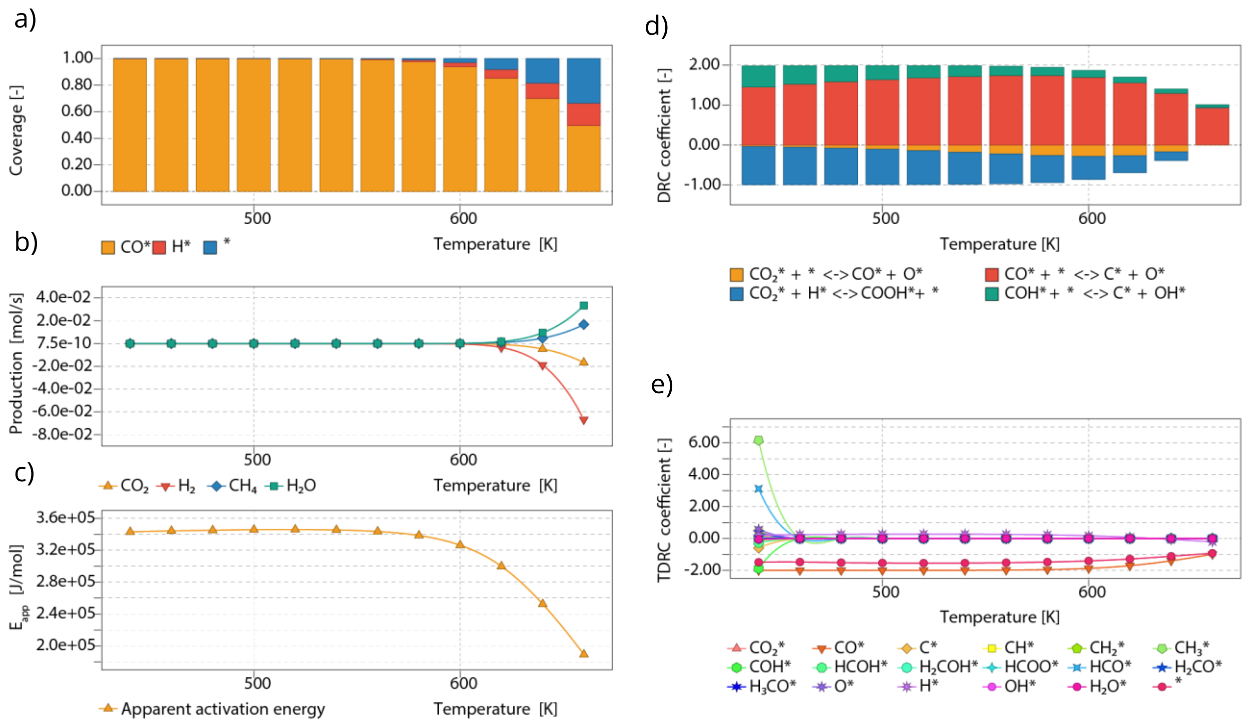

Supplementary Figure 39. Microkinetic modelling of individual reaction steps on a Ni(211) surface in a temperature range from 440-660 K. **A)** Coverage as a function of temperature, **B)** production rate of methane in mol/s, **C)** the apparent activation energy at different temperatures, **D)** degree of rate control (DRC) coefficient per reaction, **E)** the thermodynamic degree of rate control as a function of temperature.

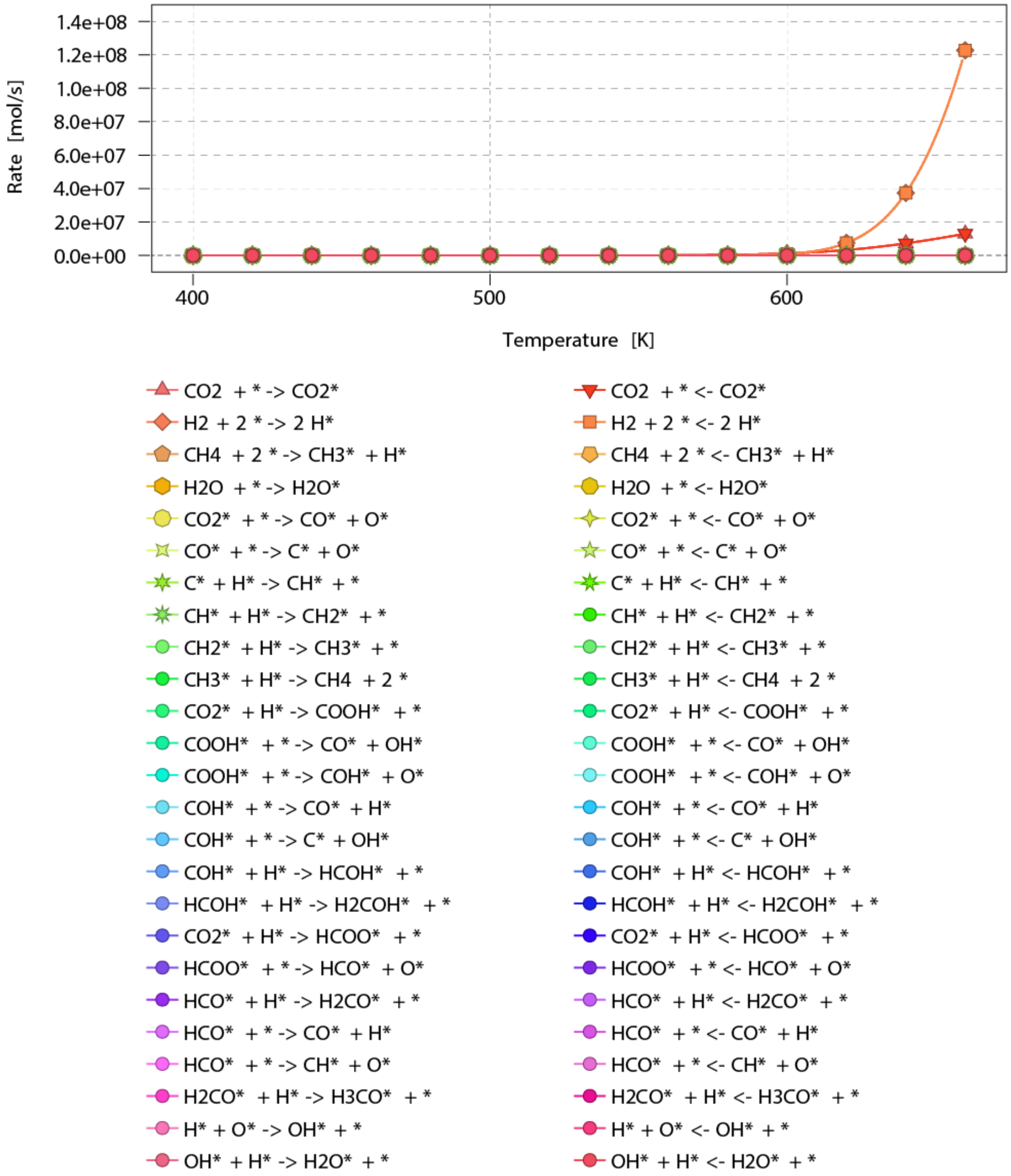

Supplementary Figure 40. Microkinetic modelling of individual reaction elementary steps on a Ni(211) surface in a temperature range from 400-660 K, the rate in mol/s is shown for each elementary reaction step.

Supplementary references

1. Vogt, C. *et al.* Unravelling structure sensitivity in CO_2 hydrogenation over nickel. *Nat. Catal.* **1**, 127–134 (2018).
2. Kleis, J. *et al.* Finite size effects in chemical bonding: From small clusters to solids. *Catal. Letters* **141**, 1067–1071 (2011).
3. Suchorski, Y. *et al.* The role of metal/oxide interfaces for long-range metal particle activation during CO oxidation. *Nat. Mater.* **17**, 519–522 (2018).
4. Lejaeghere, K. *et al.* Reproducibility in density functional theory calculations of solids. *Science* **351**, aad3000 (2016).
5. Vogiatzis, K. D. *et al.* Computational Approach to Molecular Catalysis by 3d Transition Metals: Challenges and Opportunities. *Chem. Rev.* **119**, 2453–2523 (2019).
6. Medvedev, M. G., Bushmarinov, I. S., Lyssenko, K. A., Sun, J. & Perdew, J. P. Density functional theory is straying from the path toward the exact functional. *Science* **355**, 49–52 (2017).
7. Mohr, S., Eixarch, M., Amsler, M., Mantsinen, M. J. & Genovese, L. Linear scaling DFT calculations for large tungsten systems using an optimized local basis. *Nucl. Mater. Energy* **15**, 64–70 (2018).
8. Wang, S., Cao, D., Li, Y., Wang, J. & Jiao, H. Chemisorption of CO_2 on Nickel Surfaces. *J. Phys. Chem. B* **109**, 18956–18963 (2005).
9. Luo, Q., Feng, G., Beller, M. & Jiao, H. Formic Acid Dehydrogenation on Ni(111) and Comparison with Pd(111) and Pt(111). *J. Phys. Chem. C* **116**, 4149–4156 (2012).
10. Vesselli, E. *et al.* Carbon Dioxide Hydrogenation on Ni(110). *J. Am. Chem. Soc.* **130**, 11417–11422 (2008).
11. Silaghi, M., Comas-Vives, A. & Copéret, C. CO_2 activation on Ni/$\gamma - Al_2O_3$ catalysts by first-principles calculations: from ideal surfaces to supported nanoparticles. *ACS Catal.* **6**, 4501–4505 (2016).
12. Cao, D.-B., Li, Y.-W., Wang, J. & Jiao, H. CO_2 dissociation on Ni(211). *Surf. Sci.* **603**, 2991–2998 (2009).
13. Zhou, M. & Liu, B. First-Principles Investigation of Adsorbate–Adsorbate Interactions on Ni(111), Ni(211), and Ni(100) Surfaces. *Ind. Eng. Chem. Res.* **56**, 5813–5820 (2017).
14. Ashwell, A. P. *et al.* Hydrogenation of CO to Methanol on Ni(110) through Subsurface Hydrogen. *J. Am. Chem. Soc.* **139**, 17582–17589 (2017).
15. Yang, K., Zhang, M. & Yu, Y. Direct versus hydrogen-assisted CO dissociation over stepped Ni and Ni3Fe surfaces: a computational investigation. *Phys. Chem. Chem. Phys.* **17**, 29616–29627 (2015).
16. Orita, H., Itoh, N. & Inada, Y. A comparison of CO adsorption on Pt(211), Ni(211), and Pd(211) surfaces using density functional theory. *Surf. Sci.* **571**, 161–172 (2004).
17. Zhu, Y., Chen, D., Zhou, X. & Yuan, W. DFT studies of dry reforming of methane on Ni catalyst. *Catal. Today* **148**, 260–267 (2009).
18. Zhou, Y. H., Lv, P. H. & Wang, G. C. DFT studies of methanol decomposition on Ni(1 0 0) surface: Compared with Ni(111) surface. *J. Mol. Catal. A Chem.* **258**, 203–215 (2006).
19. Catapan, R. C., Oliveira, A. A. M., Chen, Y. & Vlachos, D. G. DFT study of the water-gas shift reaction and coke formation on Ni(111) and Ni(211) surfaces. *J. Phys. Chem. C* **116**, 20281–20291 (2012).
20. Yang, H. & Whitten, J. L. Adsorption of Formyl on Ni(100). *Langmuir* **11**, 853–859 (1995).

21. Luo, Q., Wang, T., Beller, M. & Jiao, H. Hydrogen generation from formic acid decomposition on Ni(211), Pd(211) and Pt(211). *J. Mol. Catal. A Chem.* **379**, 169–177 (2013).
22. Mohsenzadeh, A., Bolton, K. & Richards, T. DFT study of the adsorption and dissociation of water on Ni(111), Ni(110) and Ni(100) surfaces. *Surf. Sci.* **627**, 1–10 (2014).
